# Inducing Social Self‐Sorting in Organic Cages To Tune The Shape of The Internal Cavity†

**DOI:** 10.1002/anie.202007571

**Published:** 2020-07-16

**Authors:** Valentina Abet, Filip T. Szczypiński, Marc A. Little, Valentina Santolini, Christopher D. Jones, Robert Evans, Craig Wilson, Xiaofeng Wu, Michael F. Thorne, Michael J. Bennison, Peng Cui, Andrew I. Cooper, Kim E. Jelfs, Anna G. Slater

**Affiliations:** ^1^ Department of Chemistry and Materials Innovation Factory University of Liverpool Crown Street Liverpool L69 7ZD UK; ^2^ Department of Chemistry Imperial College London Molecular Sciences Research Hub White City Campus London W12 0BZ UK; ^3^ Aston Institute of Materials Research, School of Engineering and Applied Science Aston University Birmingham B4 7ET UK

**Keywords:** cage compounds, molecular materials, multi-component self-assembly, self-sorting, supramolecular chemistry

## Abstract

Many interesting target guest molecules have low symmetry, yet most methods for synthesising hosts result in highly symmetrical capsules. Methods of generating lower symmetry pores are thus required to maximise the binding affinity in host–guest complexes. Herein, we use mixtures of tetraaldehyde building blocks with cyclohexanediamine to access low‐symmetry imine cages. Whether a low‐energy cage is isolated can be correctly predicted from the thermodynamic preference observed in computational models. The stability of the observed structures depends on the geometrical match of the aldehyde building blocks. One bent aldehyde stands out as unable to assemble into high‐symmetry cages‐and the same aldehyde generates low‐symmetry socially self‐sorted cages when combined with a linear aldehyde. We exploit this finding to synthesise a family of low‐symmetry cages containing heteroatoms, illustrating that pores of varying geometries and surface chemistries may be reliably accessed through computational prediction and self‐sorting.

## Graphical abstract

**A sea of expanding shapes**: A combined experimental–computational approach enabled the synthesis of low‐symmetry imine cages from mixtures of tetraaldehyde building blocks. This “social self‐sorting” approach was applied to obtain a family of new cages containing heteroatoms, showing that pores of varying geometries and surface chemistries may be reliably accessed.

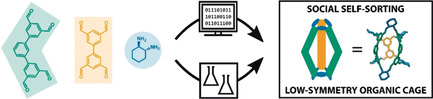

## Introduction

Controlled host‐guest recognition is of crucial importance to biological processes and artificial supramolecular systems alike.^1^, ^2^ Cage‐like compounds have been developed to exploit such host–guest interactions to achieve pollutant remediation,^3^ gas storage,^4^ anion binding,^5^ biomimetic guest recognition,^6^ molecular separations,^7^ and nanoparticle templation.^8^, ^9^ Advantages of organic cage hosts include their improved solubility over framework materials, making them excellent candidates for both liquid‐ or solid‐phase applications.^10^, ^11^, ^12^, ^13^, ^14^ Furthermore, cages offer synthetic handles that can be used to finely tune their cavity shape and electronic properties, and hence potential interactions with guest molecules.^7^, ^15^, ^16^, ^17^, ^18^, ^19^ However, the synthesis of molecular cages is often challenging, particularly where multiple bonds must be formed selectively. To avoid this problem, many molecular cage syntheses take advantage of dynamic covalent chemistry, in which reversible reactions provide an error correction mechanism to ensure the thermodynamic cage product is obtained.^20^, ^21^, ^22^ In the case of imine‐based cages, multiple amines and aldehydes must react to yield a single cage species instead of oligomeric mixtures of imines.^23^, ^24^, ^25^, ^26^ Ideally, high‐fidelity self‐sorting biases the formation of the cage product over the many other possible products, enabling selective isolation of the target molecule.^27^, ^28^, ^29^

A limitation of self‐sorting strategies is that they often result in the formation of highly symmetrical products.^22^, ^30^, ^31^ Reducing the symmetry of the host may induce anisotropy in the solid state, improve the binding of low‐symmetry guests, or enable more controlled and directional post‐synthetic modification.^32^, ^33^, ^34^, ^35^ For example, fine‐tuning of the cavity of an organic cage has been shown to afford precise control over the selectivity of the resultant solid‐state material.^7^ Stepwise syntheses exploiting orthogonal reactivities can afford low‐symmetry organic cages,^6^, ^36^, ^37^ but this limits the scalability of the resulting materials. Alternatively, low‐symmetry architectures may be obtained by purification of complex mixtures, but this is a laborious process and may be unachievable on a preparative scale due to reconfiguration of the desired products.^38^, ^39^, ^40^, ^41^ A recent study showed that low‐symmetry cages can be formed using a lower‐symmetry aldehyde precursor, but the presence of multiple structural isomers precluded the unambiguous characterisation of the cage products.^42^ We sought to avoid these problems by designing an alternative single‐step route to low‐symmetry imine‐based cages. We used mixtures of multiple aldehyde precursors with different geometries to investigate their self‐sorting behaviour, screening for combinations that led to the selective formation of low‐symmetry cages.

**Figure 1:**
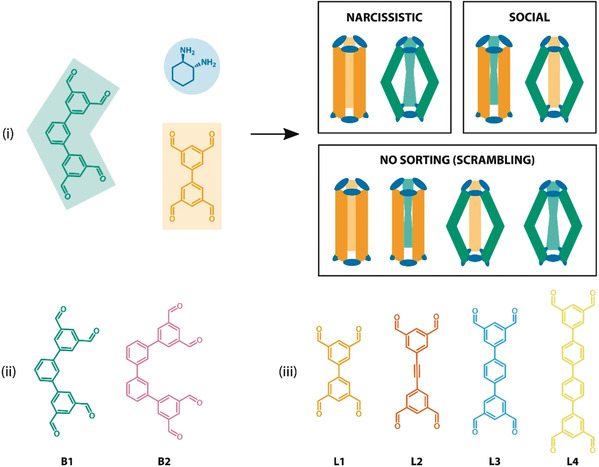
i) Illustration of narcissistically and socially self‐sorted systems as opposed to non‐sorted scrambled outcomes for an imine‐based organic cage forming reaction using linear (orange) and bent (green) aldehydes in presence of (1*R*,*2R)*‐*trans*‐1,2‐cyclohexanediamine (blue); ii) structures of the bent (**B1**, **B2**) and iii) linear (**L1–L4**) tetraaldehydes used in this work.

We recently reported a series of **Tet^3^Di^6^** tubular organic cages that were prepared through imine formation between three pseudo‐linear tetratopic aldehydes (“**Tet**”) and six ditopic amines (“**Di**”), and selected the linear tetraaldehydes as a starting point for these studies.^43^, ^44^ Reacting a mixture of two tetraaldehydes with a single diamine can produce three distinct sorting outcomes (see Figure 1 i): narcissistic self‐sorting, in which only cages incorporating a single aldehyde precursor are observed;^45^, ^46^, ^47^, ^48^ social self‐sorting, in which only cages incorporating both aldehyde precursors are observed;^27^, ^49^ and scrambling, in which a mixture of different sorting outcomes are observed.^50^ However, it is extremely hard to predict—either intuitively or computationally—which outcome will be observed for a given pair of aldehyde reactants. For simple cases, such as two linear aldehydes, one could expect that narcissistic self‐sorting is likely due to the mismatch in the aldehyde lengths and strain in the resultant cages.^48^, ^51^, ^52^, ^53^ It is much more difficult to predict the outcome when a linear aldehyde is combined with a bent aldehyde, such as **B1** (see Figure 1 ii). We hypothesised that the greater conformational degrees of freedom of non‐linear aldehydes compared to linear aldehydes would aid social self‐sorting or scrambling by accommodating a wider range of options for the cage geometry.^54^, ^55^, ^56^

To test our hypothesis, we studied imine‐based cages formed from two bent tetratopic aldehydes **B1–B2** and four linear aldehydes **L1–L4** of varying length (see Figure 1 iii). First, we sought to confirm that all the aldehydes individually form cages with (1*R*,2*R*)‐*trans*‐1,2‐cyclohexanediamine (*R*,*R*‐**CHDA**) in the presence of trifluoroacetic acid catalyst. Each bent aldehyde was then allowed to react sequentially with the series of linear aldehyde partners and *R*,*R*‐**CHDA** to assess their cage‐forming and self‐sorting behaviour. All reactions were characterised by ultra‐performance liquid chromatography‐mass spectrometry (UPLC‐MS) and ^1^H NMR spectroscopy. Where cage species could be isolated, crystal structures were sought to confirm their identities and assess their stable conformations. The sizes of isolable low‐symmetry cages were further investigated in solution by diffusion‐ordered spectroscopy (DOSY NMR) and ion‐mobility spectrometry‐mass spectrometry (IMS‐MS). Aldehyde **B1** was found to induce social self‐sorting in the studied cages, thus heteroatom‐containing analogues of **B1** were synthesised and reacted using the same methods to test whether the self‐sorting behaviour was retained.

We previously used density functional theory (DFT) formation energies to explain the thermodynamically preferred cage topologies in the dynamic imine‐based self‐assembly processes.^41^, ^57^, ^58^, ^59^ Comparing the thermodynamic stabilities of potential cage products can be a good guide to selectivity, but the reaction outcome can also be affected by factors, such as reaction kinetics,^39^, ^60^, ^61^, ^62^, ^63^, ^64^ solvent effects,^65^, ^66^, ^67^, ^68^ and the solubilities of the species involved in the equilibrium.^33^, ^40^, ^47^ In parallel with the synthetic efforts, we used computational techniques to predict the stability of the different homo‐ and heteroleptic structures originating from aldehydes **B1–B2** and **L1–L4**. The experimentally observed outcomes agreed with the relative gas‐phase formation energies of the possible **Tet^3^Di^6^** products, showing the predictive power of the simple model for the self‐sorting behaviour of imine‐based organic cages.

## Results and Discussion

### Single aldehyde systems

**Figure 2:**
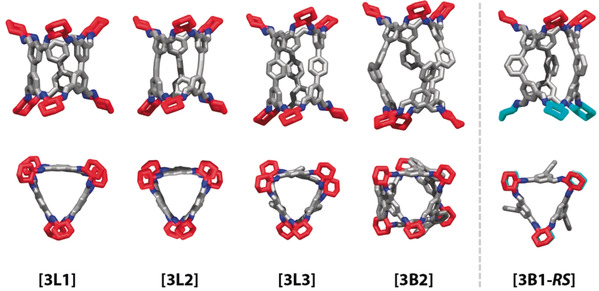
Side‐ and top‐views of the solvated single‐crystal X‐ray structures of the imine‐based cages originating from the reactions of tetraaldehydes **L1–L3** and **B2** (grey) with *R*,*R*‐**CHDA** (red). For **B1**, single crystals were only obtained for a pseudo‐*C*_3*h*_‐symmetric structure incorporating both *R*,*R‐* and *S*,*S*‐**CHDA** (cyan). Hydrogen atoms and solvent molecules are omitted for clarity, nitrogen atoms are in dark blue.

Aldehydes **B1–B2** and **L1–L4** were synthesised via Pd‐catalysed cross‐coupling reactions (see Supporting Information Section S3 for the synthetic details). The reactions of **L2** and **L3** with *R*,*R*‐**CHDA** have been reported to give tubular covalent cages **[3L2]** and **[3L3]**, respectively (see Figure 2 for the single‐crystal structures. Details and CCDC numbers are in the Supporting Information).^44^ The reaction of **L1** with *R*,*R*‐**CHDA** afforded a complex mixture of imine condensation products that could be purified by recrystallisation to yield **[3L1]**. Reactions of **L4** and **B2** with *R*,*R*‐**CHDA** both result in single cage products **[3L4]** and **[3B2]**, respectively. The single crystal structures of **[3L1]** and **[3B2]** could be elucidated. Unlike for the other aldehydes, reaction products of **B1** with either *R*,*R*‐ or *S*,*S*‐**CHDA** could not be identified and attempts to grow single crystals from such reaction mixtures were unsuccessful. However, co‐crystallisation of the opposite‐handed reaction products led to re‐equilibration of the building blocks and provided a pseudo‐*C*_3*h*_‐symmetric **[3B1‐*RS*]** cage. Investigation of the crystal structure of **[3B1‐*RS*]** revealed incorporation of equal amounts of each enantiomer of **CHDA** into the cage structure, which is reminiscent of the **CHDA** self‐sorting observed in a previously reported organic cage **CC3‐*RS***.^69^

This result prompted us to computationally explore the thermodynamic preference for the formation of pseudo‐*C*_3*h*_ cages incorporating both **CHDA** enantiomers against the corresponding enantiopure **Tet^3^Di^6^** cages (see Section S2 for the computational details). Indeed, the DFT formation energy for **[3B1‐*RS*]** was 20 kJ mol^−1^ lower than the formation energy for the enantiopure **[3B1]** at the M06‐2X/6–311G(3df,3pd) level of theory. **B1** was the only studied aldehyde for which any preference was observed. As the reaction of **B1** with *R*,*R*‐**CHDA** only formed the enantiopure **[3B1]** cage in trace amounts, we postulated that more stable cages may result from the addition of linear tetraaldehydes, enabling access to socially self‐sorted lower‐symmetry architectures. By contrast, cages including linear aldehydes and **B2** would compete with the favourable formation of **[3B2]**, leading to narcissistic self‐sorting or statistical mixtures of all possibilities.

### Mixed aldehyde systems

To explore self‐sorting in the system, we combined aldehydes from the **L** and **B** families in single‐pot reactions with *R*,*R*‐**CHDA** under cage‐forming conditions. We expected the **Tet^3^Di^6^** topology to be favoured in all cases based on our previous work.^44^ This assumption reduced the space of the possible structures to a number that could be systematically explored by computational methods. As imine formation is reversible under the reaction conditions used here, the observed product distributions are expected to relate to the thermodynamic minima. Therefore, formation energies can be predictive of the range of products seen. If all the possible cages have similar formation energies, we predict that multiple cage products will be formed or that the self‐sorted products will be selected by solvation and entropic effects. Conversely, if one or more cages are much lower in energy than the other possibilities, we predict that those structures will dominate the product distribution.

For each reaction studied, we manually constructed the **Tet^3^Di^6^** cages in all possible stoichiometries of the linear and bent aldehydes. We then applied a workflow consisting of high‐temperature molecular dynamics simulations with OPLS3e force field,^70^ followed by further geometry optimisations at the PBE‐GD3/TZVP‐MOLOPT‐GTH level of theory^71^, ^72^, ^73^, ^74^, ^75^, ^76^, ^77^, ^78^ to find the expected gas‐phase conformations of the resulting cages (see Section S3 for more details). Single point energies were calculated for the modelled structures at the M06‐2X/6–311G(3df, 3dp) level of theory and the resulting formation energies are summarised in Table 1 (for **B1**) and Table 2 (for **B2**). These calculations are performed on isolated molecules in the gas phase, which does not consider solvent effects, and hence large energetic differences are needed to predict solution‐phase structures with confidence.

**Table 1:** Side‐ and top‐views of the DFT‐optimised structures (PBE‐GD3/TZVP‐MOLOPT‐GTH) of the possible **Tet^3^Di^6^** outcomes for the reactions using mixtures of **B1** and **L1–L4** under cage forming conditions. Underneath are the single point formation energies (M06‐2X/6–311G(3df, 3dp)) in kJ mol^−1^. Entries marked with asterisks are the experimentally observed outcomes. Building blocks are coloured according to Figure 1, nitrogen atoms are dark blue, hydrogen atoms are omitted.

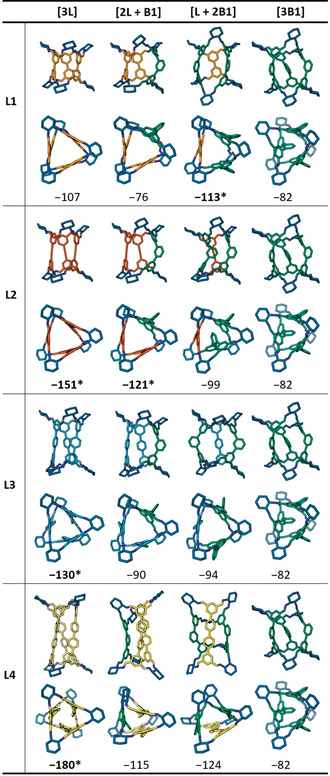

**Table 2:** Side‐ and top‐views of the DFT‐optimised structures (PBE‐GD3/TZVP‐MOLOPT‐GTH) of the possible **Tet^3^Di^6^** outcomes for the reactions using mixtures of **B2** and **L1–L4** under cage forming conditions. Underneath are the single point formation energies (M06‐2X/6–311G(3df, 3dp)) in kJ mol^−1^. Entries marked with asterisks are the experimentally observed outcomes. Building blocks are coloured according to Figure 1, nitrogen atoms are dark blue, hydrogen atoms are omitted.

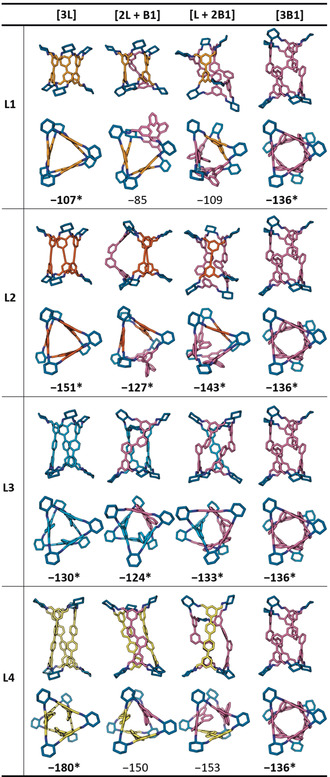

In parallel, we attempted to address the problem synthetically by targeting the cage stoichiometry of **[L+2B]** (+ 6 *R*,*R*‐**CHDA** omitted for clarity). A deuterated variant of aldehyde **L3** was available from a previous study and used in reactions with **B1** to allow discrimination of the resultant cages by mass in the UPLC‐MS.^79^ No such deuterated analogue was available for the mixture of **L4** and **B2**, and the reaction was characterised primarily by ^1^H NMR chemical shifts and UPLC retention times. Table 1 and Table 2 summarise which structures were experimentally observed.

Reactions of **B1** with aldehydes **L1‐4** and *R*,*R*‐**CHDA** all resulted in cage compounds corresponding to entries marked with asterisks in Table 1 (see Section S3.4 for screening details and raw spectra). For the shortest linear aldehyde **L1**, the major cage product was a pseudo‐*D*_3_ low‐symmetry **[L1 + 2B1]** cage, which was readily isolated via recrystallisation. The structure of this compound was elucidated by single‐crystal X‐ray diffraction (see Figure 4) and was the lowest energy structure predicted for that system. When the elongated **L2** was used instead, the two major products observed by UPLC‐MS were a low‐symmetry **[2L2 + B1]** cage and the previously described homoleptic **[3L2]**, which again were the two lowest‐energy predicted structures. For the even longer aldehyde **L3‐*d***, a complex mixture was observed by UPLC‐MS (see Figures S16–S21 and Tables S3,S4). The major product was identified as the **[3L3‐*d*]** cage, in agreement with the computational models. Lower‐mass peaks corresponding to **[2L3‐*d* + B1]** and **[L3‐*d* + 2B1]** could also be detected, both structures being of comparable DFT formation energies. The relative proportions of the products could not be determined due to insufficient chromatographic separations. For the longest aldehyde trialled, **L4**, the major product was the symmetrical tubular **[3L4]** cage, which was of significantly lower DFT energy than any competing structure in this system. In all cases, other species could be detected by mass spectrometry as trace products, including the chiral cage **[3B1]**, but could not be isolated. The distribution of the products is affected by the length of the linear aldehydes **L1–L4** in a seemingly unpredictable way, but the observed structures agree with predicted trends in the DFT formation energies. The length of **L1** appears to be suitable to relieve strain in a cage containing two **B1** moieties; **L2** is of suitable length to relieve strain in a cage containing one **B1** moiety, but **L3** and **L4** seem to be too long and do not form stable mixed **Tet^3^Di^6^** cages with **B1**.

Reactions involving aldehyde **B2** and aldehydes **L1–L4** also all resulted in the formation of cage compounds (see Table 2 and Section S3.4). For aldehydes **L2** and **L3**, the outcomes of the reactions were scrambled and all possible **Tet^3^Di^6^** cages were observed, which were all of comparable DFT formation energies. For **L1** and **L4**, we observed narcissistic self‐sorting. In the case of **L4**, signals corresponding to **[3B2]** and **[3L4]** can be seen by ^1^H NMR spectroscopy and two clear peaks with retention times corresponding to **[3B2]** and **[3L4]** can be seen in the chromatogram (see Figures S22–S26 and Table S5). However, as all products in this system have the same mass, it was not possible to unambiguously characterise the self‐sorting behaviour with mass spectrometry. While formation energies of **[3L1]** and **[L1 + 2B2]** are comparable, and the formation energy of **[3L4]** is higher than that of the socially‐sorted cages **[*n*L4 +** ***m*** **B2]**, we propose that the clean narcissistic self‐sorting in those cases is a result of antagonistic coupling between the homoleptic and the heteroleptic cages in these libraries.^80^ Formation of the stable **[3B2]** cage removes free **B2** from solution, thus favouring the formation of **[3L1]** over **[L1 + 2B2]**. Similarly, formation of the significantly more stable **[3L2]** cage removes free **L2** from the system, thus shifting the equilibrium towards clean formation of **[3B2]** over the **[*n*L4 +** ***m*** **B2]** structures.

Contrary to **B1**, aldehyde **B2** has more degrees of freedom and forms a stable **[3B2]** cage as well as scrambled mixtures of heteroleptic cages with other aldehydes. Chromatographic separations of the mixtures containing heteroleptic cages **[2L2 + B1]**, **[*n*L2 +** ***m*** **B2]**, and **[*n*L3 +** ***m*** **B2]** are currently under investigation. The reaction of **L1** and **B1** with *R*,*R*‐**CHDA** stands out as the only combination which produces a low‐symmetry heteroleptic organic cage as the only product detected by NMR.

### Heteroatom‐containing [L1 + 2B1X] systems

**Figure 3:**
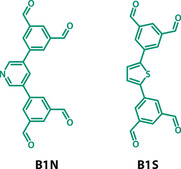
Chemical structures of heteroatom‐containing aldehydes **B1N** and **B1S**.

To investigate whether social self‐sorting would also be observed with analogues of **B1** we synthesised thiophene (**B1S**) and pyridyl (**B1N**) derivatives that have structurally related geometries (see Figure 3 for the chemical structures and Section S3.2 for the synthetic details). Due to the incorporation of heteroatoms and differently sized rings in their cores, these aldehydes were expected to produce cages with different pore geometries and electronic properties.^48^, ^81^, ^82^

**Figure 4:**
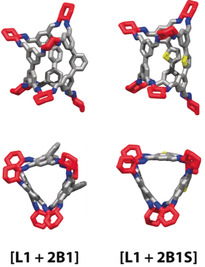
Side‐ and top‐views of the single‐crystal X‐ray structures of the socially sorted **[L1 + 2B1X]** cages originating from the reactions of mixtures of tetraaldehydes (grey) **L1** and **B1** (left) or **B1S** (right) with *R*,*R*‐**CHDA** (red).

In both cases, the reactions of **B1S** and **B1N** with **L1** and *R*,*R*‐**CHDA** gave analogues of **[L1 + 2B1]** as the major product, accompanied by significant formation of the corresponding homoleptic **[3B1X]** cages (see Section S3.5 for the synthetic details). Computational modelling predicts comparable formation energies for **[L1 + 2B1X]** and **[3B1X]** in both cases, with a slight preference for the heteroleptic structures (see Section S2 for the computational details). Inspection of the computational models suggests that **[L1 + 2B1]** and its analogues exhibit similar shapes and sizes. It was possible to obtain a single‐crystal X‐ray structure of **[L1 + 2B1S]** (see Figure 4) and comparison of this structure to that of **[L1 + 2B1]** supports this hypothesis. Unfortunately, however, crystallisation experiments of **[L1 + 2B1N]** were unsuccessful. Thus, we investigated the size of the cages in solution by DOSY NMR experiments, which demonstrated the hydrodynamic radii are similar for all three structures (see Section S4). Further evidence was obtained from IMS‐MS experiments, which indicated that the drift times for the three cages are similar (see Section S5), supporting the conclusion that the subtle differences in the linker structures have little effect on the overall molecular size in these systems.^83^, ^84^

**Figure 5:**
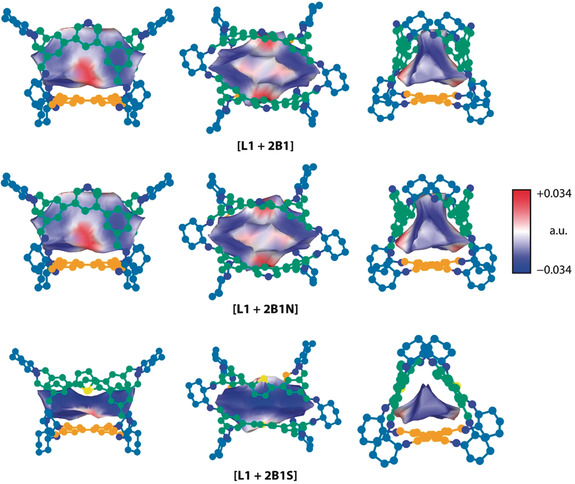
Side‐, window‐, and top‐views of the electrostatic potential inside the cavities of the socially sorted **[L1 + 2B1X]** cages, mapped onto the 0.0004 a.u. total electron density isosurface calculated at the M06‐2X/6–311+G(d,p) level of theory.

We performed analysis of the shapes and electronic structures of the internal cage cavities to probe the effect of using different aldehydes. Cage geometries were optimised as described previously. These structures were used to calculate the total electron density and the electrostatic potential at the M06‐2X/6–311+G(d,p) level of theory. We found the 0.0004 a.u. density isosurface and selected a subsurface approximating the internal cavity of each cage (see Section S2.5 for the algorithm and the implementation). Figure 5 shows the mapping of the electrostatic potential onto the cavity surface. The potential around the main window between the two non‐linear aldehydes is most affected by the neighbouring heteroatoms, while the entire cavity surface becomes narrower and elongated in the case of the more expanded thiophene linker in **[L1 + 2B1S]**. The heteroatoms themselves have little effect on the shape of the void, but do affect its electronic properties, providing a subtle yet important distinction that may have consequences for guest binding and selectivity.

## Conclusion

Four new tetraaldehydes, two linear (**L1**, **L4**) and two non‐linear (**B1**, **B2**), were synthesised and treated with *R*,*R*‐**CHDA** to form three new **Tet^3^Di^6^** organic cages: **[3L1]**, **[3L4]**, and **[3B2]**. Aldehyde **B1** did not form the expected cage **[3B1]** when treated with *R*,*R*‐**CHDA** or *S*,*S*‐**CHDA**. However, upon co‐crystallisation of reaction mixtures containing **B1** and both enantiomers of **CHDA**, the formation of the pseudo‐*C*_3*h*_‐symmetric cage **[3B1‐*RS*]** was detected. We exploited the lack of a stable homochiral cage **[3B1]** to form low‐symmetry cage compounds containing mixtures of **B1** and linear aldehydes **L1–L4** of varying lengths. Computationally obtained formation energies of the resultant cages were able to rationalise the experimentally observed resultant cages. In particular, the heteroleptic cage **[L1 + 2B1]** was predicted to be more stable than the corresponding homoleptic cages **[3L1]** and **[3B1]**, and was indeed preferentially formed. Two heteroatom‐containing analogues of **[L1 + 2B1]** were formed using this strategy, demonstrating the generality of the social self‐sorting approach to synthesis of organic cages of low symmetry. The slight change in the aldehyde geometry and the incorporation of heteroatoms did not affect the overall size of the cage molecules, while allowing for tuning of the shape and electronic properties of the internal cavity. We hope that these results will aid the design of more anisotropic organic cages for challenging separations and the selective encapsulation of biologically relevant low‐symmetry guests.

## Conflict of interest

The authors declare no conflict of interest.

## Supporting information

As a service to our authors and readers, this journal provides supporting information supplied by the authors. Such materials are peer reviewed and may be re‐organized for online delivery, but are not copy‐edited or typeset. Technical support issues arising from supporting information (other than missing files) should be addressed to the authors.

Supplementary
