# Supplementary material for: Inducing Social Self‐Sorting in Organic Cages To Tune The Shape of The Internal Cavity
Source: Angew Chem Int Ed Engl. 2020 Jul 16;59(38):16755–63. doi: 10.1002/anie.202007571 (PMC7540416; doi:10.1002/anie.202007571)
Supplement: Supplementary file 1 — Supplementary [file ANIE-59-16755-s001.pdf]

Angewandte Chemie
Eine Zeitschrift der Gesellschaft Deutscher Chemiker

Supporting Information

Inducing Social Self-Sorting in Organic Cages To Tune The Shape of The Internal Cavity**

*Valentina Abet[+], Filip T. Szczypiński[+], Marc A. Little, Valentina Santolini, Christopher D. Jones, Robert Evans, Craig Wilson, Xiaofeng Wu, Michael F. Thorne, Michael J. Bennison, Peng Cui, Andrew I. Cooper, Kim E. Jelfs, and Anna G. Slater**

anie_202007571_sm_miscellaneous_information.pdf

Electronic Supplementary Information

Contents

S1. Materials and methods 3
S1.1 Materials 3
S1.2 Thin layer chromatography (TLC) and column chromatography 3
S1.3 Analytical ultra-high-performance liquid chromatography (UPLC-MS) 3
S1.4 Nuclear magnetic resonance (NMR) spectroscopy 3
S1.5 Diffusion-ordered NMR spectroscopy (DOSY) 3
S1.6 Fourier-transform infrared spectroscopy (FTIR) 4
S1.7 Ion-mobility spectroscopy-mass spectrometry (IMS-MS) 4
S1.8 Single crystal X-ray diffraction (SC-XRD) 4
S2. Computational methods 5
S2.1 Conformer generation 5
S2.2 Density functional theory (DFT) methods 5
S2.3 Comparison with single crystal X-ray structures 7
S2.4 Modelling of the pseudo-C3h-symmetric RS cages 8
S2.5 Internal cage cavity identification analysis 9
S3. Synthetic procedures and characterisation 10
S3.1 Preparation of aldehyde precursors 10
Preparation of 5-(4,4,5,5-tetramethyl-1,3,2-dioxaborolan-2-yl)isophthalaldehyde 10
Preparation of L1 11
Preparation of L4 13

Preparation of B1 15
Preparation of B2 17
S3.2 Preparation of heteroatom-containing aldehyde precursors 19
Preparation of [B1S] 19
Preparation of [B1N] 21
S3.3 Preparation of single-aldehyde cages 23
Preparation of cage [3L1] 23
Preparation of cage [3B2] 26
Preparation of cage [3L4] 29
Attempt towards synthesis of cage [3B1] resulting in [3B1-RS] 32
S3.4 General procedure for screening for mixed aldehyde cages 33
Screening mixtures containing non-linear aldehyde B1 33
Screening mixtures containing non-linear aldehyde B2 38
S3.5 Preparation of mixed-aldehyde cages 42
Preparation of cage [L1 + 2B1] 42
Preparation of cage [L1 + 2B1S] 50
Preparation of cage [L1 + 2B1N] 53
S4. DOSY NMR spectra and analysis 55
S5. IMS-MS spectra and analysis 58
S6. SC-XRD refinement notes 59
S7. References 63

S1. Materials and methods

S1.1 Materials

All isomers of cyclohexane-1,2-diamine were purchased from TCI-UK and used as received. All other chemicals were purchased from Sigma Aldrich, Fluorochem, or Fisher and used as received. 5-Bromoisophthalaldehyde, 3,3'-dibromo-1,1'-biphenyl, 5,5'-(ethyne-1,2-diyl)diisophthalaldehyde, [1,1':4',1''-terphenyl]-3,3'',5,5''-tetracarbaldehyde and the porous organic cages **[3L2]** and **[3L3]** were synthesized according to literature procedures.[1–3]

S1.2 Thin layer chromatography (TLC) and column chromatography

Analytical TLC was carried out on Kieselgel F_{254} pre-coated silica gel plates (Merck). Visualisation was accomplished with UV light. Compounds were purified, where specified, by column chromatography or by using a Biotage Isolera 4 and flash-grade silica.

S1.3 Analytical ultra-high-performance liquid chromatography (UPLC-MS)

Analytical UPLC-MS analysis was conducted using an ACQUITY H-Class UPLC (Waters) with 254 nm UV detection, ACQUITY UPLC C18 1.7 µm 2.1 × 50 mm column (Waters, part number 186002350) and an ACQUITY tandem triple quadrupole mass spectrometer (TQD-MS, Waters). A flow rate of 600 µL min^{-1} of 100 % MeOH (MS grade) was used in all cases except for the reaction mixture of **L2-*d*$_4$** + **B1** + ***R,R*-CHDA**, which used a flow rate of 500 µL min^{-1}.

S1.4 Nuclear magnetic resonance (NMR) spectroscopy

Solution ^{1}H and ^{13}C NMR spectra were recorded using either a Bruker Avance 400 (400 MHz) or Bruker DRX500 (500 MHz) instrument. Chemical shifts are reported in ppm (*δ*) with reference to internal residual protonated species of the deuterated solvent used for ^{1}H and ^{13}C analysis. Spectra were taken at ambient probe temperature using TopSpin Version 3.6.2 (Bruker BioSpin, Billerica, MA, 2019).

S1.5 Diffusion-ordered NMR spectroscopy (DOSY)

Diffusion NMR data were acquired at 300 K using a bipolar pulse pair sequence (DSTEBPGP_3S in the Bruker library)[4] to remove any possible effects of convection from the diffusion measurements.[5,6] Sixteen magnetic field gradient amplitudes from 51.9 to 2.73 G cm^{-1} were used and incremented in equal steps of gradient. The diffusion delay time, Δ, was set to 0.10 s in order to obtain almost complete attenuation of cage signals for the range of gradients used. The gradient encoding time, δ, for all experiments was 1.3 ms and all gradients were half-sine in shape. For each gradient amplitude, 256 transients of 16384 complex data points were acquired for a total experimental time of ca. 3 h.

DOSY spectra and diffusion coefficients of the cages were processed and determined using the DOSY Toolbox software package.[7]

S1.6 Fourier-transform infrared spectroscopy (FTIR)

IR spectra were recorded using a Bruker Tensor 27 FT-IR spectrometer with Quest ATR (diamond crystal puck) attachment running Opus 6.5 software. Samples were analysed as dry powders for 16 scans with a resolution of 4 cm^{-1}. Spectra were recorded in transmission mode.

S1.7 Ion-mobility spectroscopy-mass spectrometry (IMS-MS)

Ion mobility spectroscopy mass spectrometry (IMS-MS) was performed using a Waters Synapt G2-Si Mass Spectrometer equipped with a DESI source and T-Wave ion mobility cell. A sample of the analyte (3.0 mg) was dissolved in dichloromethane (3.000 mL) with sonication. An aliquot of this solution (30 µL) was diluted in methanol (3.000 mL) and formic acid (0.1% v/v) and filtered before analysis. Data were collected over 16 accumulations with a capillary voltage of 3.0 kV, a bias voltage of 60 V, an infusion flow rate of 20 µL min^{-1}, and start and end IMS wave velocities of 1300 m s^{-1} and 150 m s^{-1} respectively.

S1.8 Single crystal X-ray diffraction (SC-XRD)

SC-XRD data sets were measured on a Rigaku MicroMax-007 HF rotating anode diffractometer (Mo-Kα radiation, λ = 0.71073 Å, Kappa 4-circle goniometer, Rigaku Saturn724+ detector); or at beamline I19, Diamond Light Source, Didcot, UK using silicon double crystal monochromated synchrotron radiation (λ = 0.6889 Å, Pilatus 2M detector). Absorption corrections, using the multi-scan method, were performed with the program SADABS.[8] For synchrotron X-ray data, collected at Diamond Light Source (λ = 0.6889Å), data reduction and absorption corrections were performed with xia2.[9] Structures were solved with SHELXT[10] and refined by full-matrix least squares on $|F|^2$ by SHELXL,[11] interfaced through the program OLEX2.[12] All non-H atoms were refined anisotropically and all H-atoms were fixed in geometrically estimated positions and refined using the riding model. Supplementary CIF files, which include structure factors, have been deposited at the Cambridge Crystal Data Centre under the submission codes: 2001201 for **[3L1]**; 2001206 **[3B1-*RS*]-*C*3*h***; 2001204 **[3B2]**; 2001208 **[L1+2B1]**-Hexane; 2001205 **[L1+2B1]**-Acetone; 2001203 **[L1+2B1]**-1,4-Dioxane; and 2001202 **[L1+2B1S]**.

S2. Computational methods

S2.1 Conformer generation

Initial structures were drawn manually in Maestro and high-temperature Molecular Dynamics (MD) simulations were run as an effective way to identify the lowest-energy conformations of the molecules.[13] The simulations were run with the MacroModel package, as part of the Schrödinger Suite (Schrödinger, LLC, New York, NY, 2018-4 release), in the NVE ensemble, with a time step of 1 fs and a total duration of 100 ns with the OPLS3e force field.[14] Structures were sampled every 10 ps and their geometry was optimised following the Polak–Ribière Conjugate Gradient algorithm with a derivative convergence criterion of 0.05 kJ mol^{-1} $Å^{-1}$. This procedure was carried out at both 500 and 1000 K to ensure sampling of the potential energy surface. When the simulations reached convergence, a set of conformers that lay within an energy window of 15 kJ mol^{-1} was analysed. Redundant conformers were removed with a maximum heavy atom deviation criterion of 0.5 Å and the resulting structures were optimised at the DFT level of theory.

S2.2 Density functional theory (DFT) methods

To get a more accurate energetic ranking of the conformations, the structures were optimised with the PBE functional,[15] triple-zeta molecularly optimised basis sets,[16] a GTH-type pseudopotential,[17–19] a plane wave[20] grid cut-off of 350 Ry and the Grimme-D3 dispersion correction[21,22] in CP2K.[23,24] Secondly, we performed single point calculations on the PBE-optimised structures using the M06-2X functional[25] and 6-311G(3df,3pd) basis set[26,27] in Gaussian16.[28] The M06-2X functional was successfully used for formation energy calculations of porous organic cages in the past[13] and does not require additional dispersion corrections, as these are already embedded in the functional. All DFT formation energies given in the main text are calculated as the differences of the total electronic energies from the single-point calculations at the M06-2X/6-311G(3df,3pd) level of theory. Example CP2K and Gaussian16 input files can be found in accompanying archive, together with final structures all precursors and cage molecules optimised following the above procedure. Resulting total electronic energies for all structures at the M06-2X/6-311G(3df,3pd) level are summarised in Table S1. If both open and closed cage conformations were identified, both corresponding energies can be found in Table S1.

Table S1 Total electronic energies of DFT-optimised precursors and cage compounds calculated at M06-2X/6-311G(3df,3pd)//PBE-GD3/TZVP-MOLOPT-GTH level of theory.

Precursors		Cages	
Compound	E / au	Compound	E / au
**L1**	-916.52971	**[3L1]**	-3911.775590
**L2**	-992.67376	**[3L2]**	-4140.224514
**L3**	-1147.56286	**[3L3]**	-4604.883778
**L4**	-1378.59542	**[3L4]**	-5298.000476
**B1**	-1147.56276	**[3B1] closed**	-4604.865204
**B2**	-1378.59562	**[3B1] open**	-4604.85956
R,R-**CHDA**	-346.53621	**[3B2] closed**	-5297.984579
H_2O	-76.42262	**[3B2] opens**	-5297.982897
**B1N**	-1163.60060	**[2L1 + B1]**	-4142.796778
**B1S**	-1468.33672	**[2L1 + B1N]**	-4158.81951
		**[2L1 + B1S]**	-4463.57075
		**[2L1 + B2]**	-4373.833036
		**[2L2 + B1]**	-4295.102286
		**[2L2 + B1] closed**	-4449.982846
		**[2L2 + B1] open**	-4449.981156
		**[2L2 + B2]**	-4526.137485
		**[2L3 + B1] closed**	-4604.867118
		**[2L3 + B1] open**	-4604.868363
		**[2L3 + B2] closed**	-4835.914469
		**[2L3 + B2] open**	-4835.908859
		**[2L4 + B1]**	-5066.943364
		**[2L4 + B2]**	-5297.989256
		**[2B1 + B2]**	-4835.912627
		**[B1 + 2B2]**	-5066.945583
		**[L1 + 2B1] closed**	-4373.840072
		**[L1 + 2B1] open**	-4373.84422
		**[L1 + 2B1N]**	-4405.92394
		**[L1 + 2B1S]**	-5015.39691
		**[L1 + 2B2]**	-4835.908228
		**[L2 + 2B2]**	-4912.065242
		**[L3 + 2B1] closed**	-4604.864903
		**[L3 + 2B1] open**	-4604.870122
		**[L3 + 2B2]**	-5066.950685
		**[L4 + 2B1]**	-4835.913802
		**[L4 + 2B2]**	-5297.990529
		**[L1 + 2B1N]**	-4405.92394
		**[2L1 + B1N]**	-4158.81951
		**[3B1N]**	-4652.98270
		**[L1 + 2B1S]**	-5015.39691
		**[2L1 + B1S]**	-4463.57075
		**[3B1S]**	-5567.20184

S2.3 Comparison with single crystal X-ray structures

Gas-phase conformations resulting from the treatment outlined above were compared against the experimental single crystal X-ray diffraction structures (see Figure S1). In order to account for the effects of computational solvent removal, the crystal structures were optimised following the Polak–Ribière Conjugate Gradient algorithm with a derivative convergence criterion of 0.05 kJ mol^{-1} $Å^{-1}$ with the OPLS3e force field in MacroModel (Schrödinger, LLC, New York, NY, 2018-4 release).

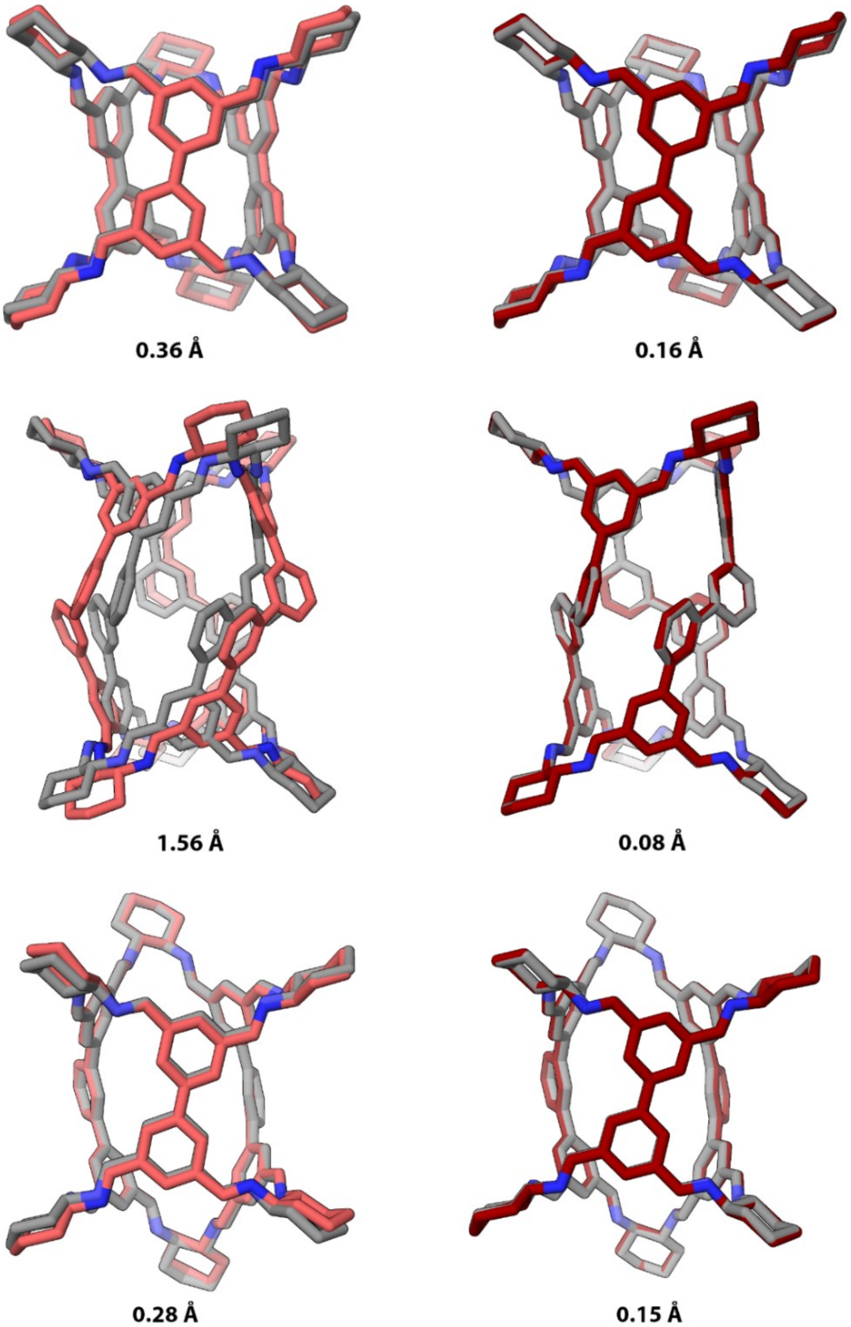

Figure S1 Overlays of the single crystal X-ray structures (left, pale pink), OPLS3e-minimised "computationally de-solvated" X-ray structures (right, dark red), and the computationally modelled

gas-phase structures (grey) of cages **[3L1]** (top), **[3L4]** (middle), and **[L1 + 2B1]** (bottom). Corresponding RMSDs are shown underneath. Hydrogen atoms were removed for clarity. Cages are not to scale in different overlays.

S2.4 Modelling of the pseudo-C_{3h}-symmetric *RS* cages

As the **[3B1]** cage was observed to re-equilibrate upon crystallisation into the pseudo-C_{3h}-symmetric stereoisomer, **[3B1-*RS*]**, which had not been computationally modelled, a set of new conformers for this cage was generated. The modified protocol was used, where we only employed a single high-temperature MD simulation at 1000 K. The gas-phase conformation thus obtained was compared against the experimental single crystal X-ray diffraction structures (see Figure S2).

Comparison of the electronic energies of the *RR* and *RS* cages can be found in Table S2 and shows that no other pseudo-C_{3h}-symmetric *RS* cages are expected for single aldehyde systems.

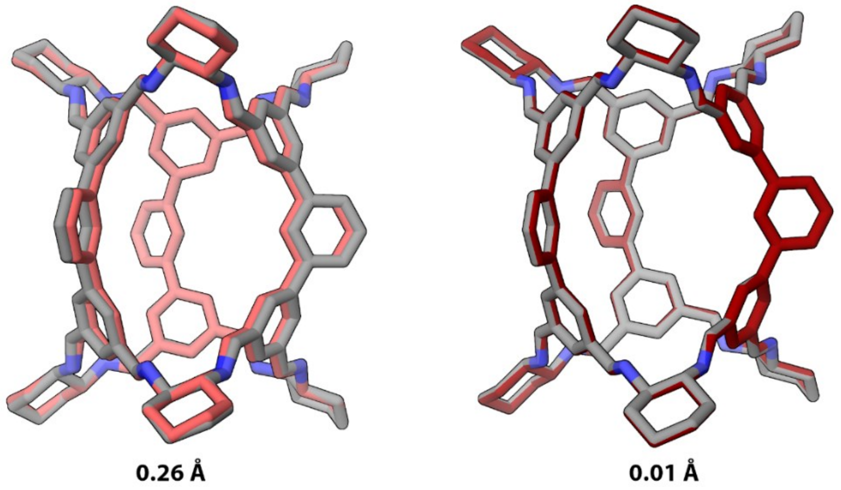

Figure S2 Overlays of the single crystal X-ray structure (left, pale pink), OPLS3e-minimised X-ray structure (right, dark red), and the computationally modelled gas-phase structure the **[3B1-*RS*]** cage (grey). Corresponding root-mean-square deviations in atomic positions are shown underneath. Hydrogen atoms were removed for clarity.

Table S2 Total electronic energies of DFT-optimised cage compounds containing only *R,R*-**CHDA** (***RR*** cages) and pseudo-C3h-symmetric cages containing mixtures of *R,R*- and *S,S*-CHDA (**RS** cages) calculated at M06-2X/6-311G(3df,3pd)//PBE-GD3/TZVP-MOLOPT-GTH level of theory.

**Structure**	***RR* Energy / au**	***RS* Energy / au**	***RS* - *RR* Energy / kJ mol^{-1}**
**[3L1]**	-3911.775590	-3911.742027	88
**[3L2]**	-4140.224514	-4140.216618	21
**[3L3]**	-4604.883778	-4604.873978	26
**[3L4]**	-5298.000476	-5297.983836	44
**[3B1] closed**	-4604.865204	-4604.873054	-21
**[3B1] open**	-4604.859560	-4604.872408	-34
**[3B2] closed**	-5297.984579	-5297.983678	2
**[3B2] open**	-5297.982897	-5297.983678	-2

S2.5 Internal cage cavity identification analysis

In order to analyse the shapes and the electronic structures of the cage cavities, we generated electron density and electrostatic potential CUBE files using the cubegen package at Medium grid spacing.[28] We then analysed those CUBE files using a purpose written Python package using modules from the SciPy[29] and NumPy[30] packages. Our approach to qualify which density isosurface points, calculated at the (0.0004 ± 1%) a.u total electron density isovalue, comprised the cage cavity was to confirm they were located closer to the geometric centre of mass of the cage than their five nearest neighbouring atoms. Although crude, this approach successfully identified the non-convex cavities of the analysed cages upon visual inspection. Subsequently, electrostatic potential values were mapped on thus identified internal cavity surfaces and the results were visualised using the Ovito package.[31]

Example codes can be found in the accompanying archive and the purpose-written Python package can be found at https://github.com/fiszczyp/void_surface.

S3. Synthetic procedures and characterisation

S3.1 Preparation of aldehyde precursors

Preparation of 5-(4,4,5,5-tetramethyl-1,3,2-dioxaborolan-2-yl)isophthalaldehyde

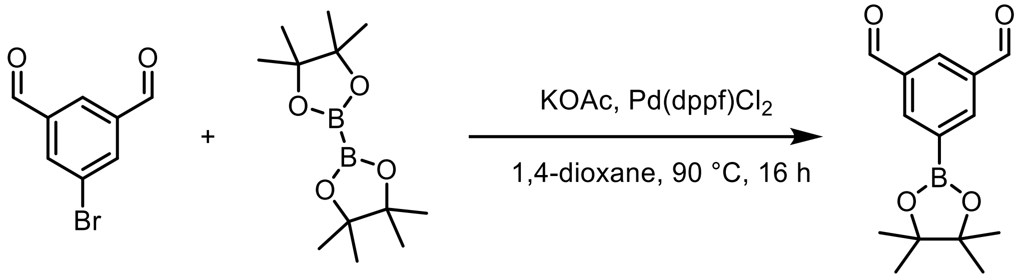

To a solution of 5-bromoisophthalaldehyde (15.0 g, 70.4 mmol, 1.0 eq.), bis(pinacolato)diboron (19.7 g, 77.4 mmol, 1.1 eq.) and KOAc (20.7 g, 211.2 mmol, 3.0 eq.) in anhydrous 1,4-dioxane (100 mL), Pd(dppf)Cl_2 (1.54 g, 2.11 mmol, 0.03 eq.) was added. The reaction was heated to 90 °C and stirred for 16h. The solid precipitate was filtered off and washed with Et_2O. The filtrate was washed with water (100 mL) and brine (100 mL), dried over $MgSO_4$ and evaporated to dryness. The residue was dissolved in DCM and purified by flash chromatography on silica gel, eluting with a 0 to 50% EtOAc in DCM gradient. Likely fractions were evaporated *in vacuo*, affording the boronic ester as an oil that solidifies on standing (15.9 g, 61.2 mmol, 87%). ^{1}H NMR (500 MHZ, $CDCl_3$): 10.11 (s, 2H), 8.54 (d, 2H, *J* = 1.7 Hz), 8.44 (t, 1H, *J* = 1.7 Hz), 1.36 (s, 12H). HRMS-CI: calcd. for $C_{14}H_{18}BO_4$ $[M+1]^+$ 260.1329, found 260.1319.

The spectra agree with literature protocols.[32]

Preparation of L1

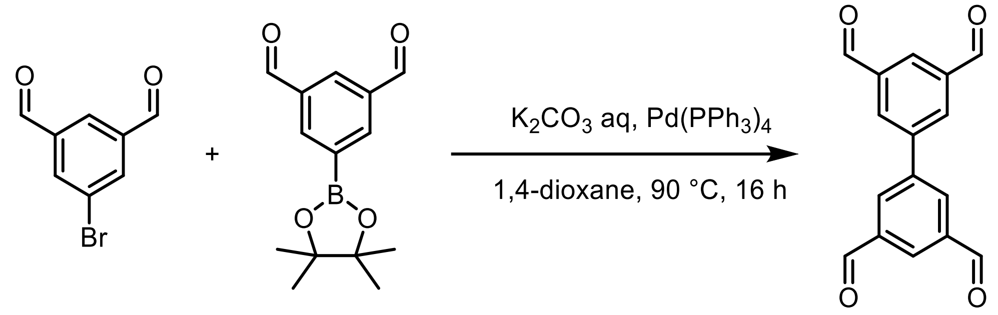

To a solution of 5-bromoisophthalaldehyde (300.0 mg, 1.4 mmol, 1.0 eq.) and 5-(4,4,5,5-tetramethyl-1,3,2-dioxaborolan-2-yl)isophthalaldehyde (403.3 mg, 1.5 mmol, 1.1 eq.) in anhydrous 1,4-dioxane (10 mL) 1M K_2CO_3 aqueous solution (1.7 mL, 1.2 eq.) was added. The flask was back filled with nitrogen, then $Pd(PPh_3)_4$ (81.1 mg, 0.07 mmol, 0.05 eq.) was added. The reaction was heated to 90 °C and stirred overnight. The off-white solid that crashed out was collected by filtration, washed with EtOAc and used without any further purification (253 mg, 0.95 mmol, 68%). ^{1}H NMR (500 MHz, DMSO-d_6): 10.23 (s, 4H), 8.37 (d, 4H, J = 1.4 Hz), 8.48 (t, 2H, J = 1.4 Hz). ^{13}C NMR (125 MHz, DMSO-d_6): 192.7, 139.7, 137.8, 133.4, 129.3. FT-IR (υ, cm^{-1}): 1699, 1652, 1558, 1457, 668. MALDI-TOF: calcd. for $C_{16}H_{10}O_4$ [M] 266.058, found 266.063.

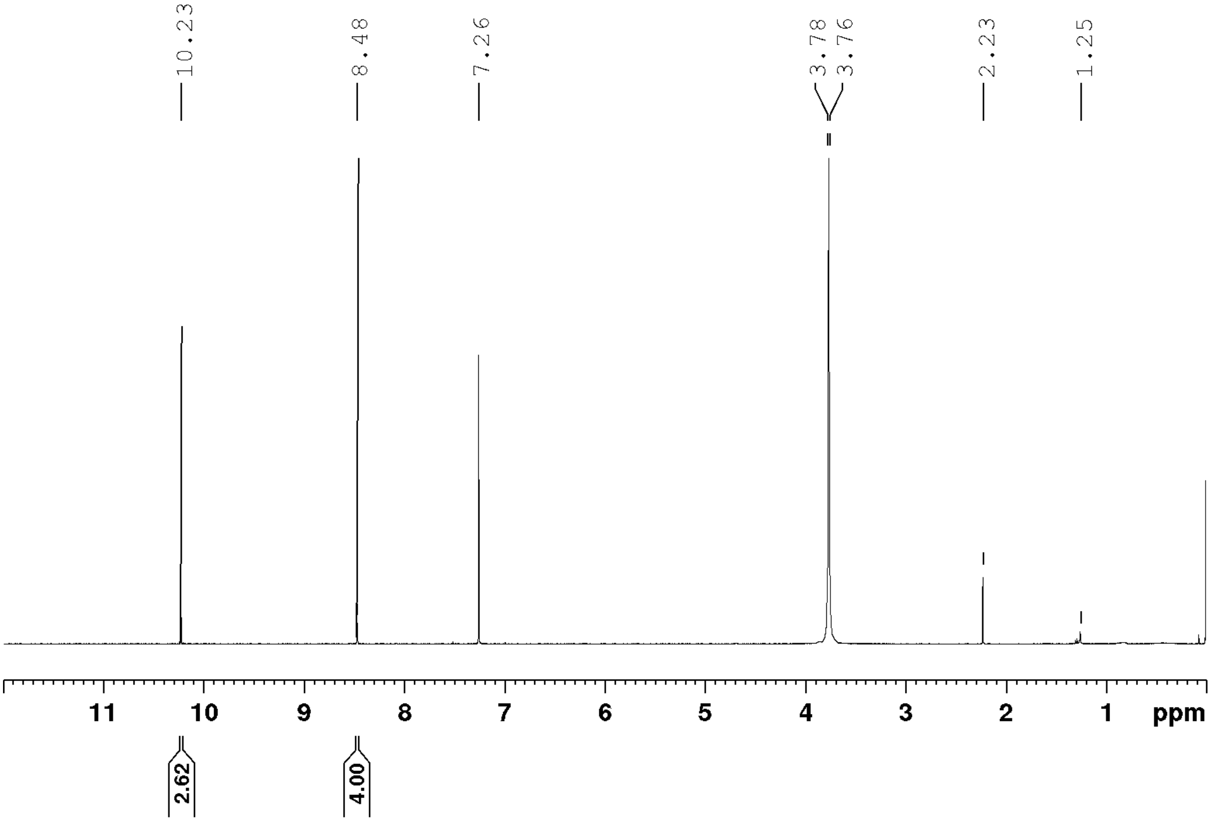

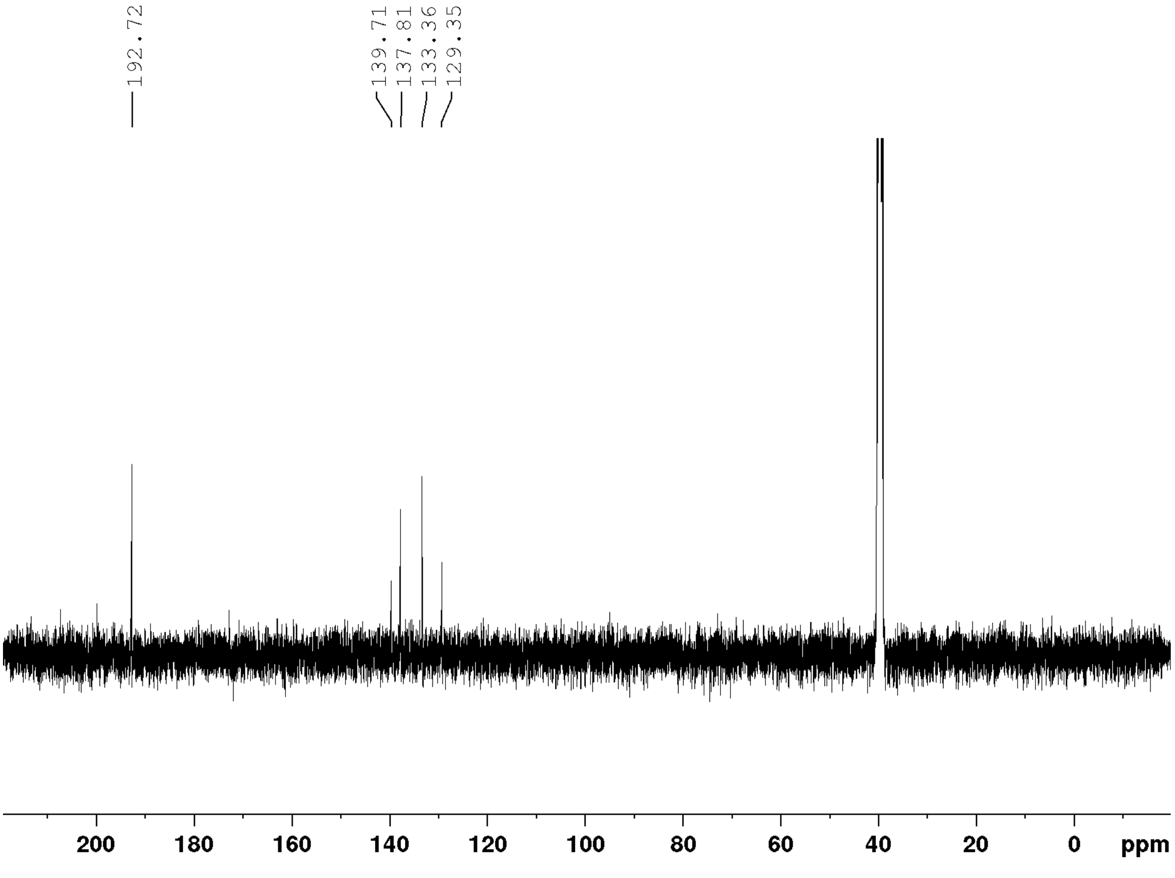

Figure S3 ^{1}H ($CDCl_3$, upper) and ^{13}C (DMSO-d_6, lower) NMR of **L1**.

Preparation of L4

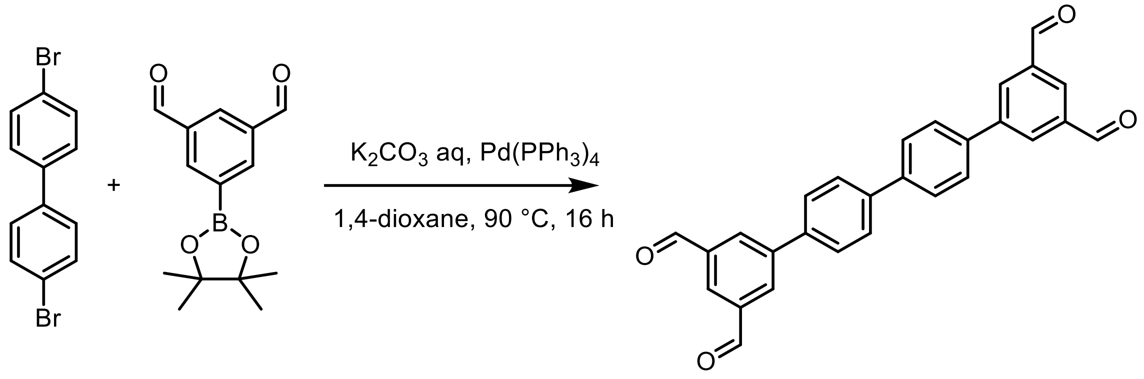

To a solution of 4,4'-dibromo-1,1'-biphenyl (1 g, 3.2 mmol, 1.0 eq.) and 5-(4,4,5,5-tetramethyl-1,3,2-dioxaborolan-2-yl)isophthalaldehyde (2.1 g, 8.0 mmol, 2.5 eq.) in anhydrous 1,4-dioxane (200 mL), 2M K_2CO_3 aqueous solution (12 mL, 1.2 eq.) was added. The flask was back filled with nitrogen, then $Pd(PPh_3)_4$ (185 mg, 0.16 mmol, 0.05 eq.) was added. The reaction was heated to 90 °C and stirred overnight. The off-white solid that crashed out was collected by filtration, washed with EtOAc and used without any further purification (561 mg, 1.34 mmol, 42 %). ^{1}H NMR (400 MHz, DMSO-*d₆*): 10.23 (s, 4H), 8.61 (d, 4H, J = 1.4 Hz), 8.41(t, 4H, *J* = 1.4 Hz), 8.02 – 7.97 (m, 4H). ^{13}C NMR (125 MHz, DMSO-*d₆*): 193.21, 141.71, 139.85, 137.99, 137.51, 133.36, 128.49, 128.08, 127.97. FT-IR (υ, cm^{-1}): 1692, 1596, 1505, 1459, 1392, 1378, 1318, 1248, 1202, 1140, 1005, 971, 885, 846, 809. 752, 736, 683, 648, 579, 544, 516, 475, 444. MALDI-TOF: calcd. for $C_{16}H_{10}O_4$ [M] 418.121, found 417.907

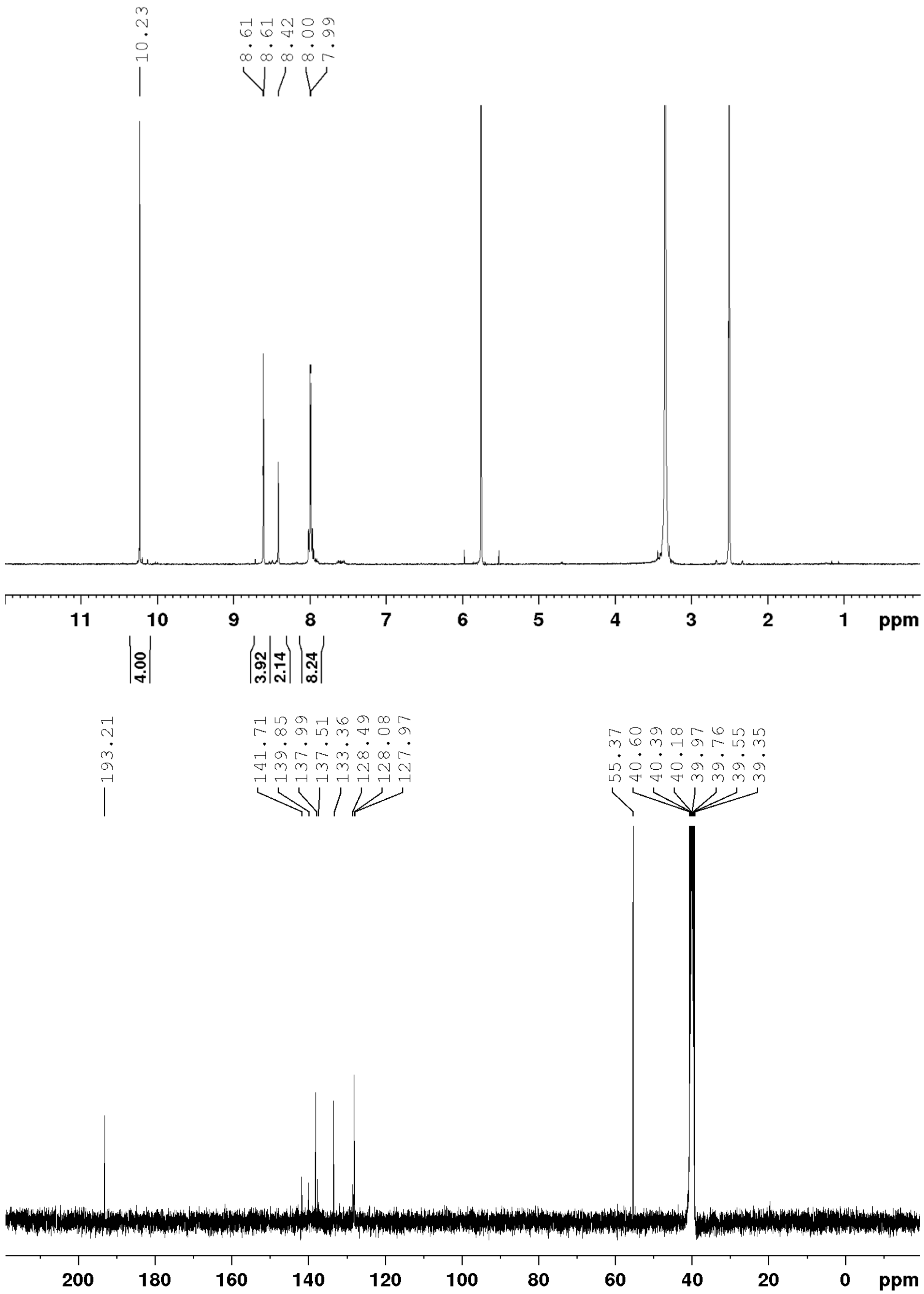

Figure S4 ^{1}H (DMSO-d_6, upper) and ^{13}C (DMSO-d_6, lower) NMR of **L4**.

Preparation of B1

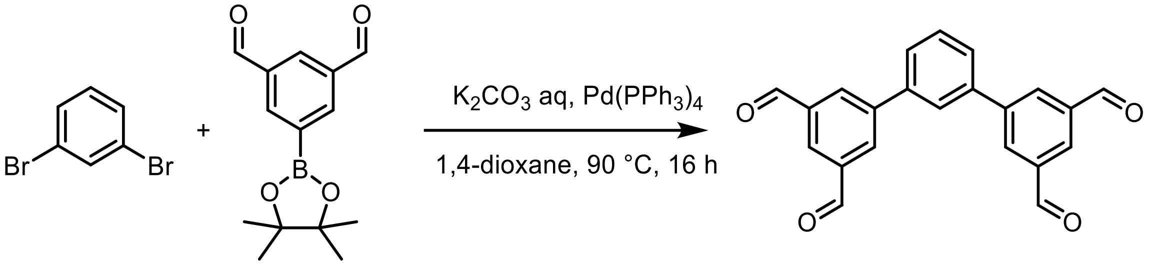

Following the general procedure, using 1,3-dibromobenzene (100.0 mg, 0.42 mmol), 5-(4,4,5,5-tetramethyl-1,3,2-dioxaborolan-2-yl)isophthalaldehyde (264.5 mg, 1.01 mmol), 1M K_2CO_3 aqueous solution (1 mL) in 1,4-dioxane (5 mL), an off-white solid crashed out. This was collected by filtration, washed with EtOAc and used without any further purification (119 mg, 0.35 mmol, 83%). ^{1}H NMR (500 MHZ, DMSO-*d*$_6$): 10.22 (s, 4H), 8.65 (d, 4H, *J* = 1.4 Hz), 8.43 (t, 2H, *J* = 1.4 Hz), 8.27 (t, 1H, *J* = 1.7 Hz), 7.94 (dd, 2H, *J* = 1.7 and 7.7 Hz), 7.73 (t, 1H, *J* = 7.7 Hz). ^{13}C NMR (125 MHz, DMSO-*d*$_6$): 192.8, 141.7, 139.0, 137.6, 133.5, 130.3, 128.3, 127.4, 125.9. FT-IR (υ, cm^{-1}): 1751, 1361, 1315, 1209, 653. MALDI-TOF: calcd. for $C_{22}H_{14}O_4$ [M] 342.099, found 342.088.

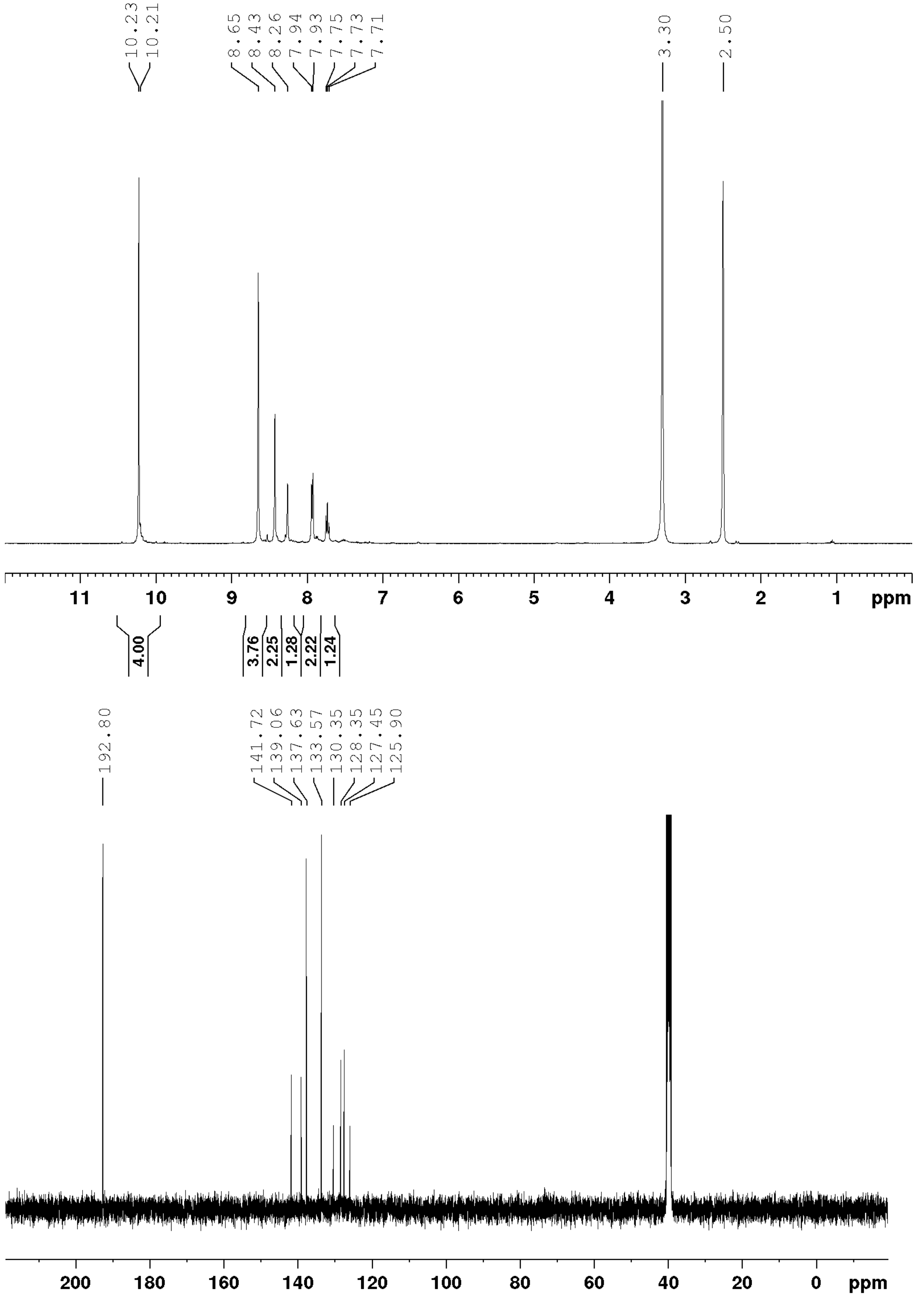

Figure S5 ^{1}H (DMSO-d_6, upper) and ^{13}C (DMSO-d_6, lower) NMR of **B1**.

Preparation of B2

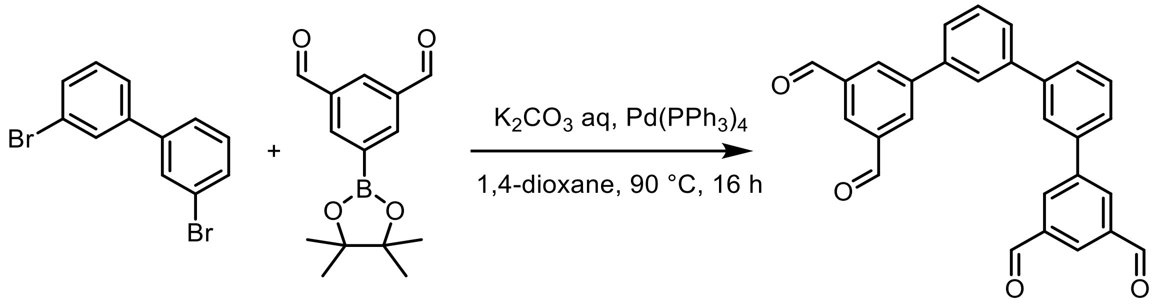

To a solution of 3,3'-dibromo-1,1'-biphenyl[2] (500.0 mg, 1.6 mmol, 1.0 eq.) and 5-(4,4,5,5-tetramethyl-1,3,2-dioxaborolan-2-yl)isophthalaldehyde (1.04 g, 4.0 mmol, 2.5 eq.) in anhydrous 1,4-dioxane (10 mL) 1M K_2CO_3 aqueous solution (4 mL, 2.5 eq.) was added. The flask was back filled with nitrogen, then $Pd(PPh_3)_4$ (92.4 mg, 0.08 mmol, 0.05 eq.) was added. The reaction was heated to 90 °C and stirred overnight. The off-white solid that crashed out was collected by filtration, washed with EtOAc and used without any further purification (400 mg, 0.96 mmol, 60%). ^{1}H NMR (500 MHz, DMSO-d_6): 10.21 (s, 4H), 8.64 (d, 4H, J = 1.4 Hz), 8.40 (t, 2H, J = 1.4 Hz), 8.20 (t, 2H, J = 1.7 Hz), 7.89 (m, 4H), 7.69 (t, 2H, J = 1.7 Hz). ^{13}C NMR (125 MHz, DMSO-d_6): 192.9, 142.0, 141.1, 138.8, 137.6, 133.7, 130.1, 127.9, 127.6, 126.6, 125.9. FT-IR (υ, cm^{-1}): 1703, 1692, 1597, 1399, 1139, 1007, 873, 700. MALDI-TOF: calcd. for $C_{28}H_{18}O_4$ [M] 418.45, found 418.61.

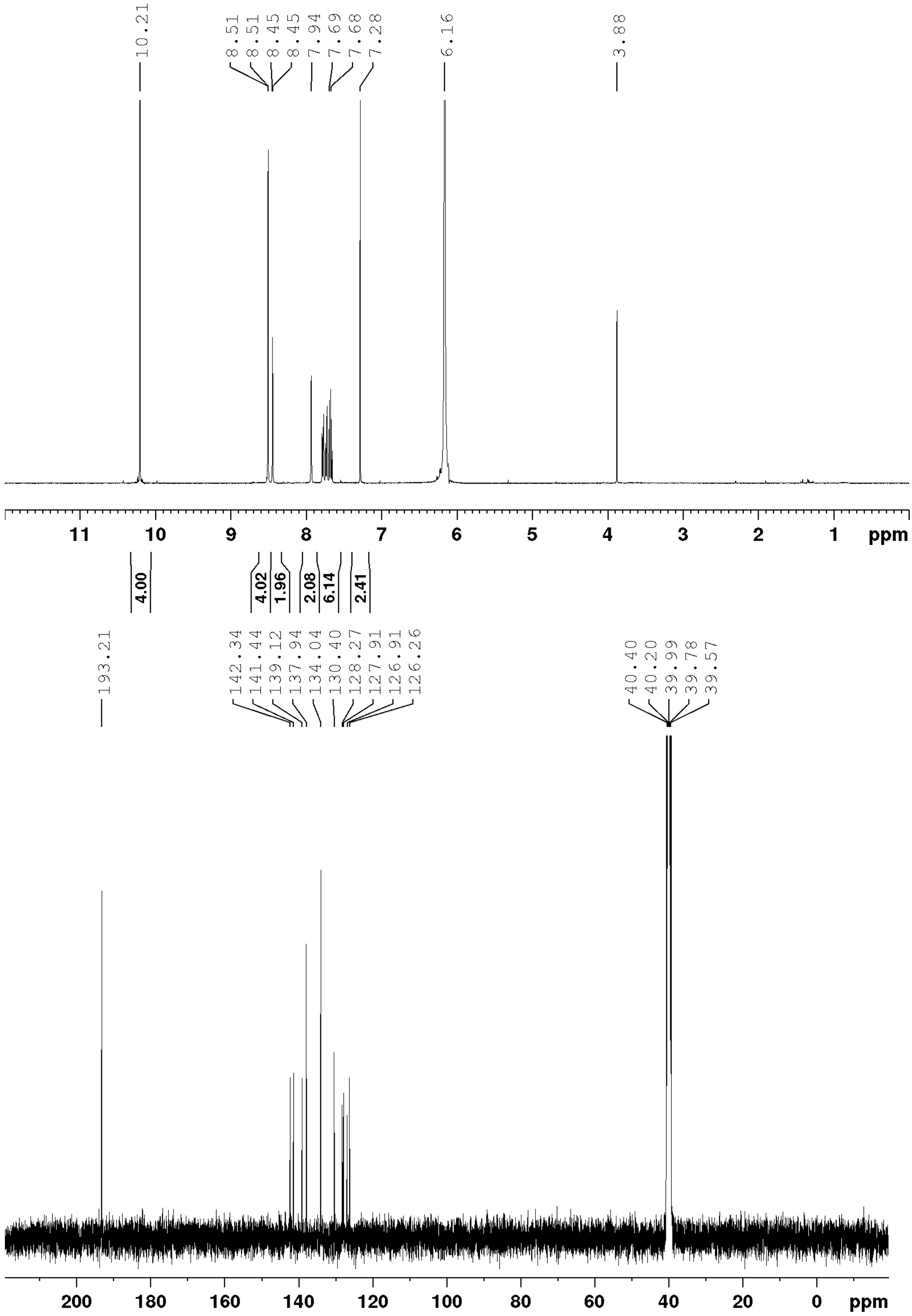

Figure S6 ^{1}H ($CDCl_3$, upper) and ^{13}C (DMSO-d_6, lower) NMR of **B2**.

S3.2 Preparation of heteroatom-containing aldehyde precursors

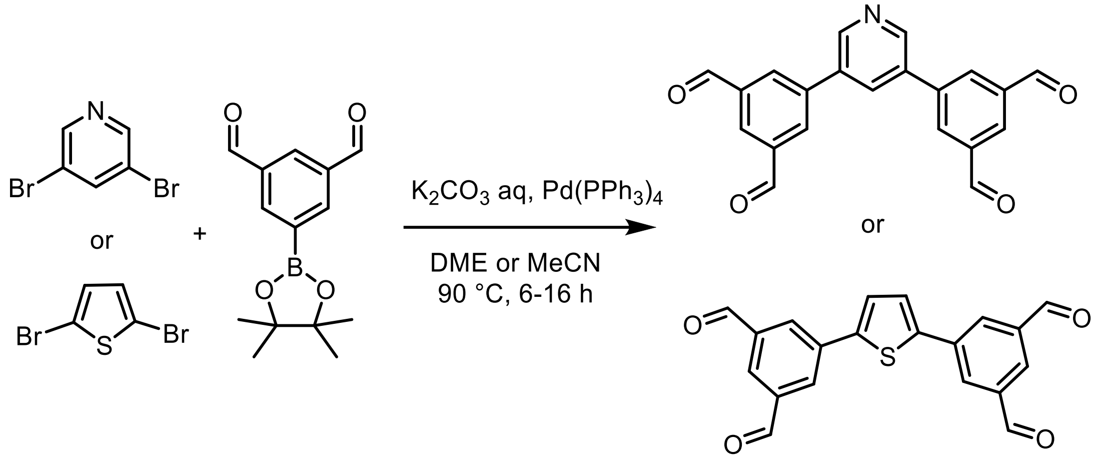

To a solution of the dibromo(hetero)aryl (1.0 eq.) and 5-(4,4,5,5-tetramethyl-1,3,2-dioxaborolan-2-yl)-isophthalaldehyde (2.4 eq.) in anhydrous DME (12.5 mL) 1M K_2CO_3 aqueous solution (2.5 eq.) was added. The flask was back filled with nitrogen and deoxygenated, then $Pd(PPh_3)_4$ (0.05 eq.) was added. The reaction was heated to 90^oC and stirred for 6h. The solid that crashed out was collected by filtration, washed with EtOAc and used without any further purification.

Preparation of [B1S]

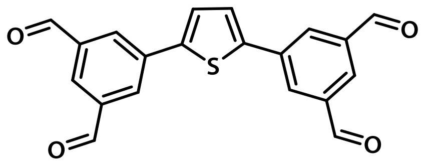

Following the general procedure, using 2,5-dibromothiophene (100.0 mg, 0.41 mmol), 5-(4,4,5,5-tetramethyl-1,3,2-dioxaborolan-2-yl)isophthalaldehyde (258.1 mg, 0.99 mmol), 1M K_2CO_3 aqueous solution (1 mL) in DME (5 mL), a yellow solid crashed out. This was collected by filtration, washed with EtOAc and used without any further purification (141.4 mg, 0.40 mmol, 99%). ^{1}H NMR (500 MHz, DMSO-*d*$_6$): 10.17 (s, 4H), 8.54 (d, 4H, *J* = 1.2 Hz), 8.34 (t, 2H, *J* = 1.2 and 2.5 Hz), 7.92 (s, 2H). ^{13}C NMR (125 MHz, DMSO-*d*$_6$): 192.5, 141.7, 137.8, 135.1, 131.1, 128.5, 127.5. FT-IR (υ, cm^{-1}): 1739, 1367, 1225, 1216, 668. MALDI-TOF: calcd. for $C_{20}H_{11}O_4S$ [M-H]$^-$ 347.038, found 347.132.

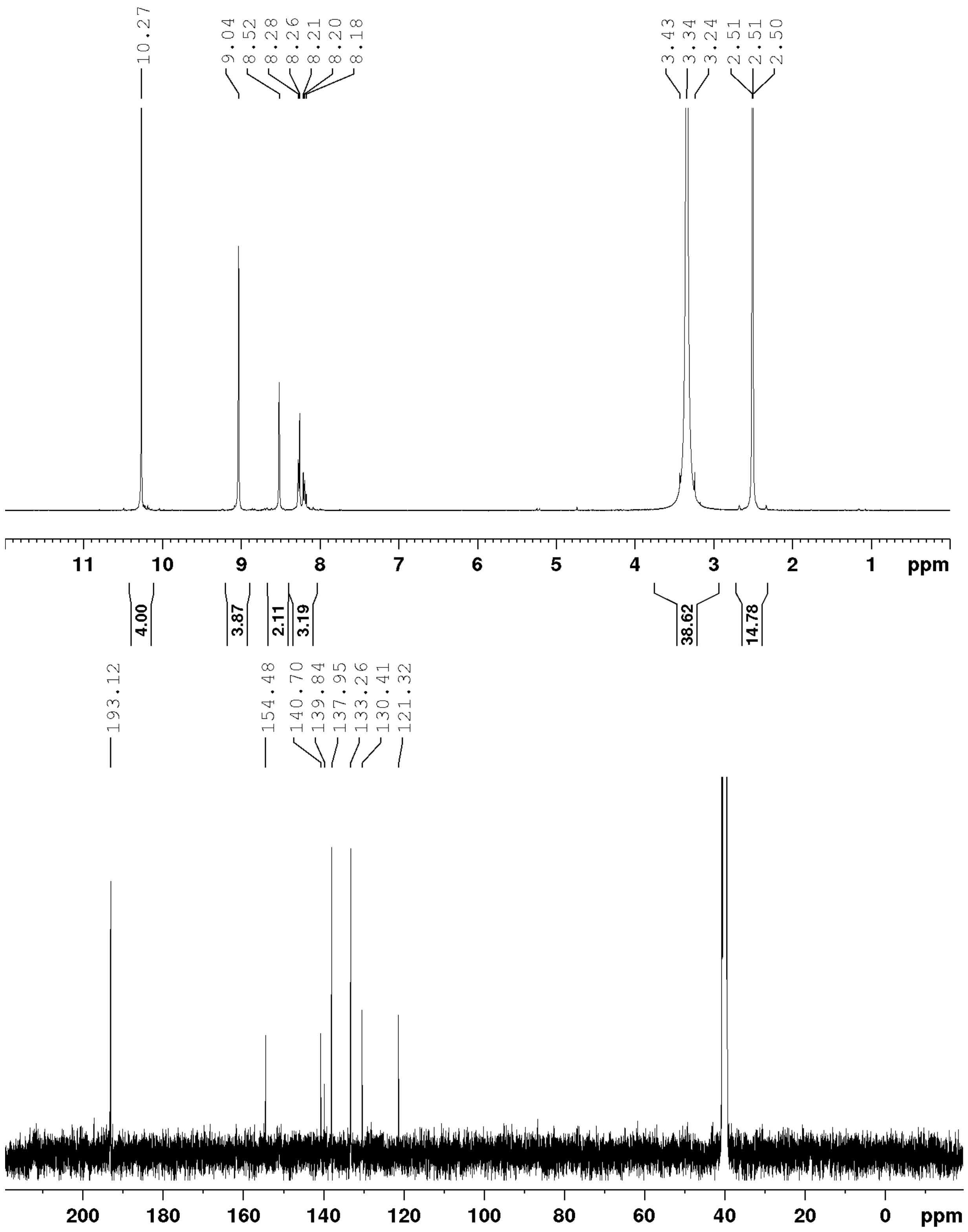

Figure S7 ^{1}H (DMSO-d_6, upper) and ^{13}C (DMSO-d_6, lower) NMR of **B1S**.

Preparation of [B1N]

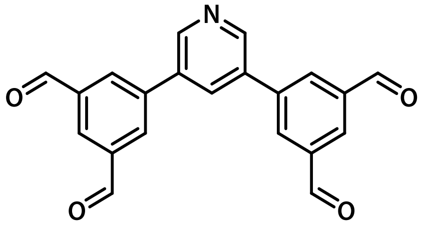

3,5-Dibromopyridine (237.0 mg, 1.0 mmol, 1.0 eq.), K_2CO_3 (552.4 mg, 4.0 mmmol, 4.0 eq.) and 5-(4,4,5,5-tetramethyl-1,3,2-dioxaborolan-2-yl)isophthalaldehyde (520.3 mg, 2.0 mmol, 2.0 eq.) were dissolved in anhydrous MeCN (10 mL) and water (10 mL). The flask was back filled with argon, then $Pd(PPh_3)_4$ (115.6 mg, 0.1 mmol, 0.1 eq.) was added. The reaction was heated to 90°C and stirred for 16h. The off-white solid that crashed out was collected by filtration, washed with EtOAc and used without any further purification (180.4 mg, 0.52 mmol, 52%). ^{1}H NMR (500 MHz, DMSO-*d6*): 10.23 (s, 4H), 9.14 (d, 1H, *J* = 1.4 Hz), 8.75-8.72 (m, 4H), 8.48 (t, 2H, *J* = 1.4 and 2.8 Hz). ^{13}C NMR (125 MHz, DMSO-*d6*): 192.3, 147.6, 138.4, 137.4, 133.6, 133.5, 133.0, 128.7. FT-IR (υ, cm^{-1}): 1733, 1359, 1222, 659. MALDI-TOF: calcd. for $C_{21}H_{12}NO_4$ [M-H]$^-$ 342.078, found 342.159.

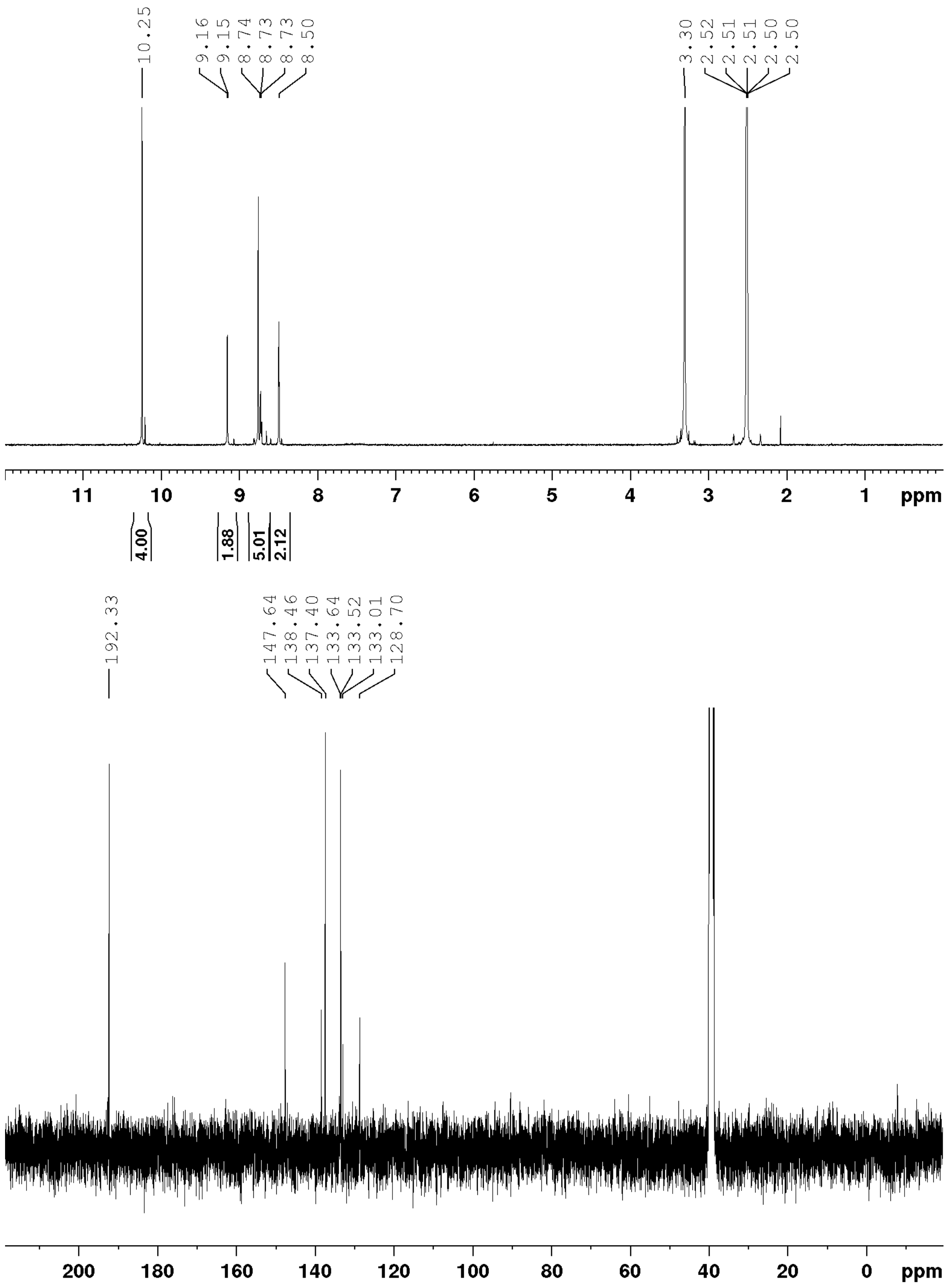

Figure S8 ^{1}H (DMSO-d$_6$, upper) and ^{13}C (DMSO-d$_6$, lower) NMR of **B1N**.

S3.3 Preparation of single-aldehyde cages

Preparation of cage [3L1]

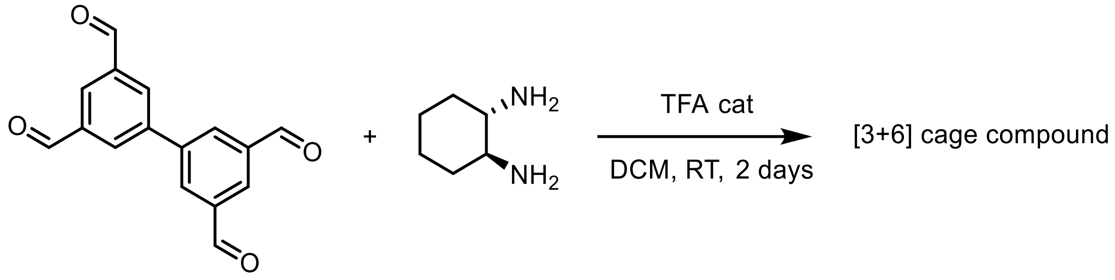

To a stirred solution of [1,1'-biphenyl]-3,3',5,5'-tetracarbaldehyde (60 mg, 0.23 mmol, 1 eq.) and (1*R*,2*R*)- or (1*S*,2*S*)-cyclohexane-1,2-diamine (51 mg, 0.45 mmol, 2 eq.) in DCM (30 mL), 2-3 drops of TFA were added. The reaction was stirred at RT for 2d. The mixture was then diluted with DCM to a 2 mg/mL concentration and the solids were filtered off. The filtrate was concentrated *in vacuo* to ~15 mL, hexane (~30 mL) was added with stirring and the resulting precipitate was collected by filtration, affording an off-white solid (43 mg, 45 %). ^{1}H NMR (400 MHz, $CDCl_3$): 8.31 (s, 6H), 8.27 (s, 6H), 8.12 (s, 6H), 7.81 (s, 6H), 7.68 (s, 6H), 3.54-3.48 (m, 6H), 3.26-3.20 (m, 6H), 2.03-1.53 (m, 48 H). ^{13}C NMR (125 MHz, $CDCl_3$): 160.93, 160.25, 153.70, 136.75, 128.91, 126.45, 123.09, 75.18, 73.59, 32.08, 24.50, 24.31, 24.10. FT-IR (ʋ, cm^{-1}): 2926, 2855, 1643, 1593, 1446, 1372, 1342, 1309, 1265, 1163, 1139, 1084, 1039, 975, 934, 874, 823, 796, 696, 662, 634, 564, 539, 513, 471, 440, 413. MALDI-TOF: calcd. for $C_{84}H_{91}N_{12}$ $[M+H]^+$ 1267.7490, found 1268.6

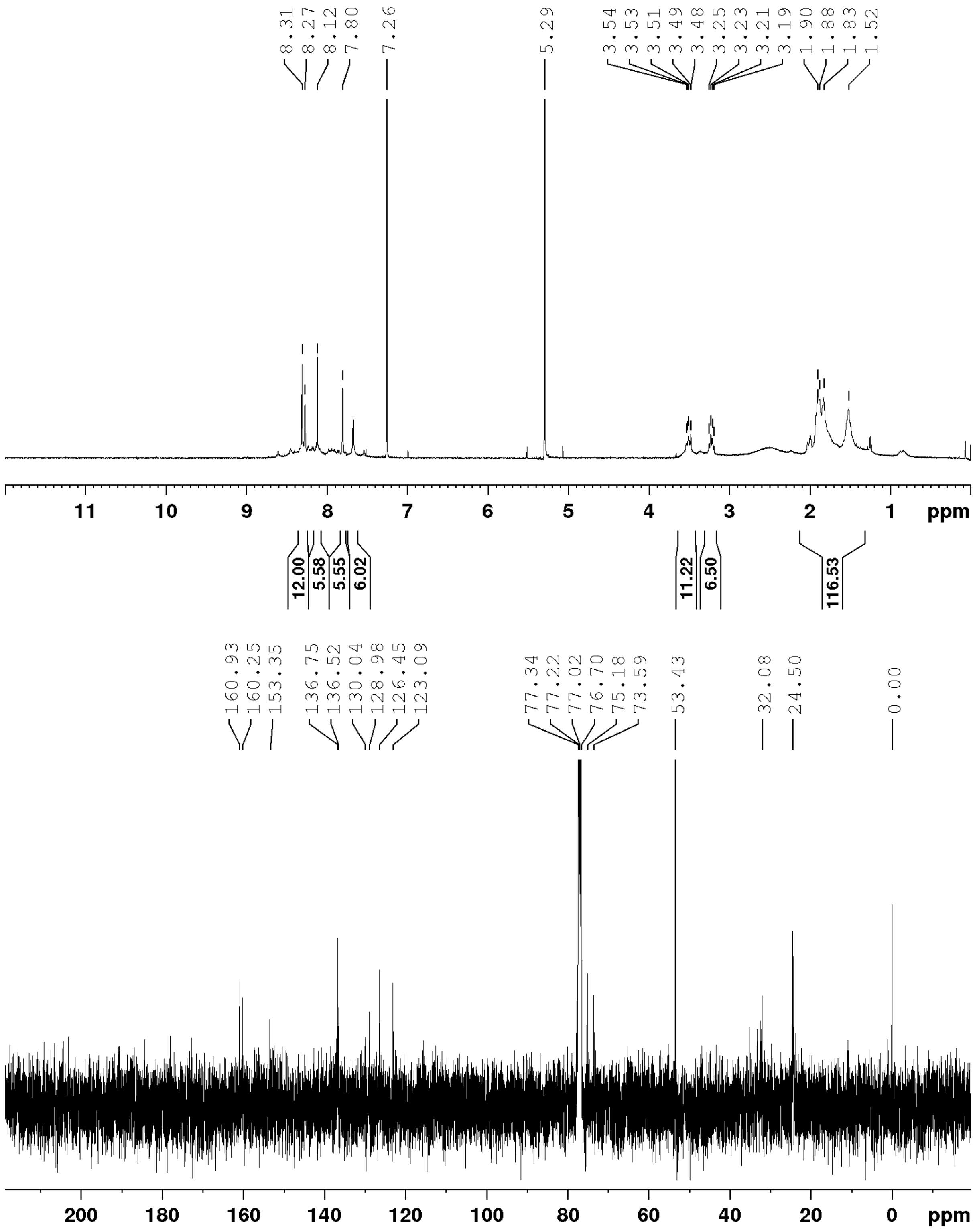

Figure S9 ^{1}H ($CDCl_3$, upper) and ^{13}C ($CDCl_3$, lower) NMR of cage **[3L1]**.

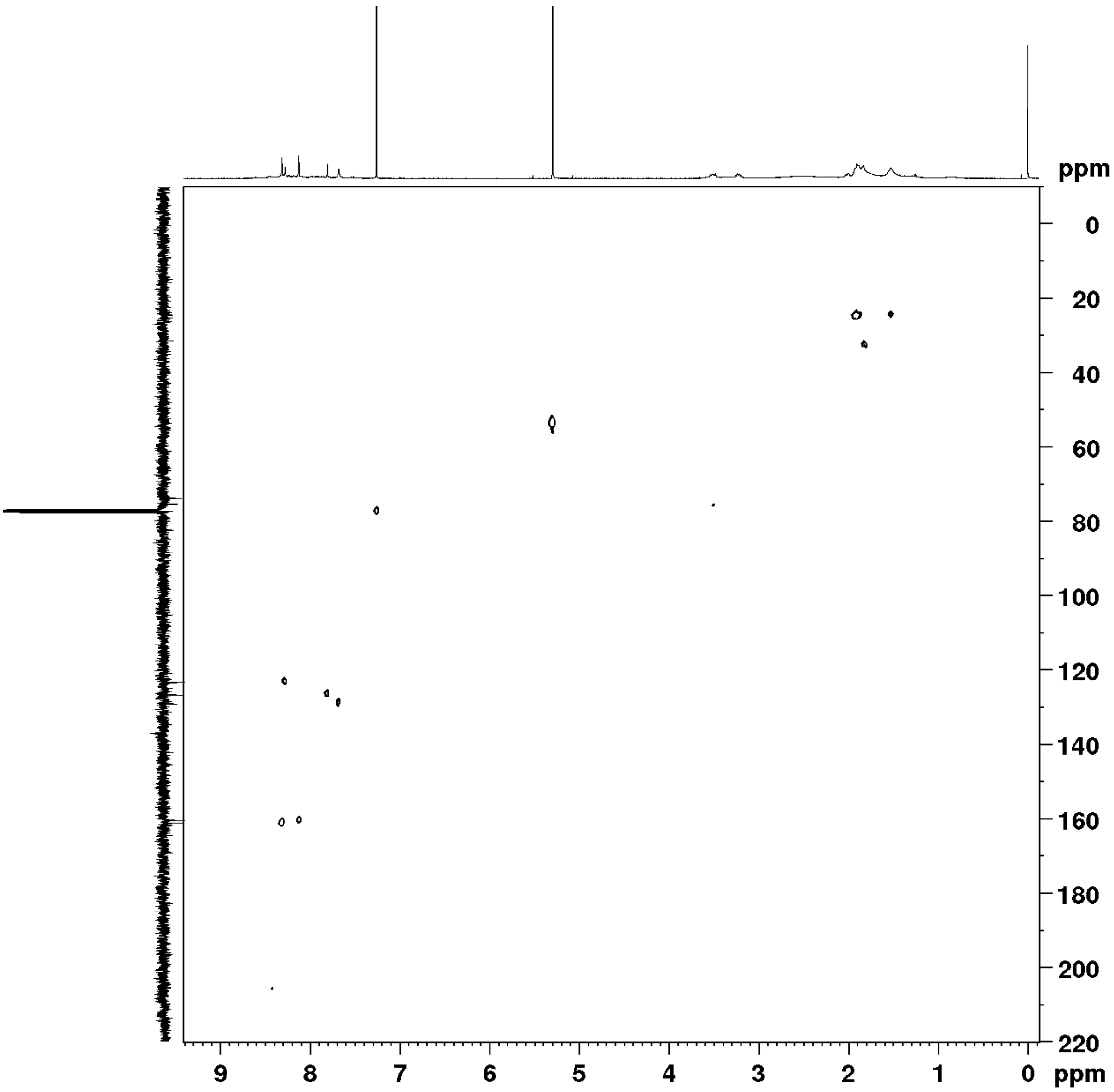

Figure S10 ^{1}H-^{13}C HSQC NMR ($CDCl_3$) of cage **[3L1]**.

Preparation of cage [3B2]

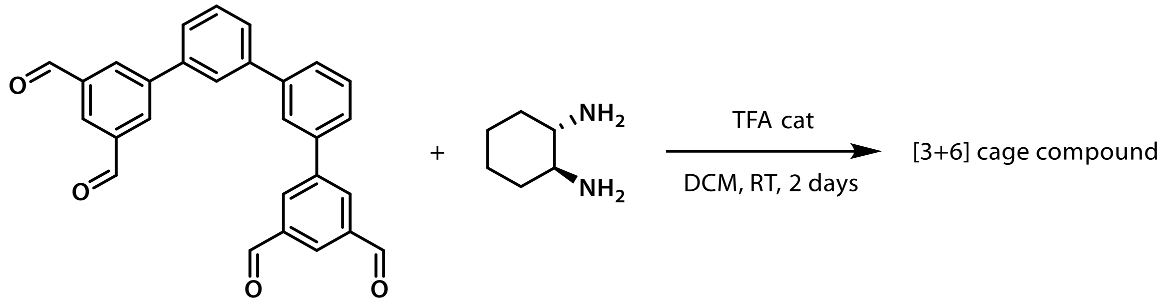

To a stirred solution of [1,1':3',1'':3'',1'''-quaterphenyl]-3,3''',5,5'''-tetracarbaldehyde (200 mg, 0.48 mmol, 1 eq.) and (1*R*,2*R*)- or (1*S*,2*S*)-cyclohexane-1,2-diamine (109 mg, 0.96 mmol, 2 eq.) in DCM (10 mL), 2-3 drops of TFA were added. The reaction was stirred at RT for 2d. The mixture was then diluted with DCM to a 2 mg/mL concentration and the solids were filtered off. The filtrate was concentrated *in vacuo* to ~20 mL, hexane (~40 mL) was added with stirring and the resulting precipitate was collected by filtration, affording an off-white solid (83.6 mg, 54% for the *R,R* cage, 87.5 mg, 56% for the *S,S* cage).

R,R cage: ^{1}H NMR (500 MHz, $CDCl_3$): 8.26 (d, 12H, *J* = 3.2 Hz), 8.20 (s, 6H), 7.92 (s, 6H), 7.59 (s, 6H), 7.56-7.53 (m, 12H), 7.47-7.45 (m, 6H), 7.32 (t, 6H, *J* = 7.5 Hz), 3.49-3.37 (m, 12H), 1.87-1.51 (m, 48H). FT-IR (υ, cm^{-1}): 2929, 2857, 1646, 1595, 1447, 1201, 1092, 878, 789, 688. HRMS (ESI^{+}): *m/z* calcd. for $C_{120}H_{115}N_{12}$ $[M+H]^{+}$ 1723.9367, found 1723.9293.

S,S cage: ^{1}H NMR (500 MHz, $CDCl_3$): 8.26 (d, 12H, *J* = 3.2 Hz), 8.19 (s, 6H), 7.92 (s, 6H), 7.59 (s, 6H), 7.53-7.53 (m, 12H), 7.46-7.44 (m, 6H), 7.34 (t, 6H, *J* = 7.5 Hz), 3.49-3.37 (m, 12H), 1.98-1.71 (m, 48H). FT-IR (υ, cm^{-1}): 1668, 1646, 1595, 1448, 1371, 1200, 1132, 877, 701. HRMS (ESI^{+}): *m/z* calcd. for $C_{120}H_{115}N_{12}$ $[M+H]^{+}$ 1723.9367, found 1723.9362.

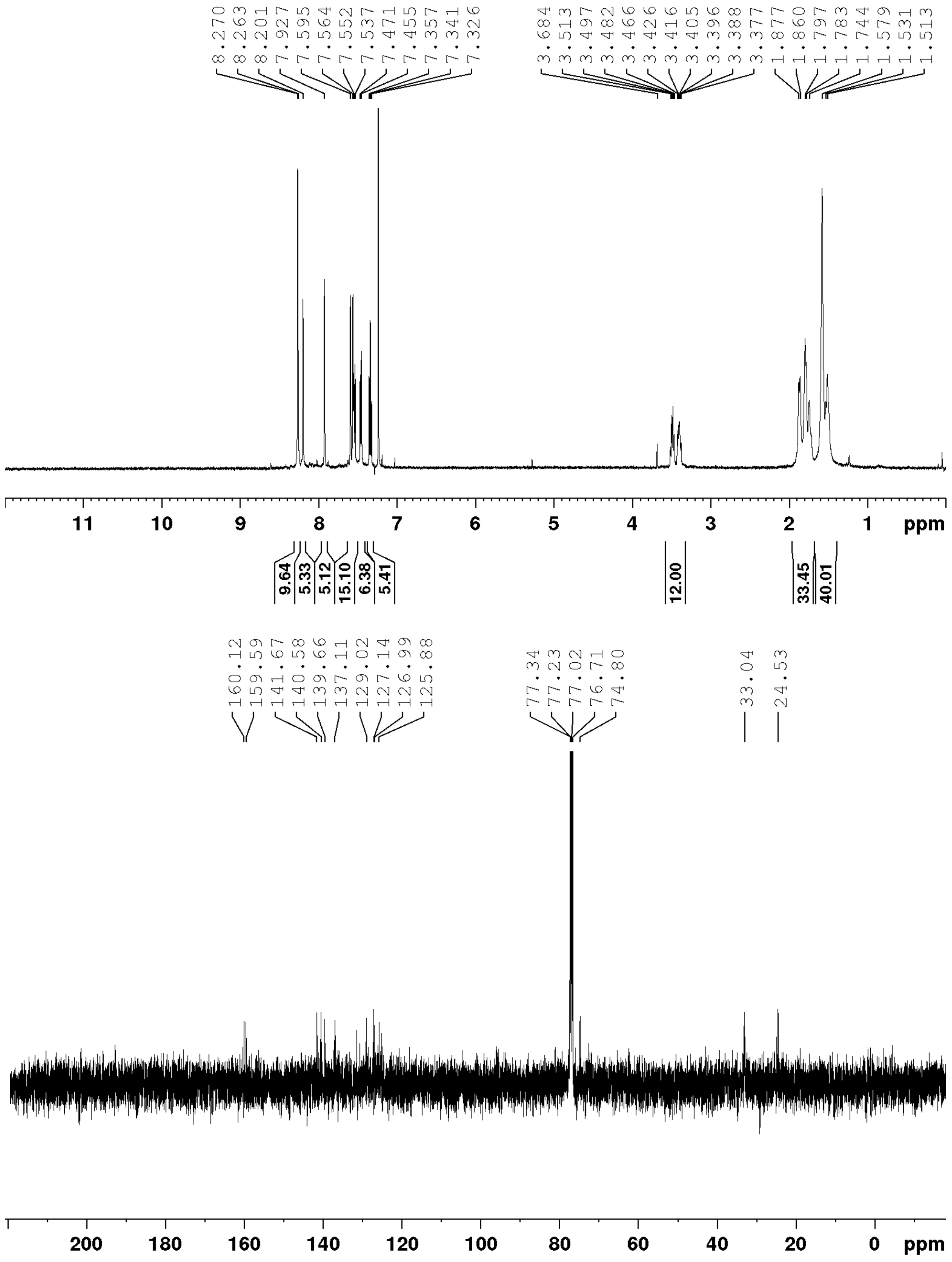

Figure S11 ^{1}H ($CDCl_3$, upper) and ^{13}C ($CDCl_3$, lower) NMR of cage**[3B2].**

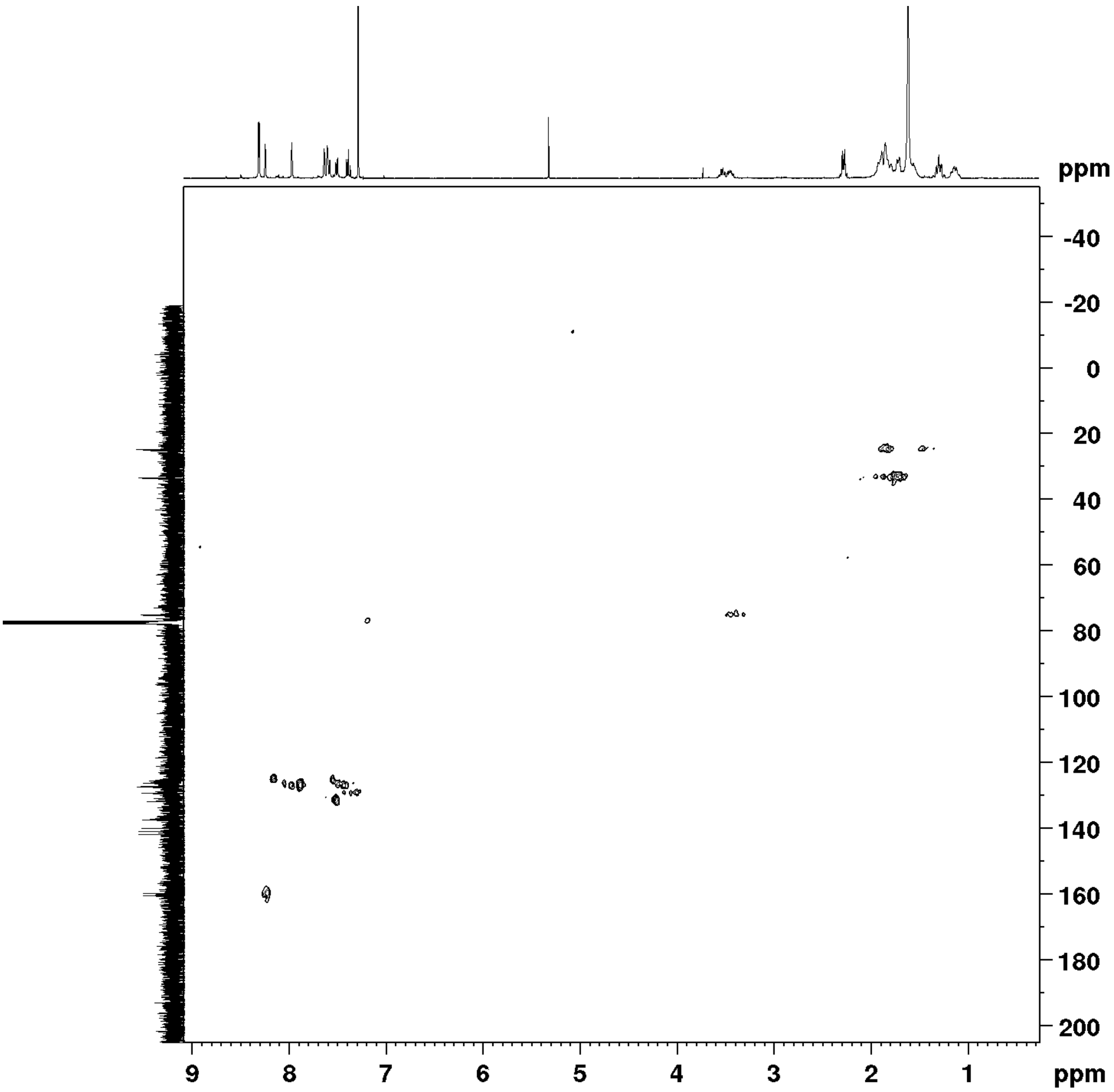

Figure S12 ^{1}H-^{13}C HSQC NMR ($CDCl_3$) of cage **[3B2]**.

Preparation of cage [3L4]

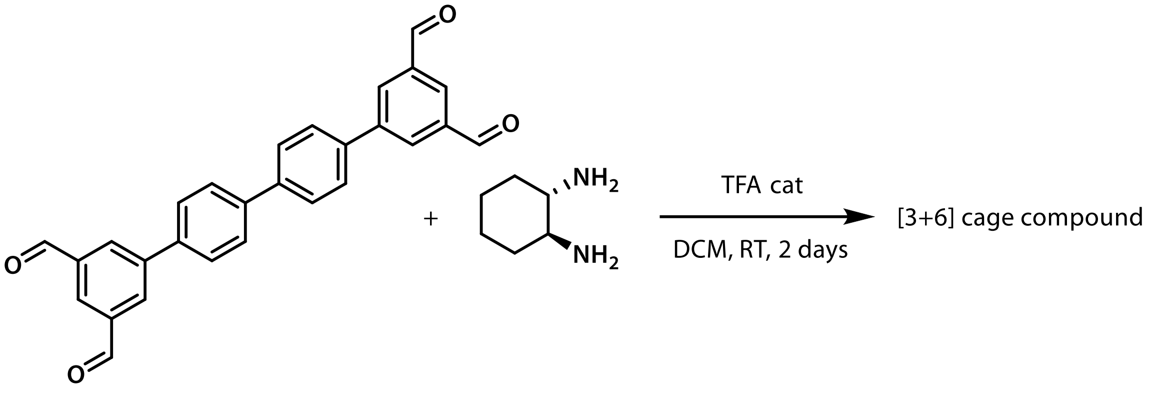

To a stirred solution of [1,1':4',1'':4'',1'''-quaterphenyl]-3,3''',5,5'''-tetracarbaldehyde (100 mg, 0.24 mmol, 1 eq.) and (1*R*,2*R*)-cyclohexane-1,2-diamine (55 mg, 0.48 mmol, 2 eq.) in DCM (20 mL), 1 drop of TFA was added. The reaction was stirred at RT for 2d. The mixture was then diluted with DCM to a 2 mg/mL concentration and the solids were filtered off. The filtrate was concentrated *in vacuo* to ~20 mL, hexane (~40 mL) was added with stirring and the resulting precipitate was collected by filtration, affording an off-white solid (20 mg, 15 %). ^{1}H NMR (400 MHz, $CDCl_3$): 8.29 (s, 6H), 8.17 (s, 6H), 7.87 (m, 12H), 7.42 (s, 6H), 7.87 (d, 12H, *J* = 8.08 Hz), 7.09 (d, 12H, *J* = 8.08 Hz), 3.51-3.45 (m, 12H), 3.24-3.18 (m, 12H), 2.06 – 1.52 (m, 48H). ^{13}C (125 MHz, $CDCl_3$): 161.44, 160.94, 141.41, 139.39. 139.11, 137.00, 136.32, 131.46, 127.52, 127.29, 125.72, 75.53, 74.00, 32.64, 31.97, 24.54. HRMS (ESI^{+}): *m/z* calcd. for $C_{120}H_{115}N_{12}$ $[M+2H]^{2+}$ 862.4717, found. 862.5544

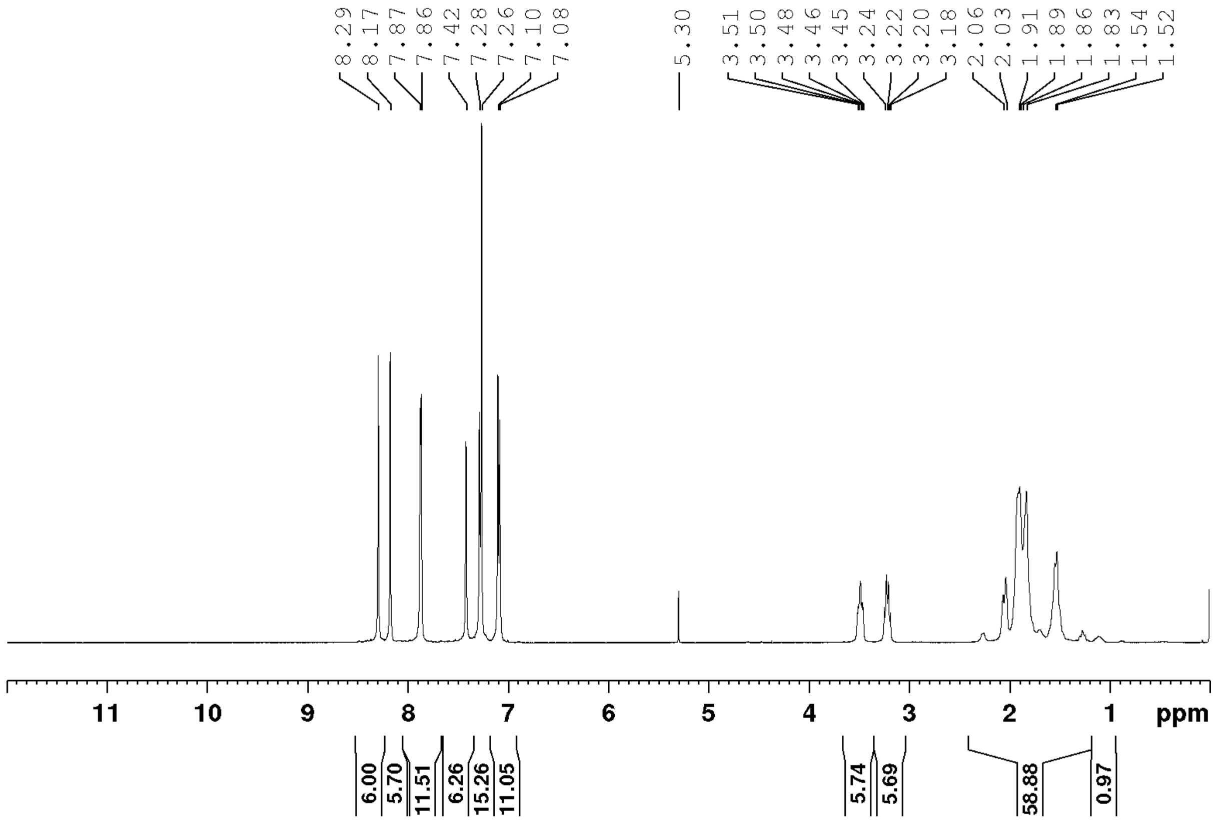

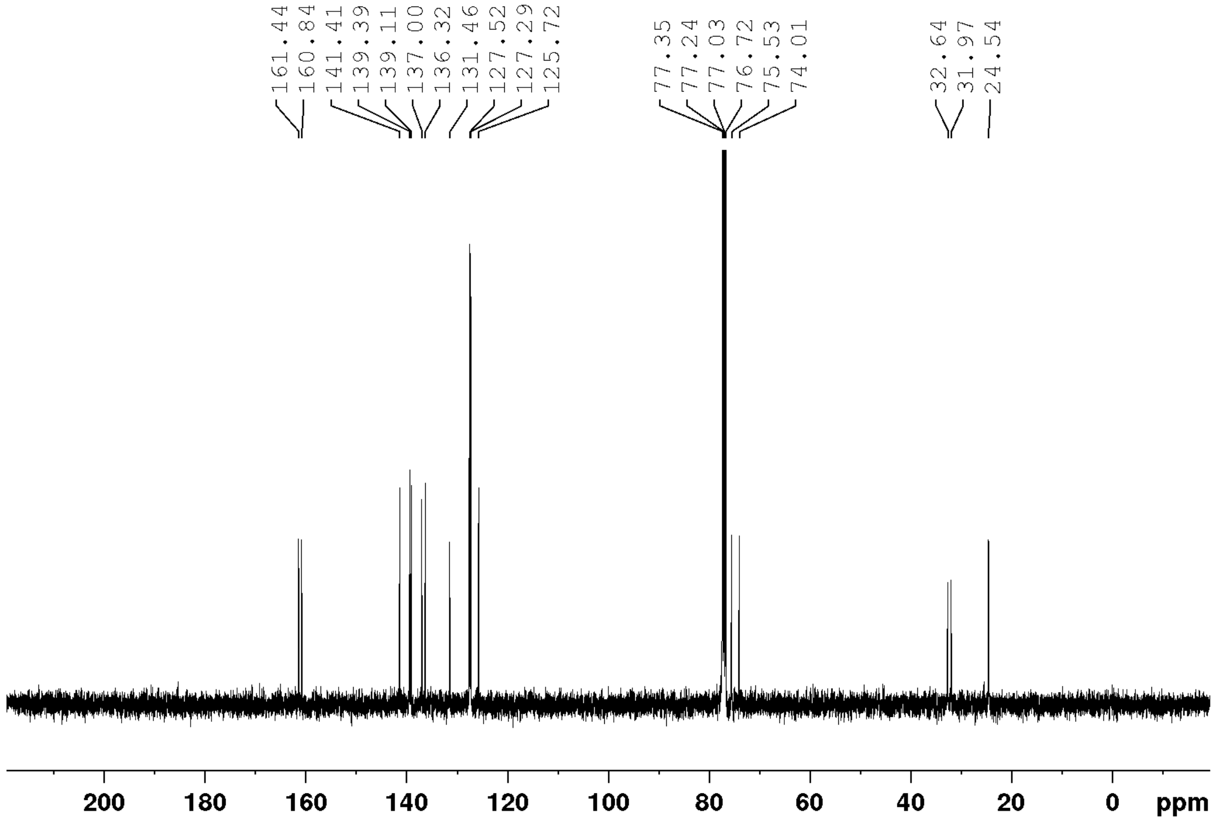

Figure S13 ^{1}H ($CDCl_3$, upper) and ^{13}C ($CDCl_3$, lower) NMR of cage **[3L4]**.

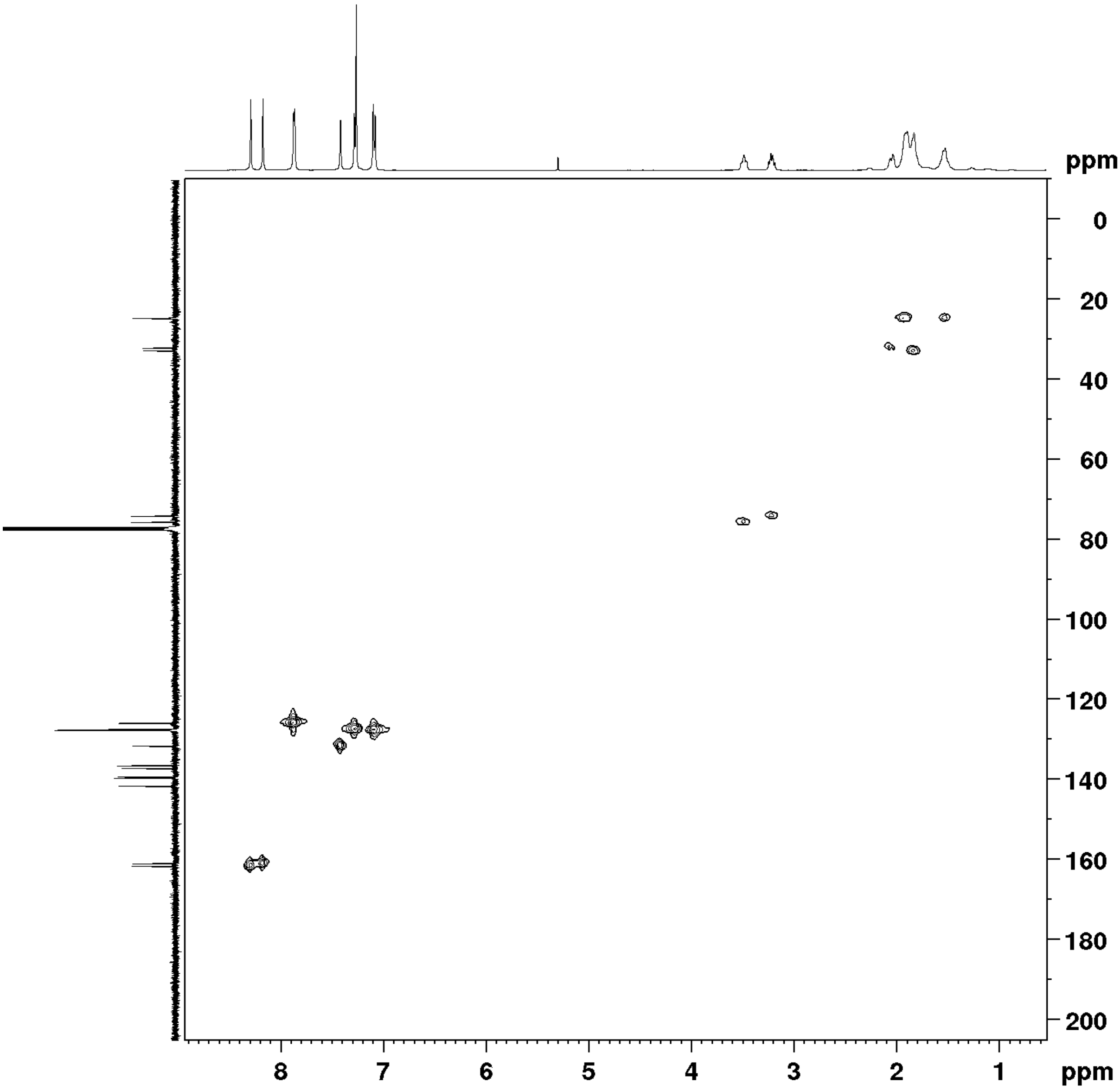

Figure S14 ^{1}H-^{13}C HSQC NMR ($CDCl_3$) of cage **[3L4].**

Attempt towards synthesis of cage [3B1] resulting in [3B1-*RS*]

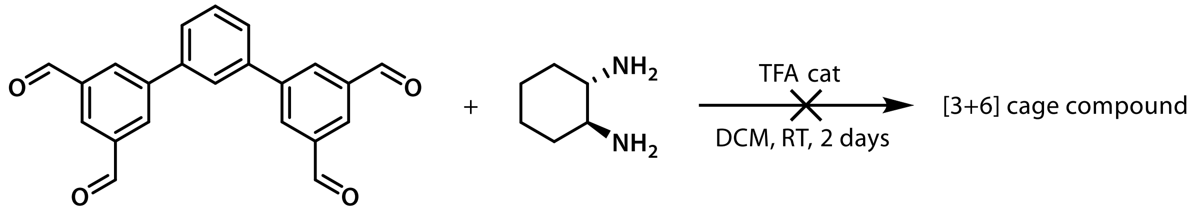

To a stirred solution of [1,1':3',1''-terphenyl]-3,3'',5,5''-tetracarbaldehyde (200 mg, 0.58 mmol, 1 eq.) and (1*R*,2*R*)-cyclohexane-1,2-diamine (133 mg, 0.48 mmol, 2 eq.) in chloroform (100 mL), 2-3 drops of TFA were added. Higher concentration reactions were found to give complex mixtures of products. The reaction was stirred at RT for 2d. The mixture was then diluted with DCM to a 2 mg/mL concentration and the solids were filtered off. The filtrate was concentrated *in vacuo* to ~20 mL, hexane (~40 mL) was added with stirring and the resulting precipitate was collected by filtration, affording an off-white solid (265 mg). Attempts to purify via recrystallization were unsuccessful. ^{1}H NMR (400 MHz, $CDCl_3$): Peaks could be identified at 8.49, 8.36, 8.26, 8.14, 8.06, 7.99, 7.94, 7.89, 7.85, 7.77, 7.66, 7.35, 7.15, 6.93, 3.41, 2.27, 1.31, but could not be integrated due to complex baseline (see **Figure S24**). IR (υ, cm^{-1}): 2926, 2855, 1641, 1594, 1447, 1374, 1201, 1135, 942, 877, 796, 699, 641, 479. MALDI-TOF: calcd. for $C_{102}H_{102}N_{12}$ $[M+H]^+$ 1495.8492, found 1850.662, 1496.168, 1157.678, 1031.704, 847.584, 853.502, 705.55, 677.521, 663.489.

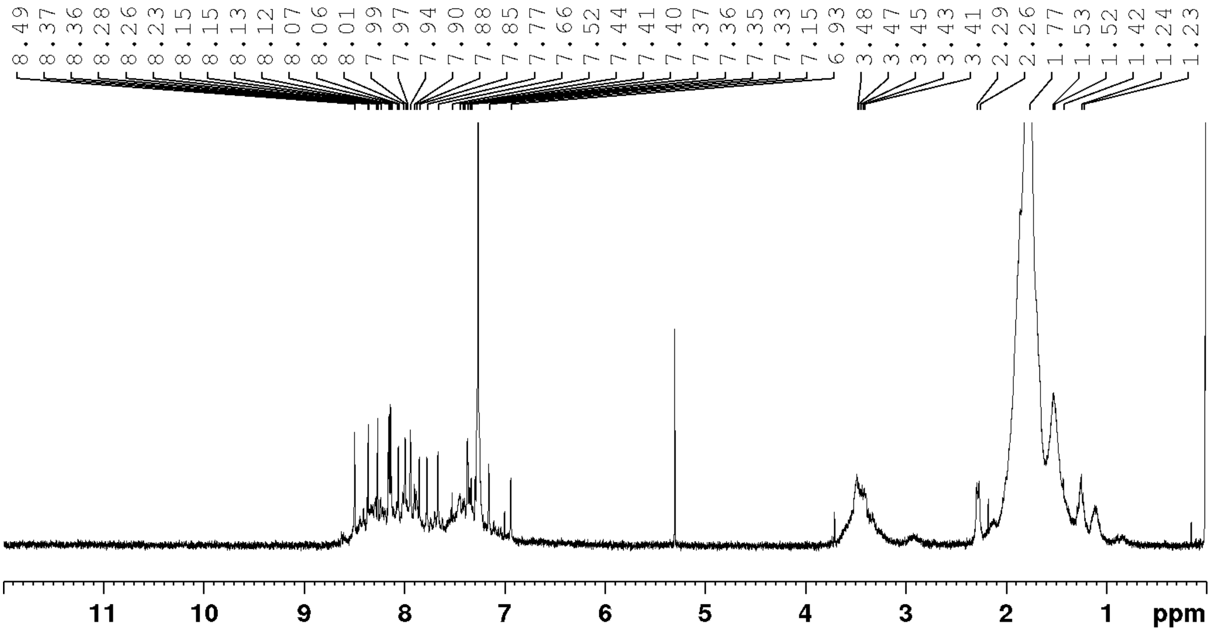

Figure S15 ^{1}H ($CDCl_3$) NMR of the mixture from the cage reaction of **B1** with ***R,R*-CHDA**.

An analogue was synthesised using (1*S*,2S)-cyclohexane-1,2-diamine and the mixture of the two stereoisomers afforded the pseudo-C_{3h}-symmetric cage **[3B1-*RS*]** under crystallisation conditions.

S3.4 General procedure for screening for mixed aldehyde cages

To a stirred solution of the corresponding linear tetraaldehyde (1 eq.), [1,1':3',1''-terphenyl]-3,3'',5,5''-tetracarbaldehyde (2 eq.) or [1,1':3',1'':3'',1'''-quaterphenyl]-3,3''',5,5'''-tetracarbaldehyde (2 eq.) and (1*R*,2*R*) -cyclohexane-1,2-diamine (6 eq.) in DCM (20 mL), 2-3 drops of TFA were added. The reaction was stirred at RT for 3d. The mixture was then diluted with DCM to a 2 mg/mL concentration and the solids were filtered off. The filtrate was concentrated *in vacuo* to ~20 mL, hexane (~40 mL) was added with stirring and the resulting precipitate was collected by filtration and characterised by UPLC-MS and NMR without further purification.

Screening mixtures containing non-linear aldehyde B1

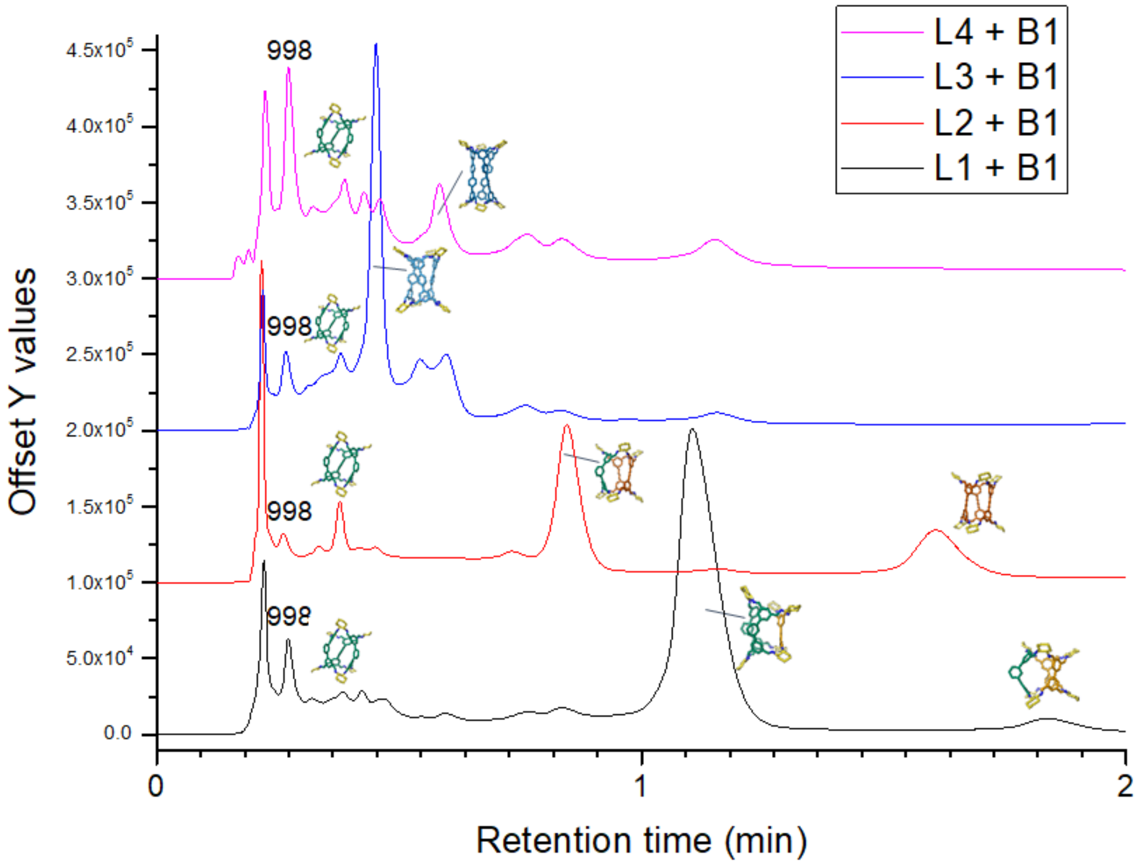

Figure S16 UPLC chromatograms for screening reactions **L1**-**L4** + **B1** + *R,R*-**CHDA**, major peaks labelled with cage structures from MS data (**Table S3**).

Table S3 Notable m/z$^+$ signals at UPLC chromatogram (see Figure S16) peaks for all mixtures tested. Question marks indicate tentative assignments; bold retention times indicate the highest peaks seen.

Aldehydes used	Retention time (min)	Notable m/z$^+$	Assignments
L1 + B1	0.2 – 0.4	998	?[2B1 + 4CHDA + H]$^+$
	0.7 – 1.0	711, 749, 1422, 1497	{**[L1+2B1]**+2H}$^{2+}$, {**[3B1]**+2H}$^{2+}$, {**[L1+2B1]**+H}$^+$, {**[3B1]**+H}$^+$
	1.0 – 1.3	711, 1422	{[L1+2B1]+2H}$^{2+}$, {[L1+2B1]+H}$^+$
	1.7 – 1.9	673, 1345	{**[2L1+B1]**+2H}$^{2+}$, {**[2L1+B1]**+H}$^+$,
L2 + B1	0.2 – 0.4	998	?[2B1 + 4CHDA + H]$^+$
	0.7 – 1.0	697, 1393	{[2L2+B1]+2H}$^{2+}$, {[2L2+B1]+H}$^+$
	1.5 – 2.0	671, 1341	{**[3L2]**+2H}$^{2+}$, {**[3L2]**+H}$^+$
L3 + B1	0.4 – 0.6	749, 1498	All [3+6] cage possibilities are the same mass, tentative assignment by retention time is seen on Figure S16.
L4 + B1	0.2 – 0.5	998	?[2B1 + 4CHDA + H]$^+$
	0.5 – 1.0	749, 863, 1497, 1727	{**[3B1]**+2H}$^{2+}$, {**[3L4]**+2H}$^{2+}$, {**[3B1]**+H}^{2}, {**[3L4]**+H}$^+$

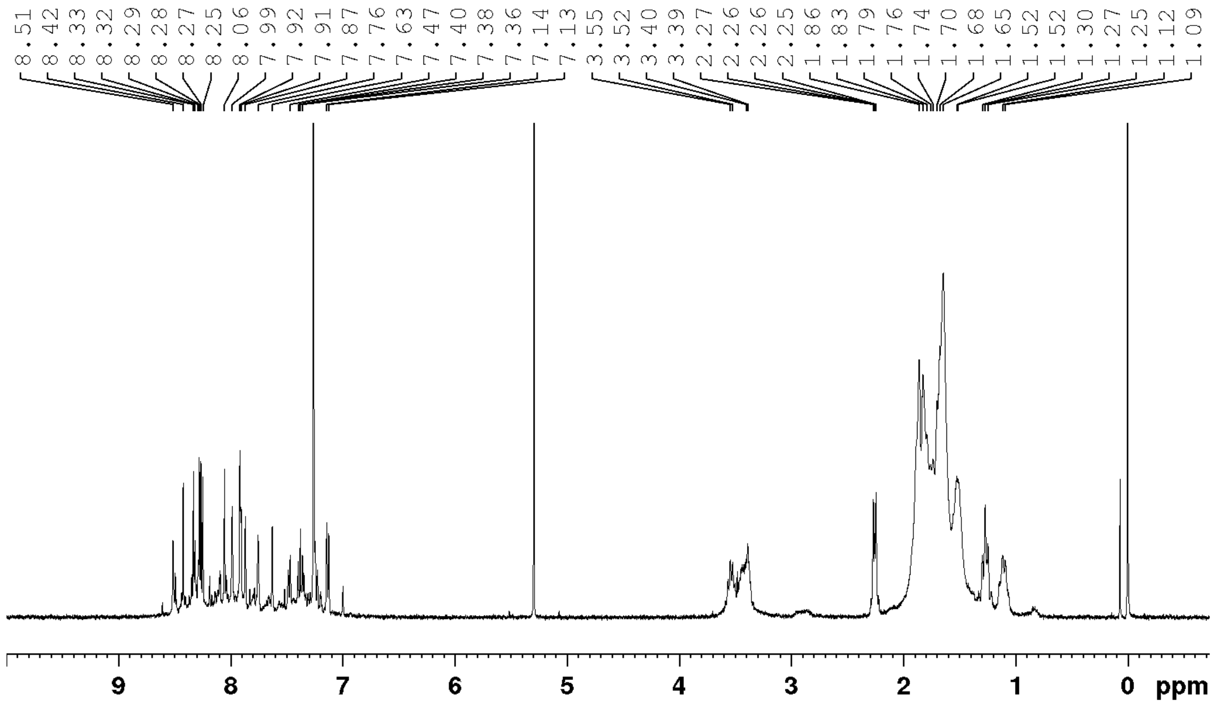

Figure S17 Crude ^{1}H ($CDCl_3$) NMR of screening reaction of **L1** + **B1** + ***R,R*-CHDA**

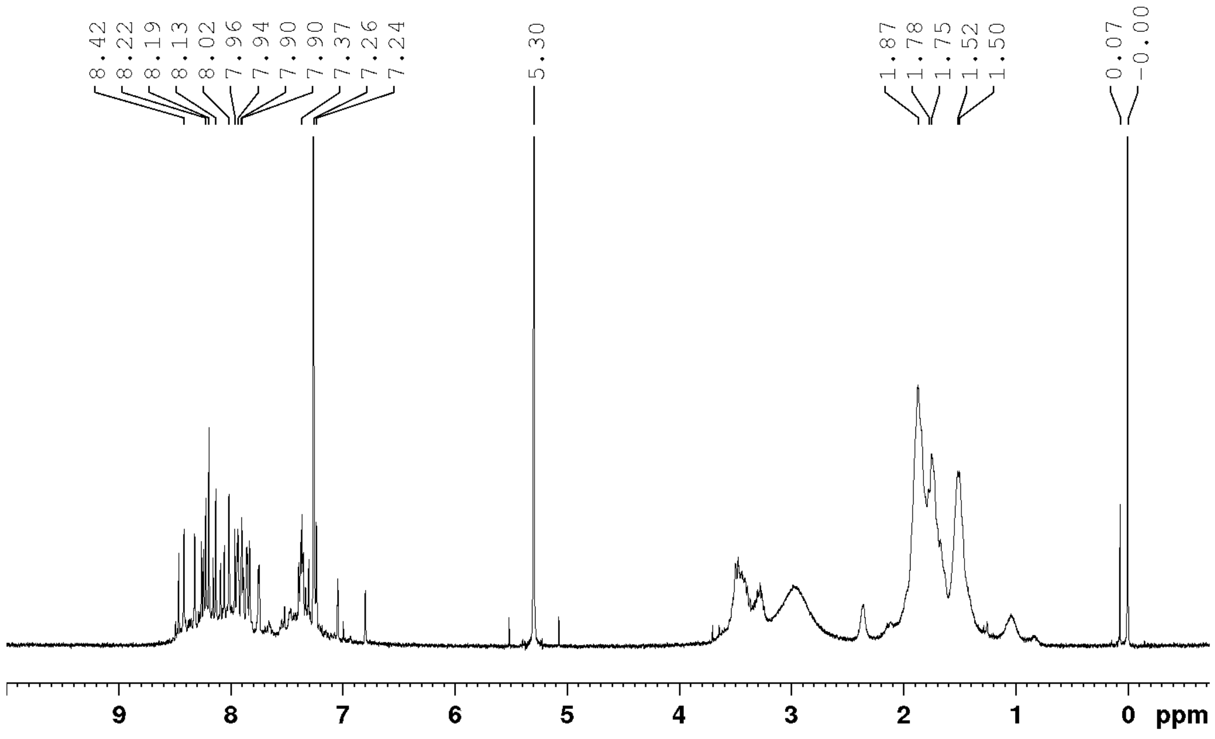

Figure S18 Crude ^{1}H ($CDCl_3$) NMR of screening reaction of **L2** + **B1** + ***R,R*-CHDA**.

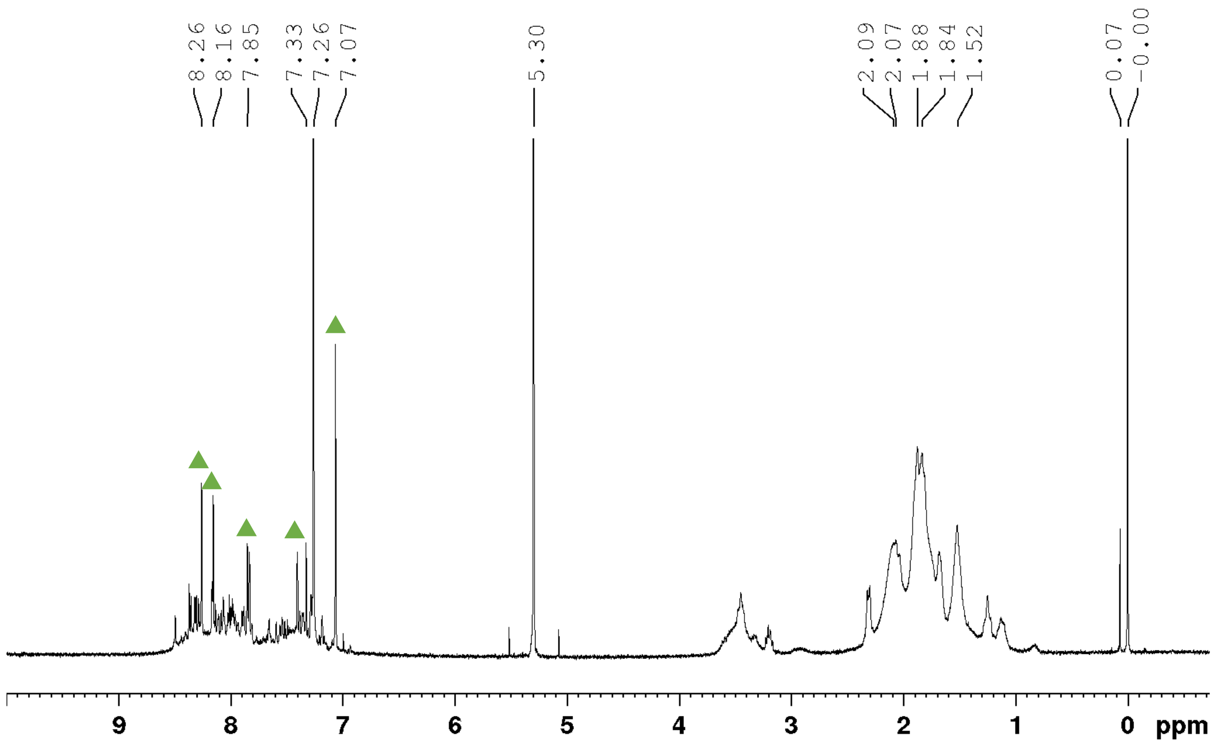

Figure S19 Crude ^{1}H ($CDCl_3$) NMR of reaction of **L3** + **B1** + *R,R*-**CHDA**. Green triangles indicate aromatic peaks arising from **[3L3]** (data compared to literature values).

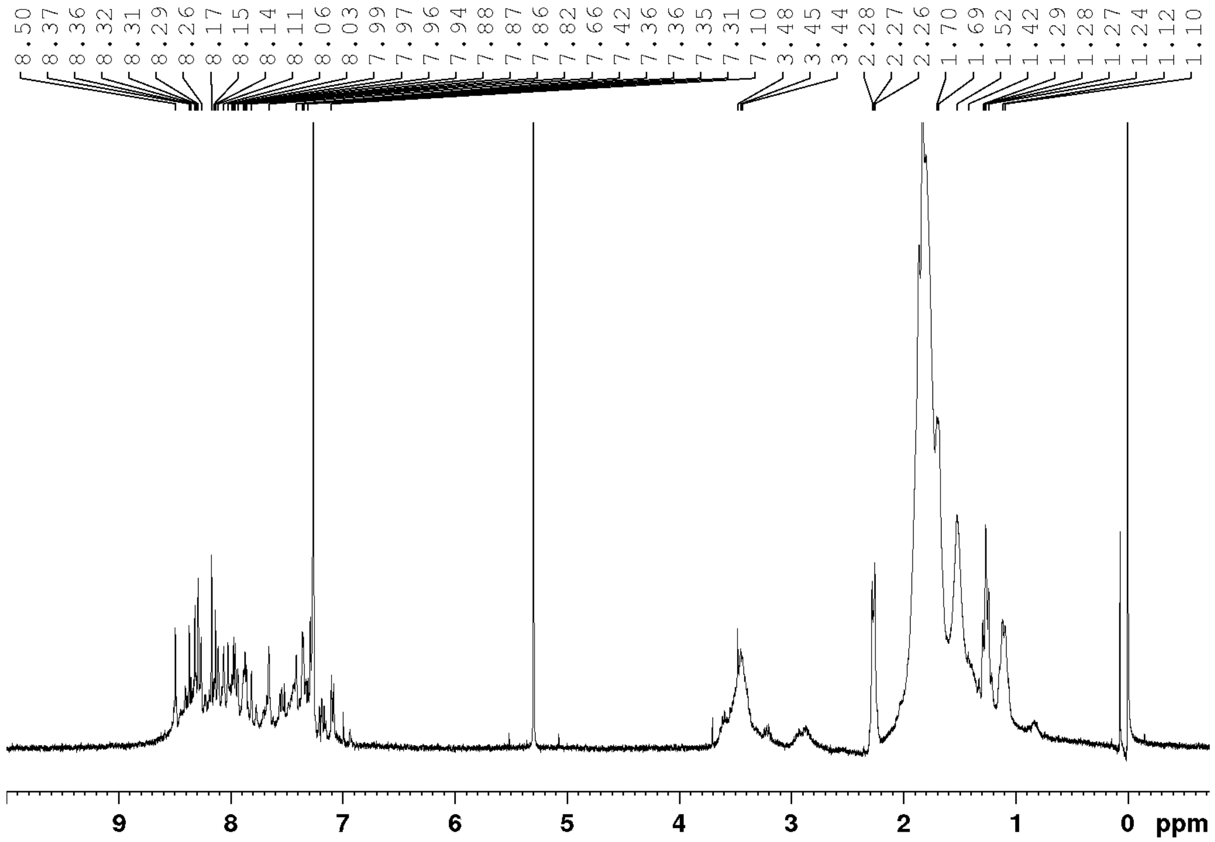

Figure S20 Crude ^{1}H ($CDCl_3$) NMR of screening reaction of **L4 + B1 +** *R,R*-**CHDA**.

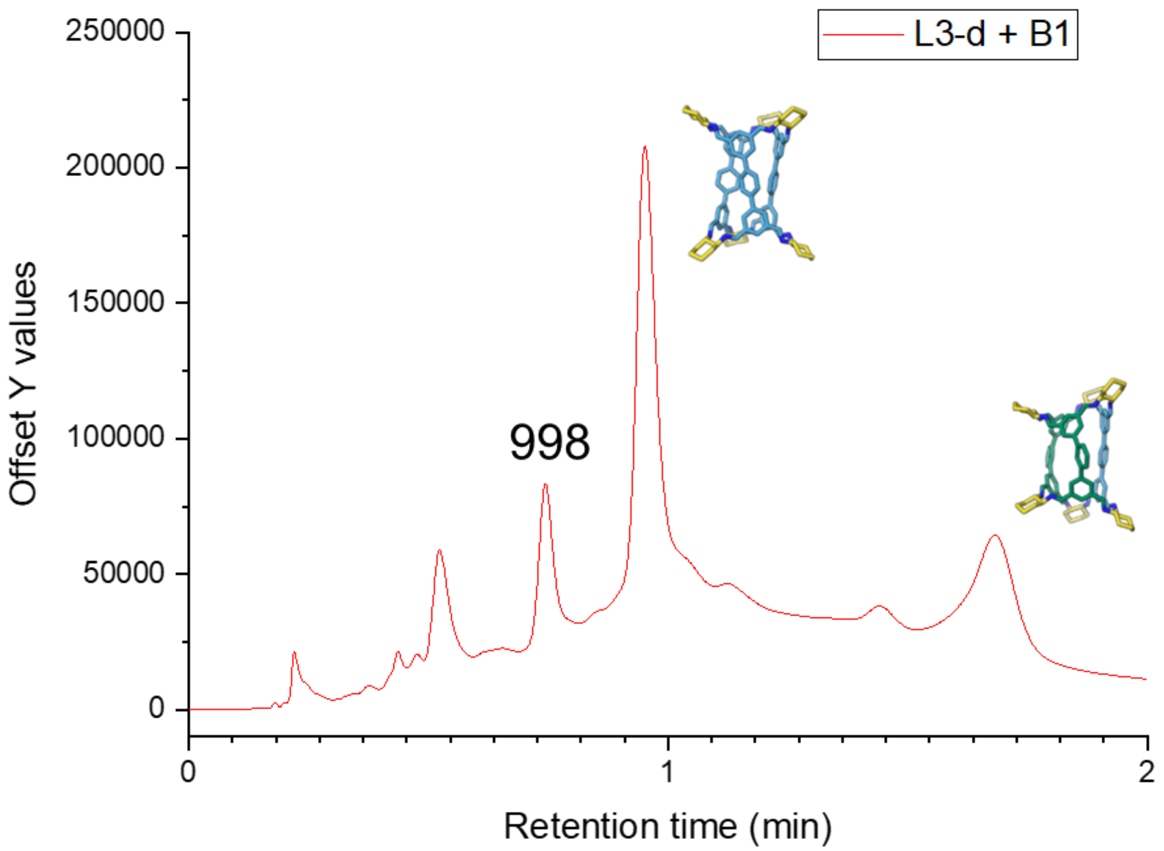

Figure S21 UPLC chromatogram for screening reaction **L3-*d4*** + **B1** + *R,R*-CHDA. Major peaks labelled with cage structures from MS data (**Table S4**).

Table S4 Notable m/z$^+$ signals at UPLC chromatogram (see Figure S21) peaks for **L3-*d4* + B1**. Question marks indicate tentative assignments; bold retention times indicate the highest peaks seen.

Aldehydes used	Retention time (min)	Notable m/z$^+$	Assignments
L3-*d4* + B1	0.7 – 0.9	998	?[2B1 + 4CHDA + H]$^+$
	0.9 – 1.3	755, 1510	{**[3L3-d]**+2H}$^{2+}$, {**[3L3-d]**+H}$^+$
	1.5 – 2.4	751, 1501	{[L3-d+2B1]+2H}$^{2+}$, {[L3-d+2B1]+H}$^+$

Screening mixtures containing non-linear aldehyde B2

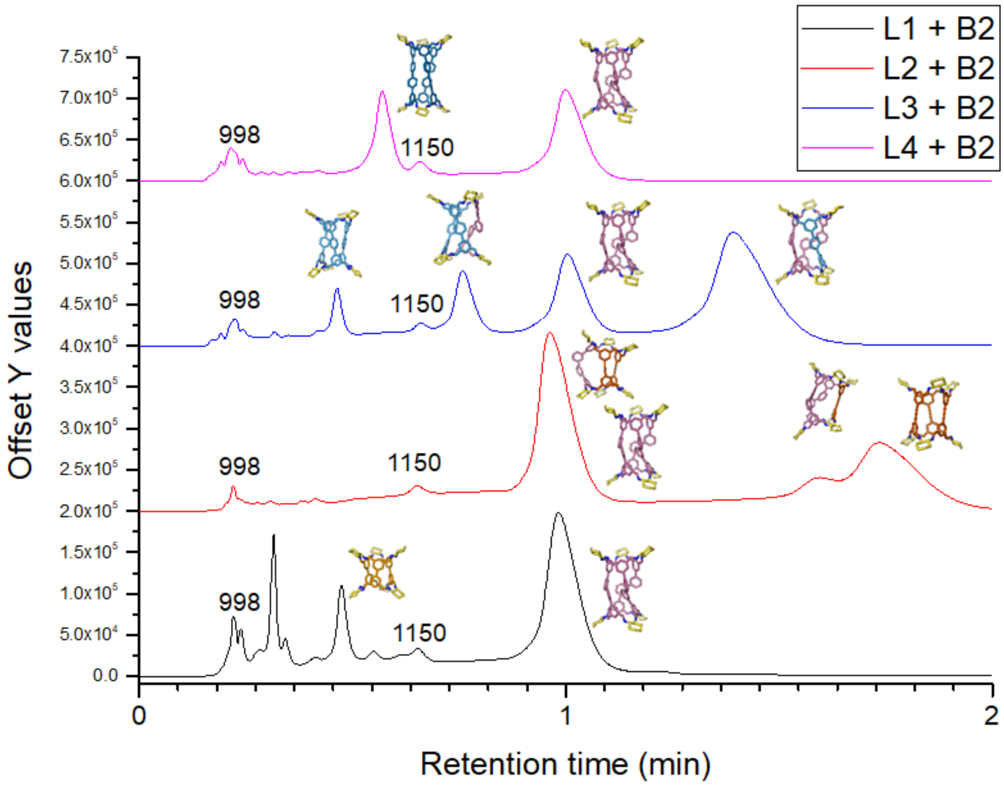

Figure S22 UPLC chromatograms for screening reactions **L1**-**L4** + **B2**, major peaks labelled with cage structures from MS data (**Table S5**).

Table S5 Notable m/z$^+$ signals at UPLC chromatogram (see Figure S22) peaks for **L1**-**L4** + **B2**. Question marks indicate tentative assignments.

Aldehydes used	Retention time (min)	Notable m/z$^+$	Assignments
L1 + B2	0.4 – 0.6	635, 1269	{**[3L1]**+2H}$^{2+}$, {**[3L1]**+H}$^+$
	0.9 – 1.2	863, 1726	{**[3B2]**+2H}$^{2+}$, {**[3B2]**+H}$^+$
L2 + B2	0.9 – 1.1	735, 864, 1469, 1726	{**[2L2+B2]**+2H}$^{2+}$, {**[3B2]**+2H}$^{2+}$, {**[2L2+B2]**+H}$^+$ {**[3B2]**+H}$^+$
	1.5 – 2.0	671, 799, 1341, 1597	{**[3L2]**+2H}$^{2+}$, {**[L2+2B2]**+2H}$^{2+}$, {**[3L2]**+H}$^+$ {[L2+2B2]+H}$^+$
L3 + B2	0.4 – 0.6	749, 1151, 1498	{**[3L3]**+2H}$^{2+}$, ?[2B2 + 4CHDA + H]$^+$, {**[3L3]**+H}$^+$
	0.6 – 0.9	787, 1574	{**[2L3+B2]**+2H}$^{2+}$, {[2L3+B2]+H}$^+$
	0.9 – 1.2	863, 1726	{**[3B2]**+2H}$^{2+}$, {**[3B2]**+H}$^+$
	1.2 – 1.7	825, 1649	{[**L3+2B2]**+2H}$^{2+}$, {[L3+2B2]+H}$^+$
L4 + B2	0.4 – 0.8	863, 1151, 1725	{**[3L4]**+2H}$^{2+}$, ?[2B2 + 4CHDA + H]$^+$, {**[3L4]**+H}$^+$
	0.8 – 1.2	863, 1726	{**[3B2]**+2H}$^{2+}$, {**[3B2]**+H}$^+$

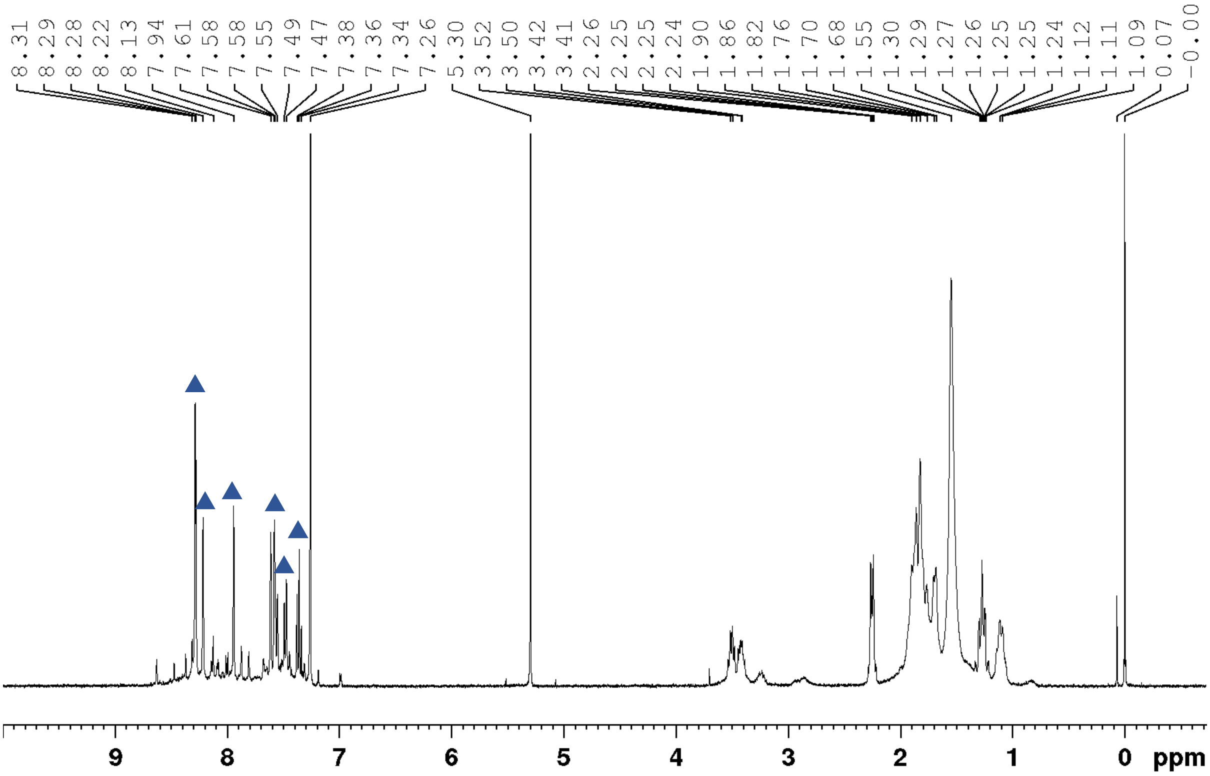

Figure S23 Crude ^{1}H ($CDCl_3$) NMR of screening reaction of **L1 + B2 + *R,R*-CHDA**. Blue triangles indicate aromatic peaks arising from cage **[3B2]** (cf. Figure S11).

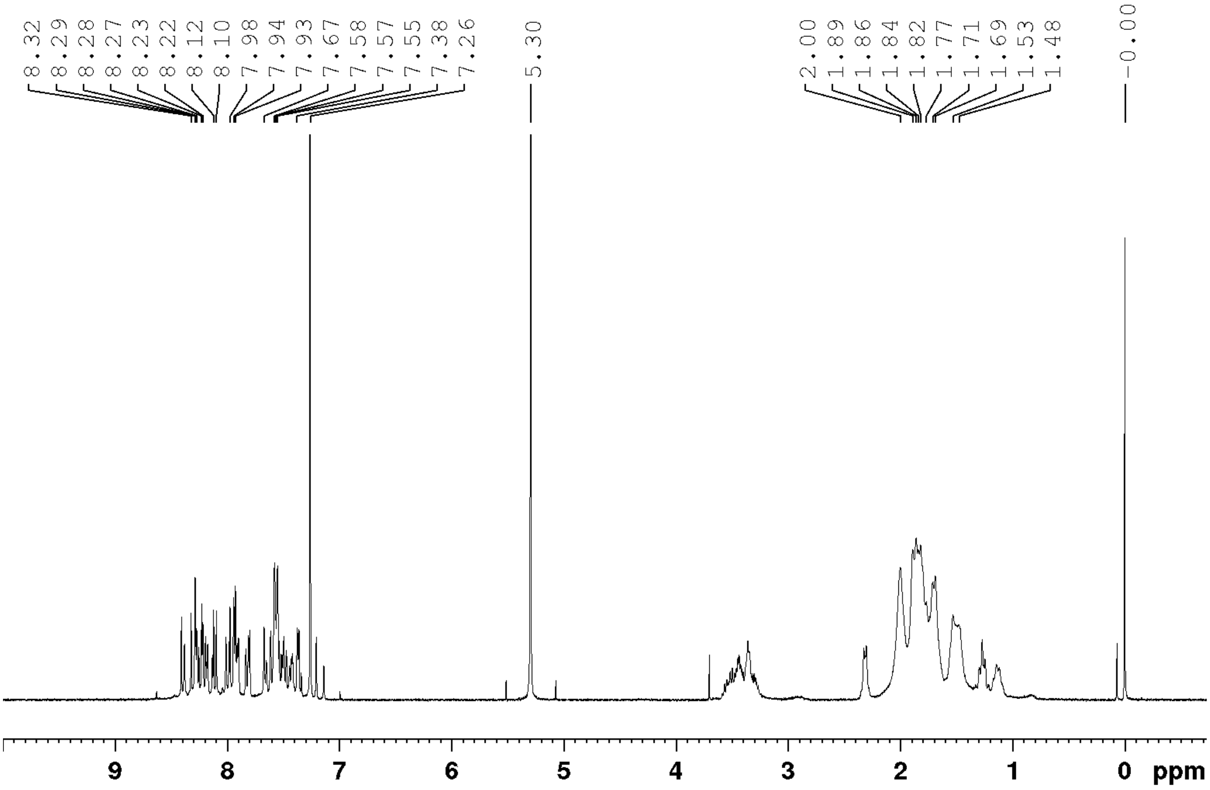

Figure S24 Crude ^{1}H ($CDCl_3$) NMR of screening reaction of **L2 + B2 + *R,R*-CHDA**.

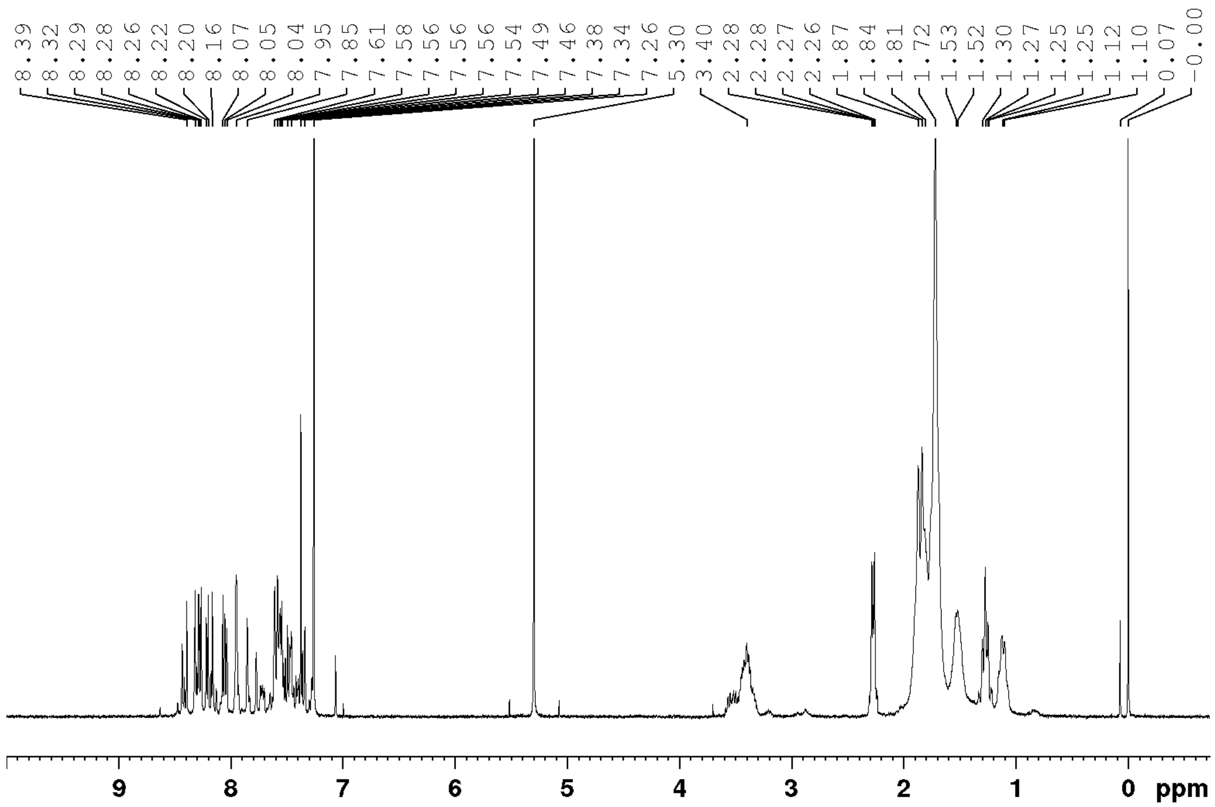

Figure S25 Crude ^{1}H ($CDCl_3$) NMR of screening reaction of **L3 + B2 + *R,R*-CHDA**.

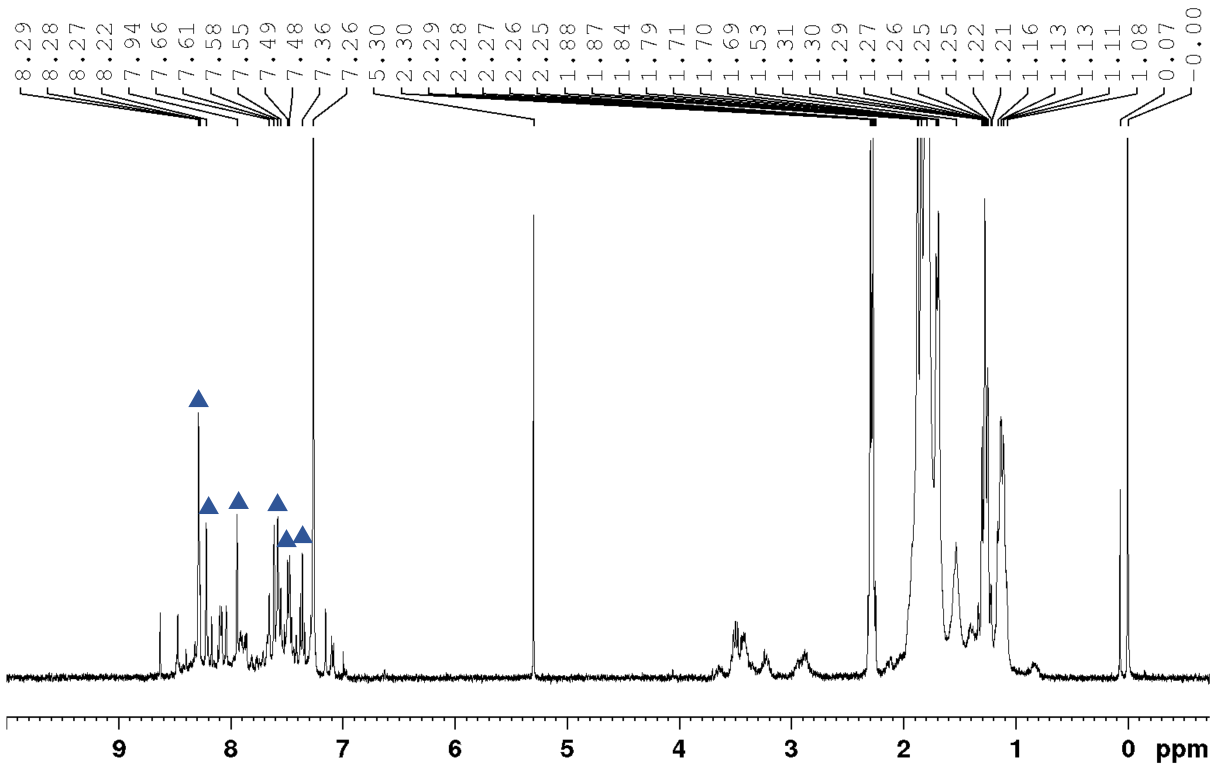

Figure S26 Crude ^{1}H ($CDCl_3$) NMR of screening reaction of **L4 + B2 + *R,R*-CHDA**. Blue triangles indicate aromatic peaks arising from cage **[3B2]** (cf. Figure S11).

S3.5 Preparation of mixed-aldehyde cages

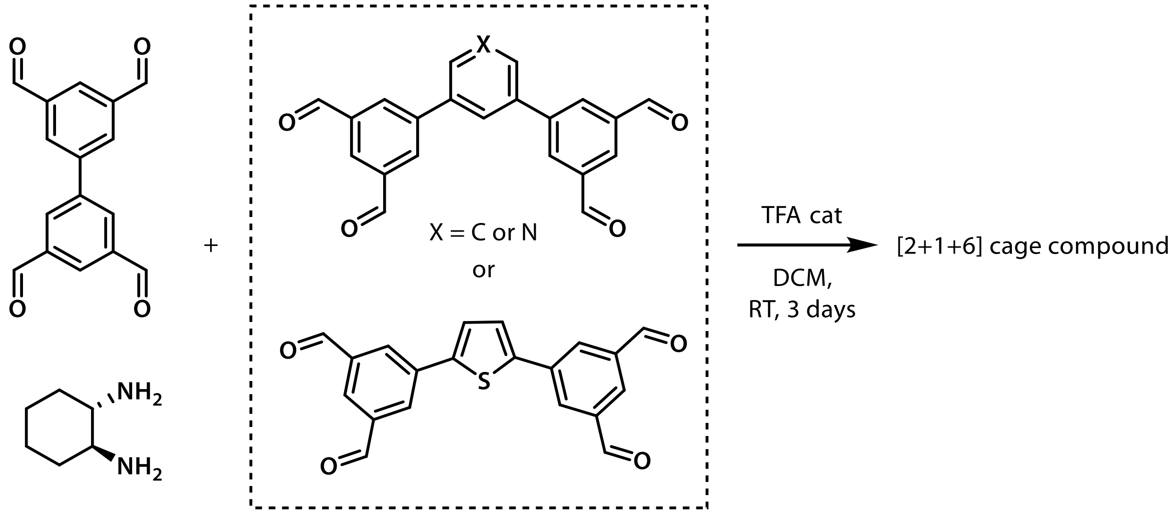

To a stirred solution of the corresponding tetraaldehyde (2 eq.), [1,1'-biphenyl]-3,3',5,5'-tetracarbaldehyde (1 eq.) and (1*R*,2*R*)- or (1*S*,2*S*)-cyclohexane-1,2-diamine (6 eq.) in DCM (20 mL), 2-3 drops of TFA were added. The reaction was stirred at RT for 3d. The mixture was then diluted with DCM to a 2 mg/mL concentration and the solids were filtered off. The filtrate was concentrated *in vacuo* to ~20 mL, hexane (~40 mL) was added with stirring and the resulting precipitate was collected by filtration or purified by preparative HPLC.

Preparation of cage [L1 + 2B1]

Following the general procedure, using [1,1':3',1''-terphenyl]-3,3'',5,5''-tetracarbaldehyde (100.0 mg, 0.29 mmol), [1,1'-biphenyl]-3,3',5,5'-tetracarbaldehyde (38.5 mg, 0.15 mmol) and (1*R*,2*R*)- or (*1S,2S*)-cyclohexane-1,2-diamine (98.2 mg, 0.86 mmol), the solid precipitate was collected by filtration as a pure compound (94.3 mg, 39% for the *R,R* cage, 88.8 mg, 37% for the *S,S* cage).

R,R cage: ^{1}H NMR (500 MHz, $CDCl_3$): 8.50 (s, 1H), 8.40 (s, 2H), 8.32 (s, 2H), 8.26-8.22 (m, 6H), 8.03 (s, 2H), 7.97 (s, 2H), 7.89-7.88 (m, 6H), 7.84 (s, 2H), 7.77 (s, 2H), 7.51 (s, 2H), 7.47-7.45 (m, 3H), 7.37-7.34 (m 2H), 7.22-7.20 (m, 2H), 7.12-7.10 (m, 4H), 3.54-3.37(m, 12H), 1.84-1.49 (m, 48H). FT-IR (ʋ, cm^{-1}): 1669, 1649, 1447, 1306, 1201, 1132, 830, 700. HRMS (ESI$^+$): *m/z* calcd. for $C_{96}H_{99}N_{12}$ [M+H]$^+$ 1420.8188, found 1420.8123.

S,S cage: ^{1}H NMR (500 MHz, $CDCl_3$): 8.52 (s, 1H), 8.42 (s, 2H), 8.32 (s, 2H), 8.28-8.25 (m, 6H), 8.05 (s, 2H), 7.98 (s, 2H), 7.95-7.91 (m, 6H), 7.87 (s, 2H), 7.76 (s, 2H), 7.61 (s, 2H), 7.51-7.45 (m, 3H), 7.39-7.35 (m 2H), 7.24-7.22 (m, 2H), 7.14-7.13 (m, 4H), 3.56-3.39 (m, 12H), 1.86-1.53 (m, 48H). FT-IR (ʋ, cm^{-1}): 1670, 1644, 1201, 1140, 906, 730. HRMS (ESI$^+$): *m/z* calcd. for $C_{96}H_{99}N_{12}$ [M+H]$^+$ 1420.8188, found 1420.8119.

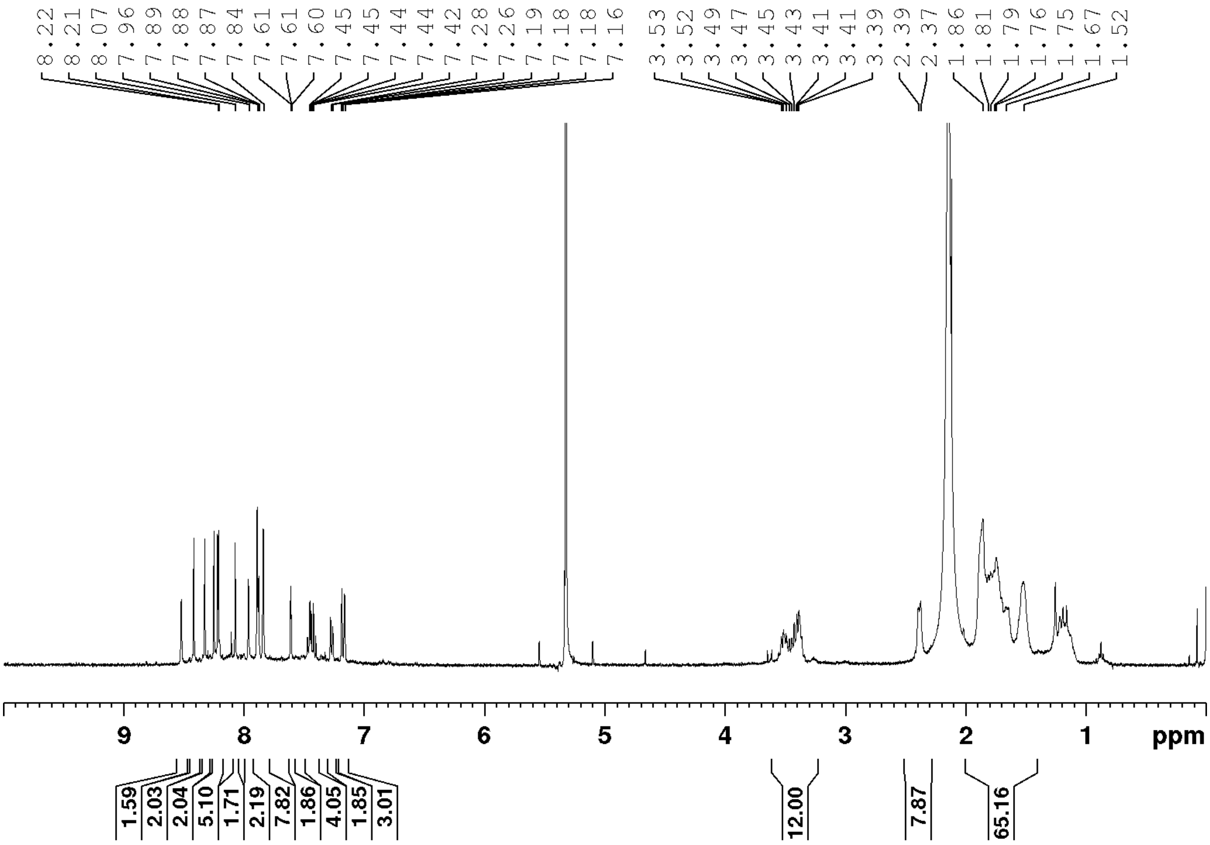

Figure S27 ^{1}H (CD_2Cl_2) NMR of cage **[L1 + 2B1]**.

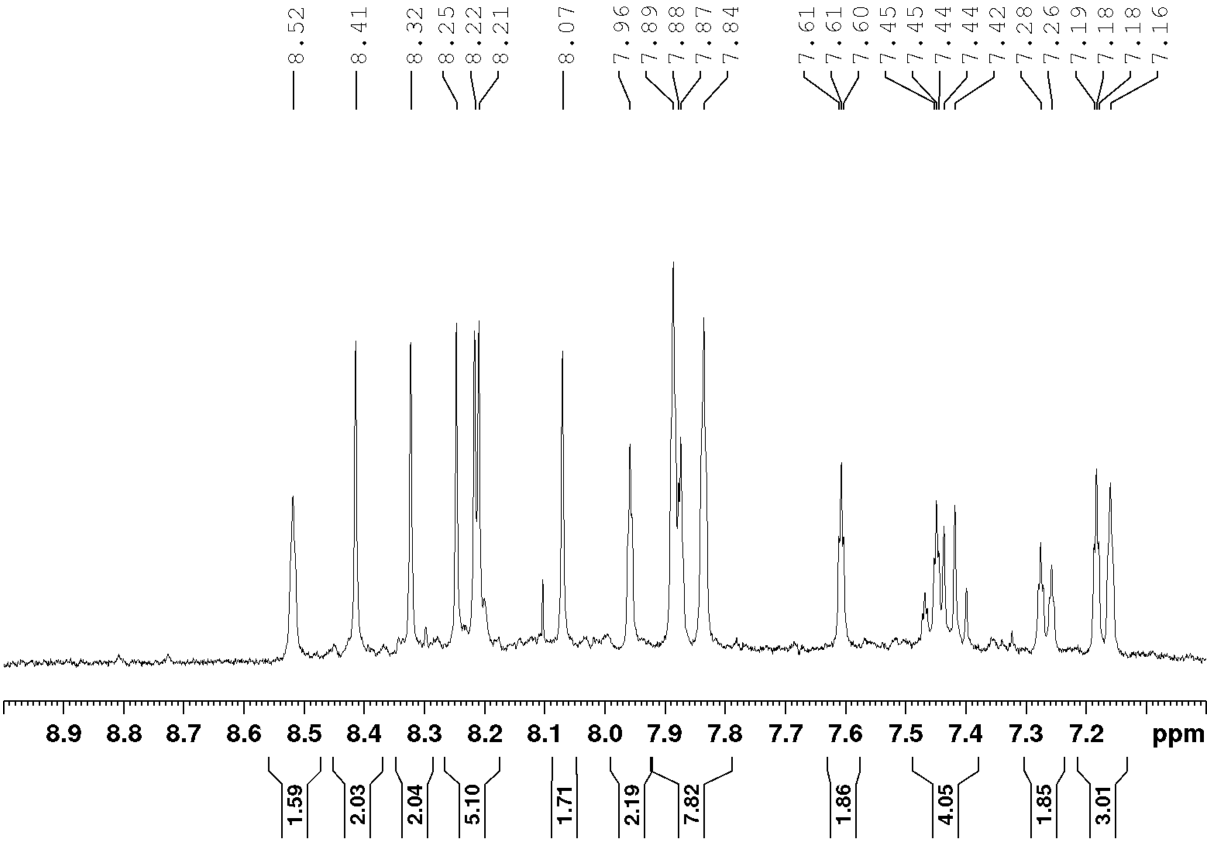

Figure S28 Aromatic region of ^{1}H (CD_2Cl_2) NMR of reaction of cage **[L1 + 2B1]**.

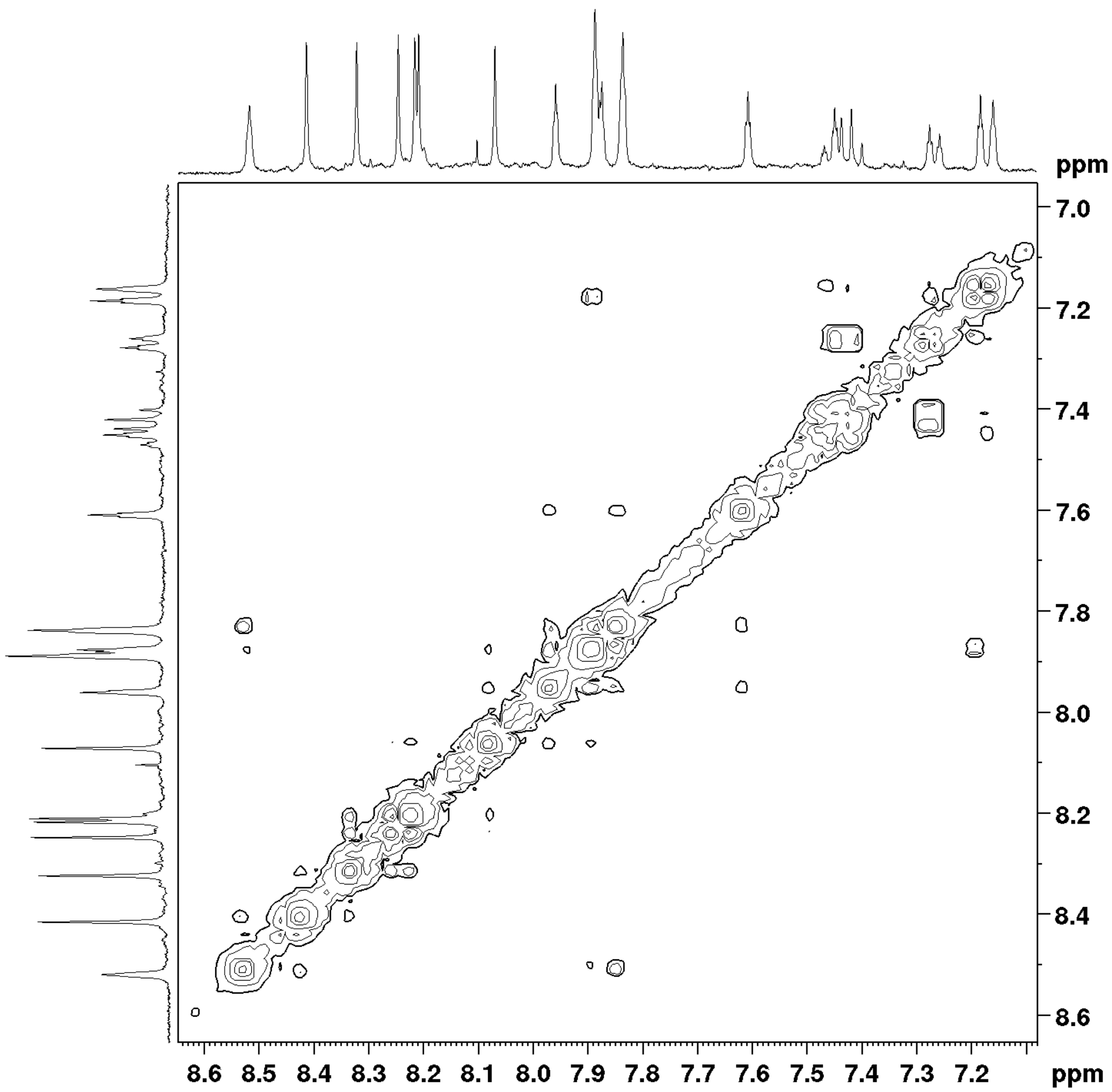

Figure S29 Aromatic region ^{1}H-^{1}H COSY (CD_2Cl_2) NMR of reaction of cage **[L1 + 2B1]**.

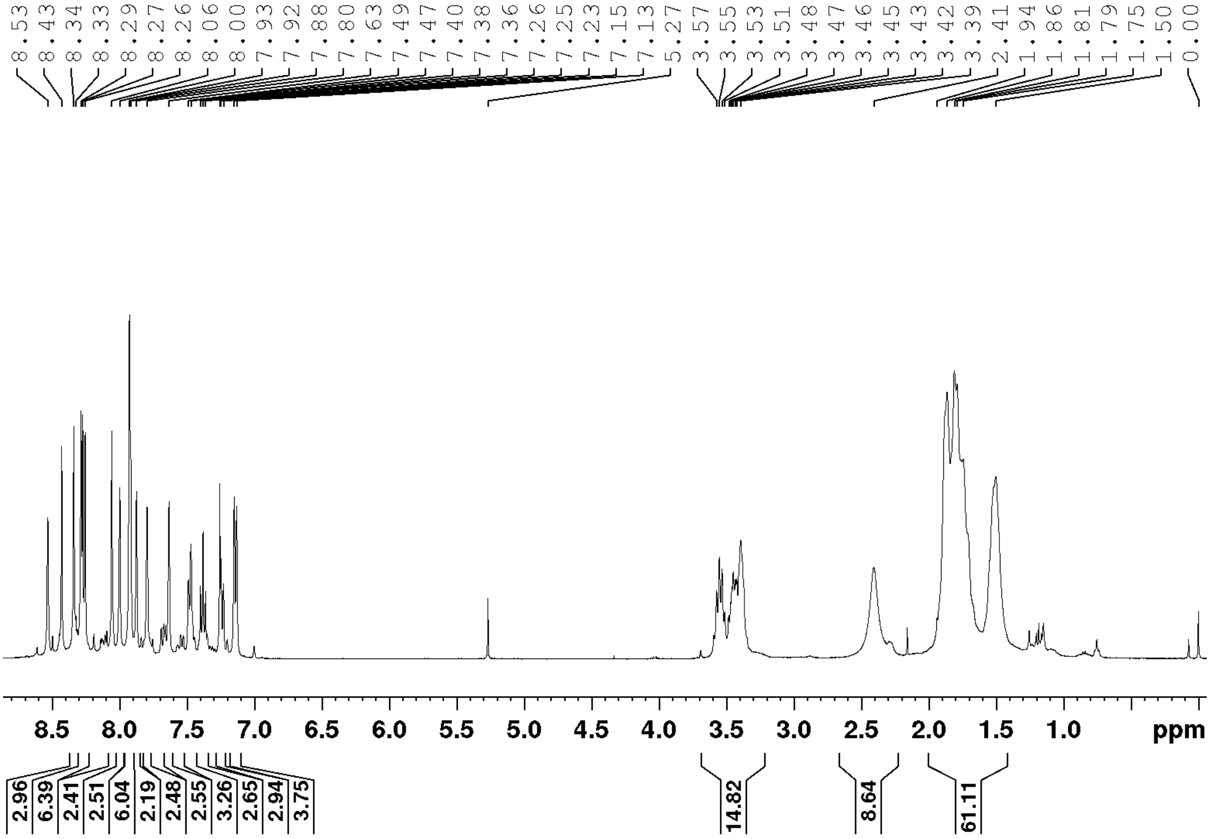

Figure S30 ^{1}H ($CDCl_3$) NMR of reaction of cage **[L1 + 2B1]**.

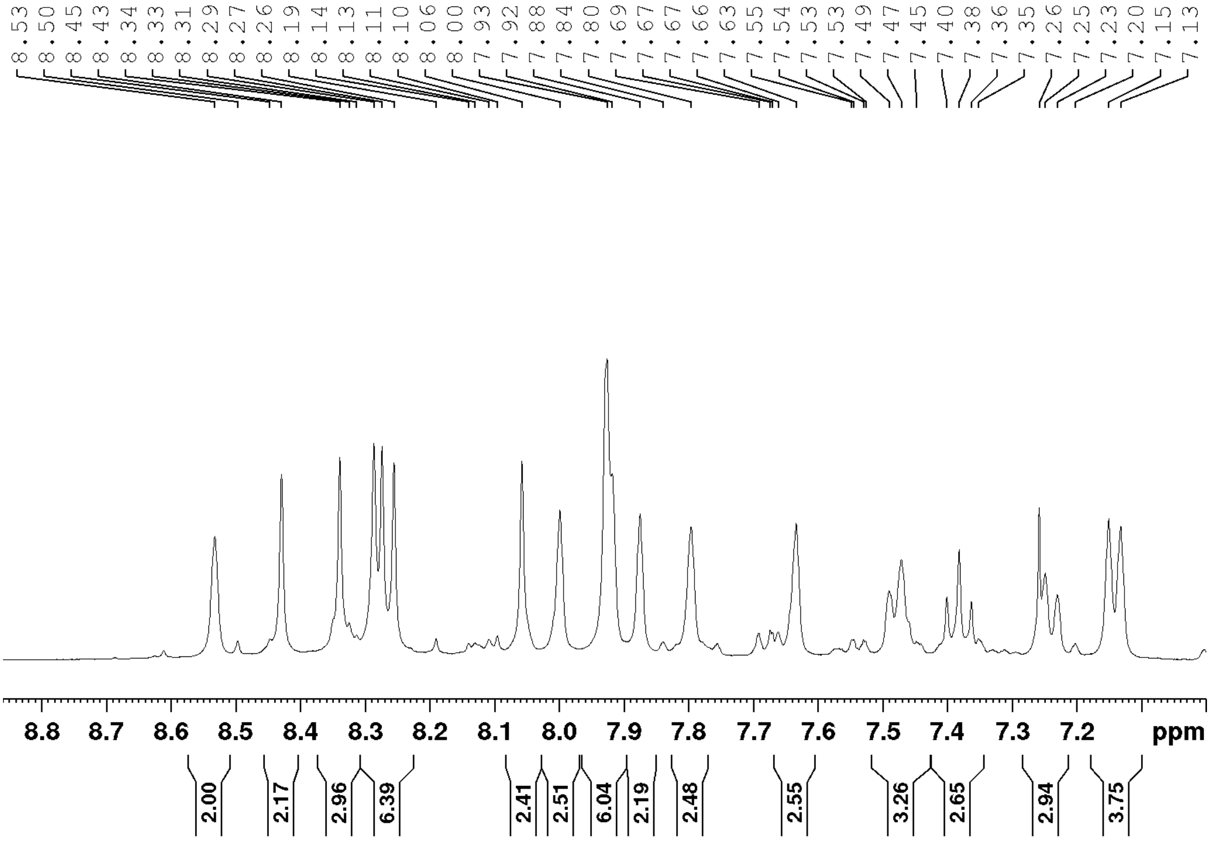

Figure S31 Aromatic region of ^{1}H ($CDCl_3$) NMR of reaction of cage **[L1 + 2B1]**.

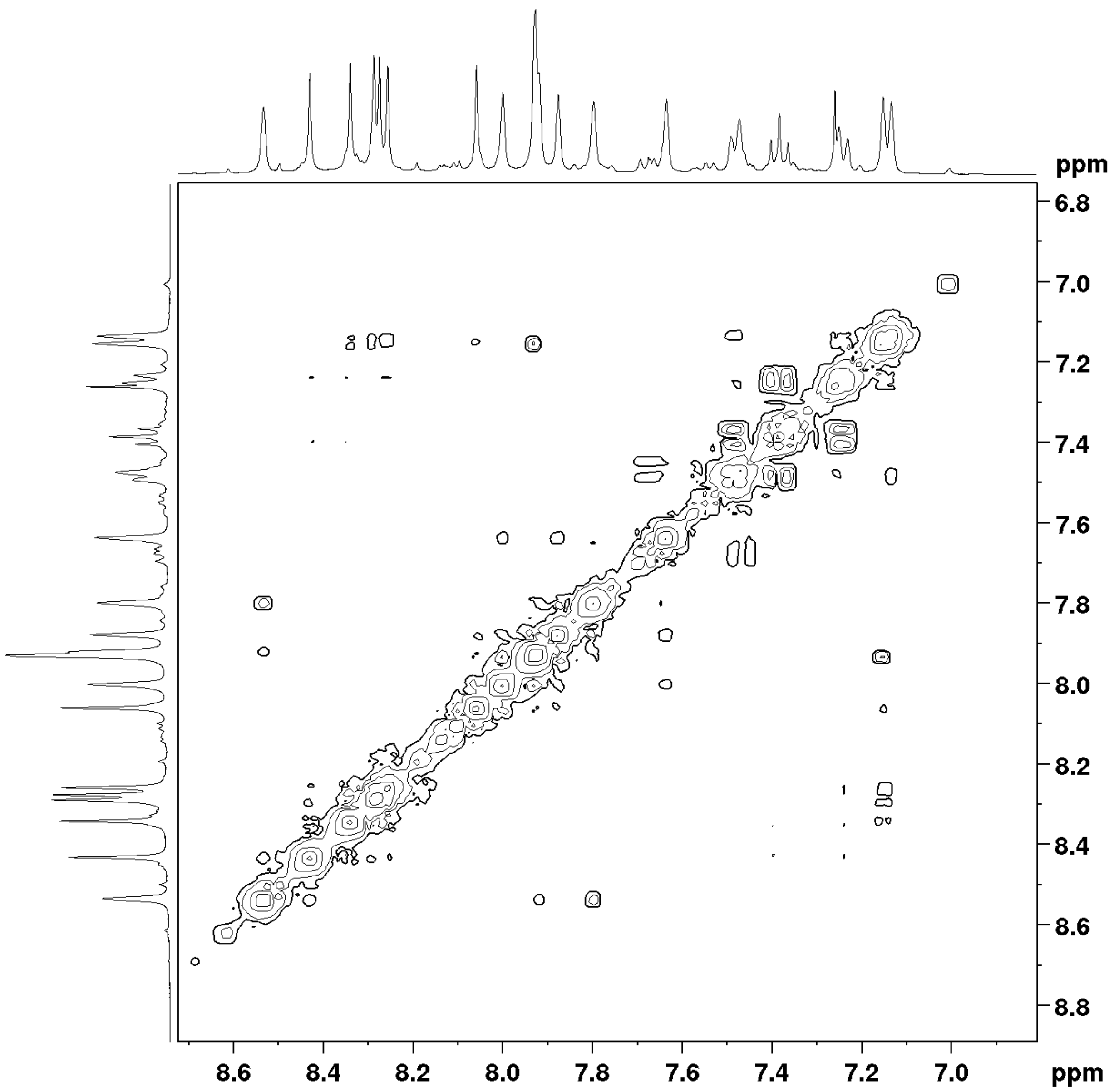

Figure S32 Aromatic region of ^{1}H-^{1}H COSY ($CDCl_3$) NMR of reaction of cage **[L1 + 2B1]**.

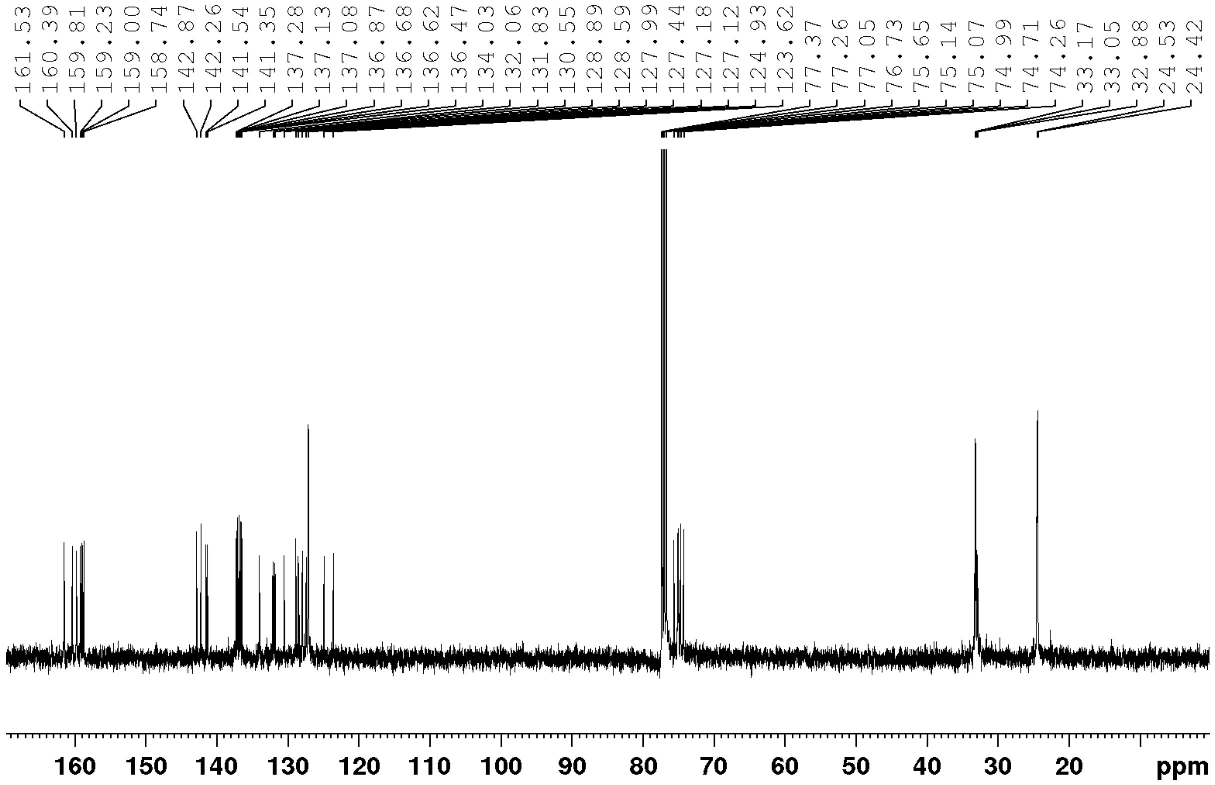

Figure S33 Aromatic region ^{13}C ($CDCl_3$) NMR of reaction of cage **[L1 + 2B1]**.

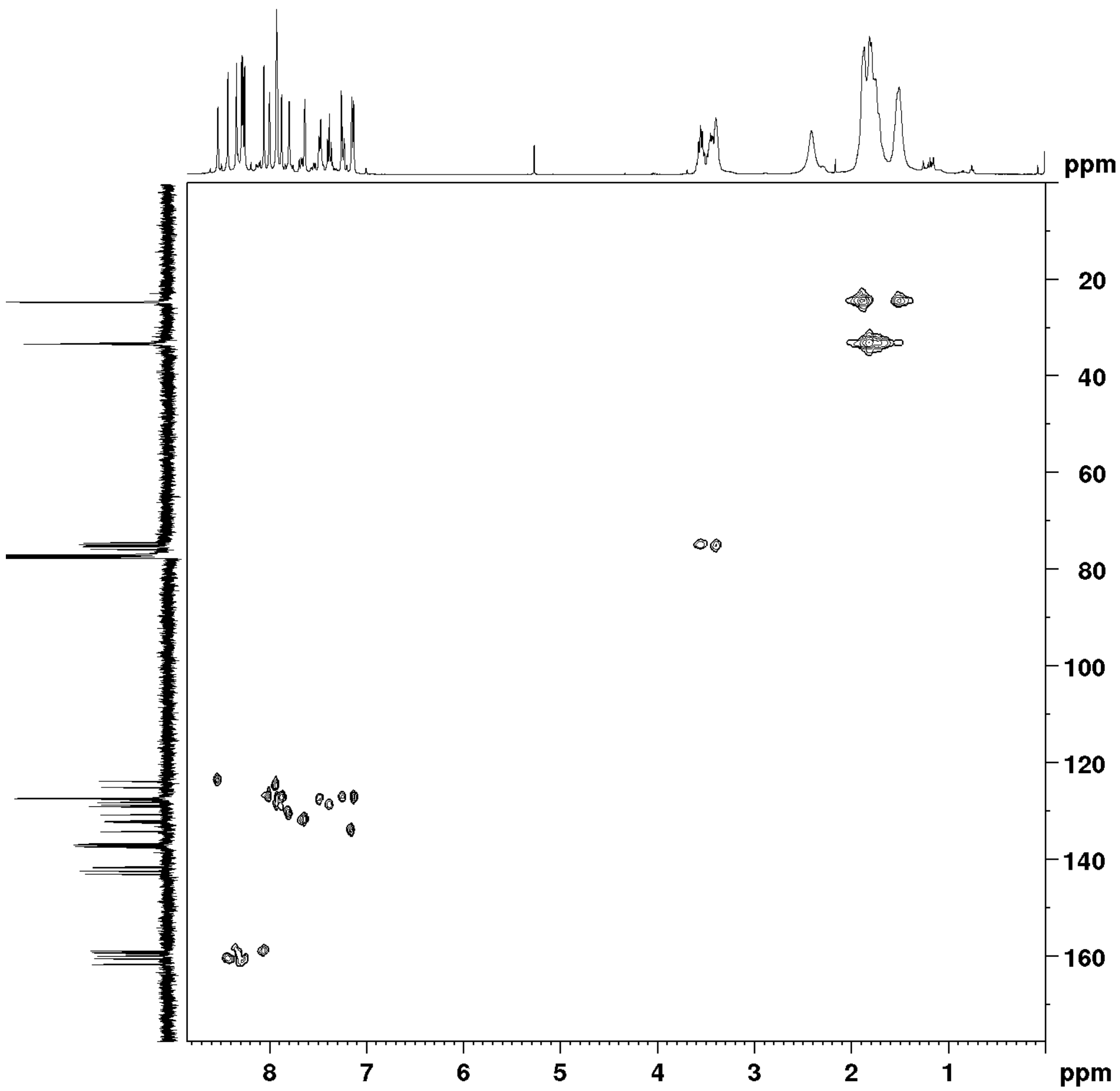

Figure S34 ^{1}H-^{13}C HSQC ($CDCl_3$) NMR of reaction of cage **[L1 + 2B1]**.

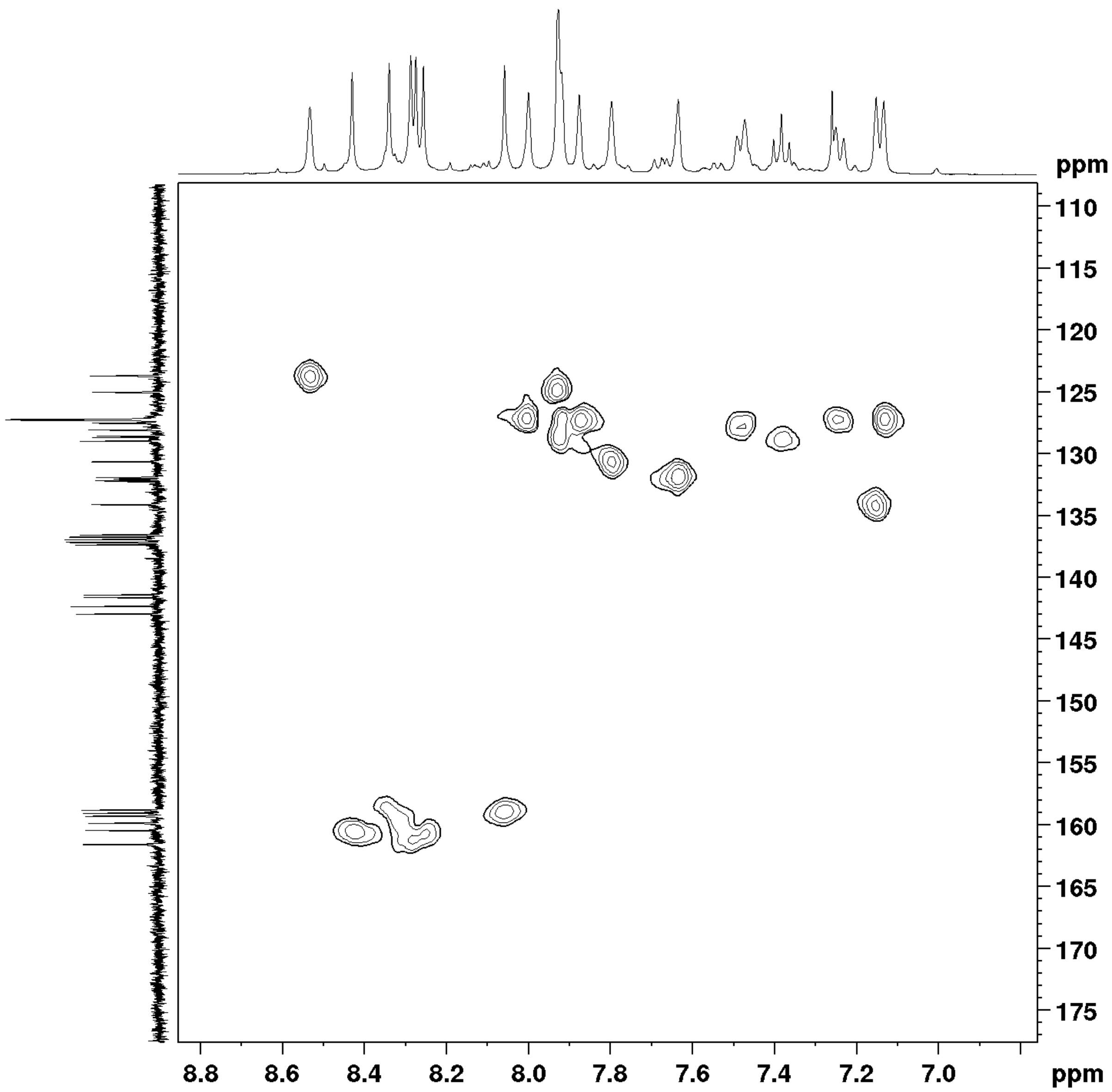

Figure S35 Aromatic region ^{1}H-^{13}C HSQC ($CDCl_3$) NMR of cage **[L1 + 2B1]**.

Preparation of cage [L1 + 2B1S]

Following the general procedure, using 5,5'-(thiophene-2,5-diyl)diisophthalaldehyde (100.0 mg, 0.29 mmol), [1,1'-biphenyl]-3,3',5,5'-tetracarbaldehyde (38.5 mg, 0.15 mmol) and (1*R*,2*R*)- or (*1S,2S*)-cyclohexane-1,2-diamine (98.2 mg, 0.86 mmol), the solid precipitate was collected by filtration and purified by preparative HPLC that afforded a light yellow solid (74.1mg, 31% for the *R,R* cage, 119.6 mg, 50% for the *S,S* cage).

R,R cage: ^{1}H NMR (500 MHz, $CDCl_3$): 8.44 (s, 2H), 8.29-8.28 (m, 4H), 8.17-8.15 (m, 6H), 8.11-8.10 (m, 4H), 8.04 (s, 2H), 7.79-7.77 (m, 8H), 7.71-7.70 (m, 4H), 7.10 (d, 2H, *J* = 3.8 Hz), 6.85 (d, 2H, *J* = 3.8 Hz), 3.53-3.18 (m, 12H), 1.90-1.71 (m, 48H). FT-IR (υ, cm^{-1}): 2927, 2856, 1646, 1598, 1448, 12610, 1094, 875, 731. HRMS (ESI^+): *m/z* calcd. for $C_{92}H_{95}N_{12}S_2$ $[M+H]^+$ 1432.7317, found 1432.7244.

S,S cage: ^{1}H NMR (500 MHz, $CDCl_3$): 8.44 (s, 2H), 8.30-8.28 (m, 4H), 8.15-8.14 (m, 6H), 8.11-8.10 (m, 4H), 8.04 (s, 2H), 7.80-7.78 (m, 8H), 7.72-7.71 (m, 4H), 7.10 (d, 2H, *J* = 3.8 Hz), 6.85 (d, 2H, *J* = 3.8 Hz), 3.54-3.17 (m, 12H), 1.87-1.49 (m, 48H). FT-IR (υ, cm^{-1}): 2925, 2854, 1674, 1644, 1447, 1200, 1137, 908, 729. HRMS (ESI^+): *m/z* calcd. for $C_{92}H_{95}N_{12}S_2$ $[M+H]^+$ 1432.7317, found 1432.7296.

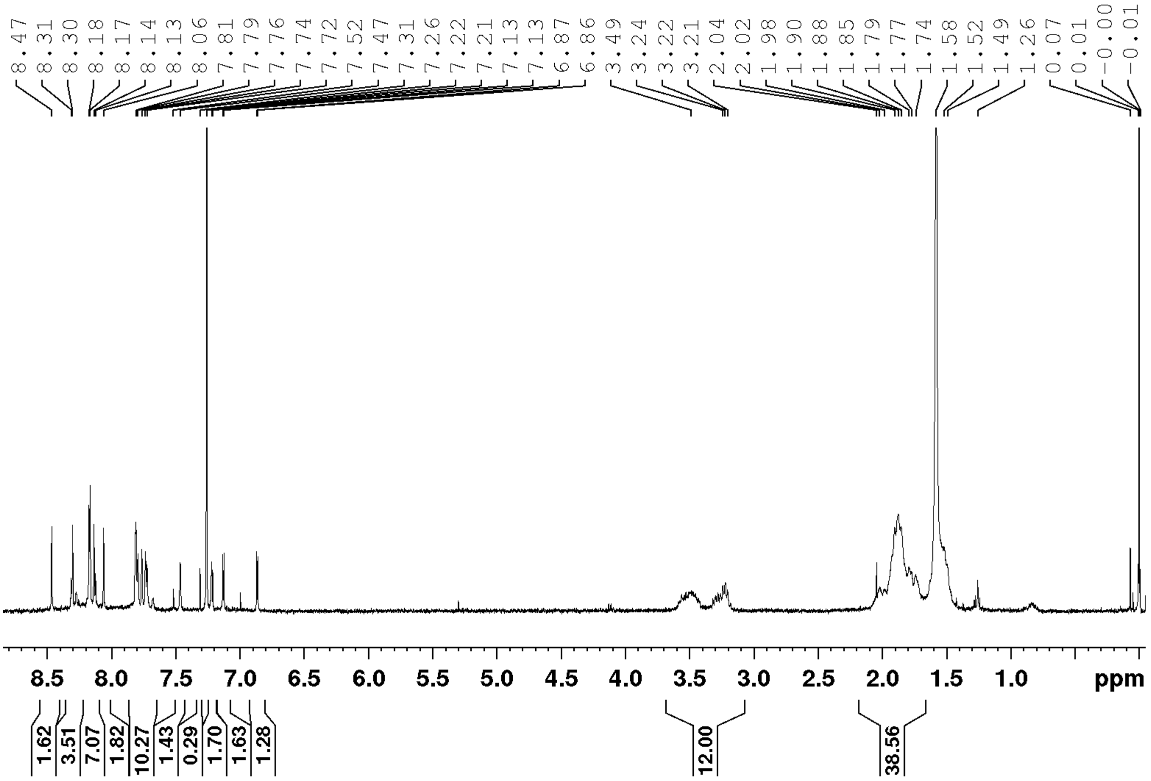

Figure S36 ^{1}H ($CDCl_3$) NMR of cage **[L1 + 2B1S]**.

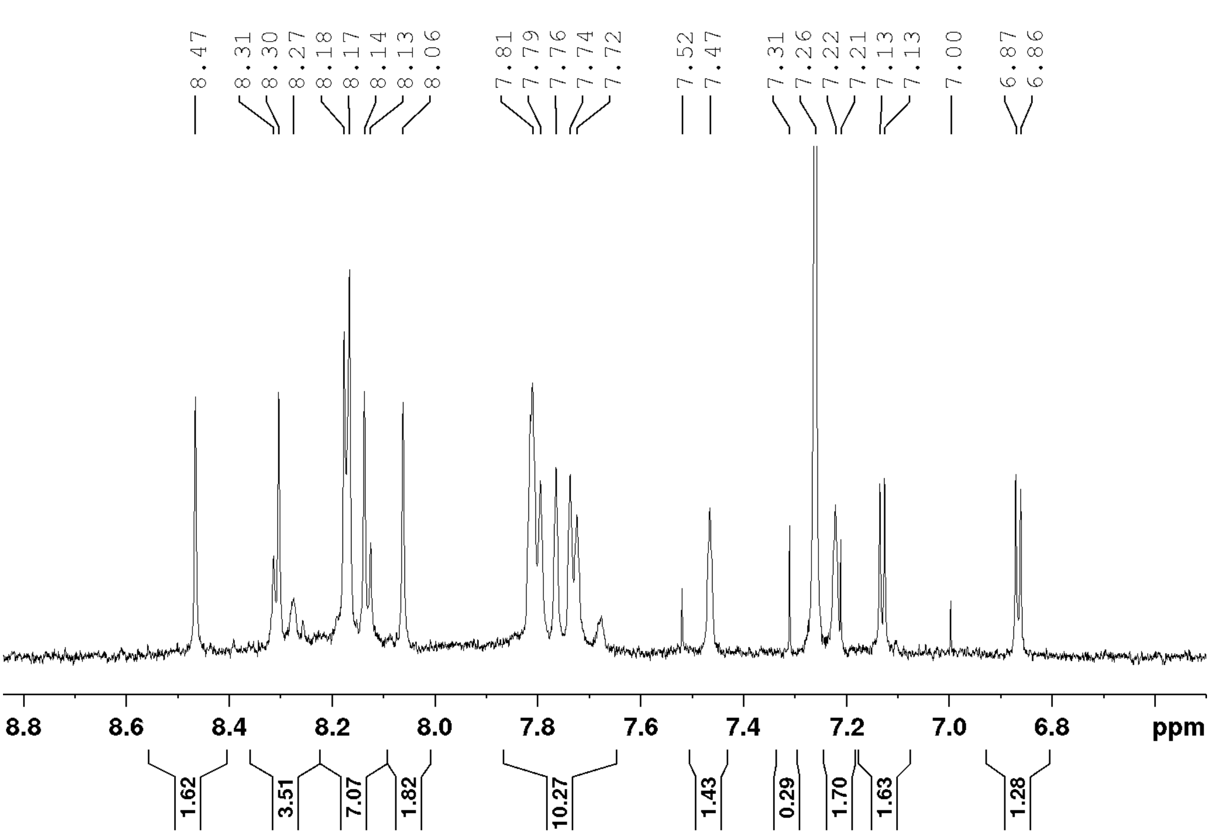

Figure S37 Aromatic region of ^{1}H ($CDCl_3$) NMR of cage **[L1 + 2B1S]**.

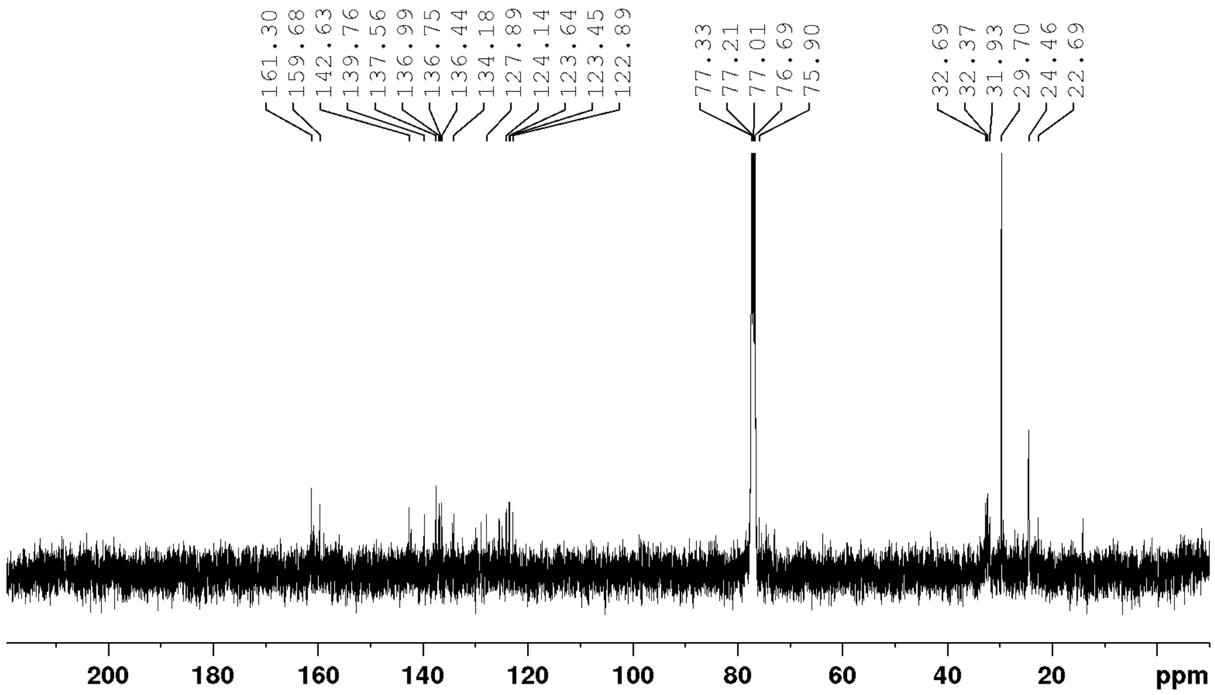

Figure S38 Aromatic region of ^{13}C ($CDCl_3$) NMR of cage **[L1 + 2B1S]**.

Preparation of cage [L1 + 2B1N]

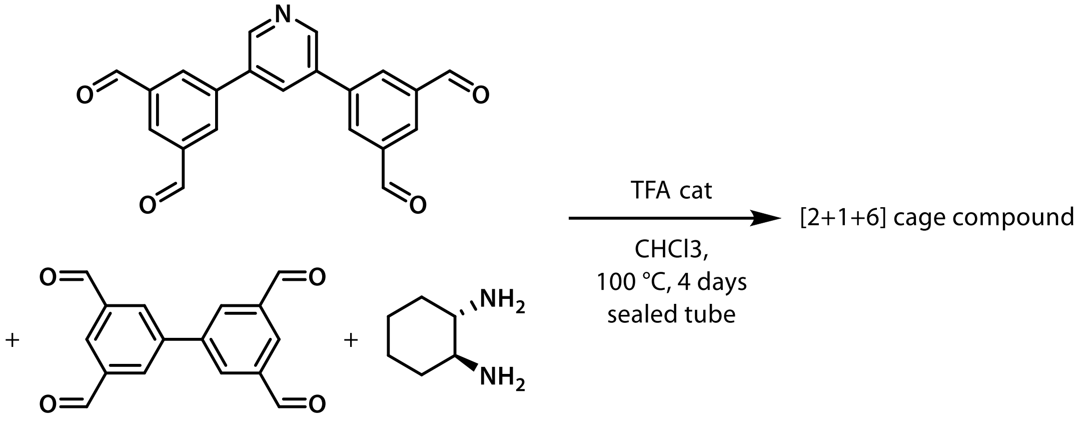

To a stirred solution of 5,5'-(pyridine-3,5-diyl)diisophthalaldehyde (100.0 mg, 0.29 mmol, 2 eq.), [1,1'-biphenyl]-3,3',5,5'-tetracarbaldehyde (38.8 mg, 0.14 mmol, 1 eq.) and (1*S*,2*S*)-cyclohexane-1,2-diamine (98.2 mg, 0.86 mmol, 6 eq.) in $CHCl_3$ (20 mL) in a sealed tube, 2-3 drops of TFA were added. The tube was sealed, the reaction was heated to 100°C and stirred for 4d. The mixture was then diluted with $CHCl_3$ to a 2 mg/mL concentration and the solids were filtered off. The filtrate was concentrated *in vacuo* to ~20 mL, hexane (~40 mL) was added with stirring and the resulting precipitate was collected by filtration or purified by preparative HPLC, which afforded an off-white solid (25.6 mg, 13%). ^{1}H NMR (500 MHz, $CDCl_3$): 8.72 (d, 2H, *J* = 2.2 Hz), 8.50 (d, 2H, *J* = 2.2 Hz), 8.47 (s, 2H), 8.40 (s, 2H), 8.32 (s, 2H), 8.27-8.24 (m, 6H), 8.10 (s, 2H), 8.03 (s, 2H), 7.96 (s, 2H), 7.92-7.88 (m, 6H), 7.72 (s, 2H), 7.60 (s, 2H), 7.40 (s, 2H), 7.17 (s, 2H), 3.54-3.40 (m, 12H), 1.87-1.67 (m, 48H). FT-IR (υ, cm^{-1}): 2925, 2853, 1684, 1647.1448, 1261, 1138, 908, 731. HRMS (ESI^+): *m/z* calcd. for $C_{94}H_{97}N_{14}$ $[M+H]^+$ 1422.8093, found 1422.8037.

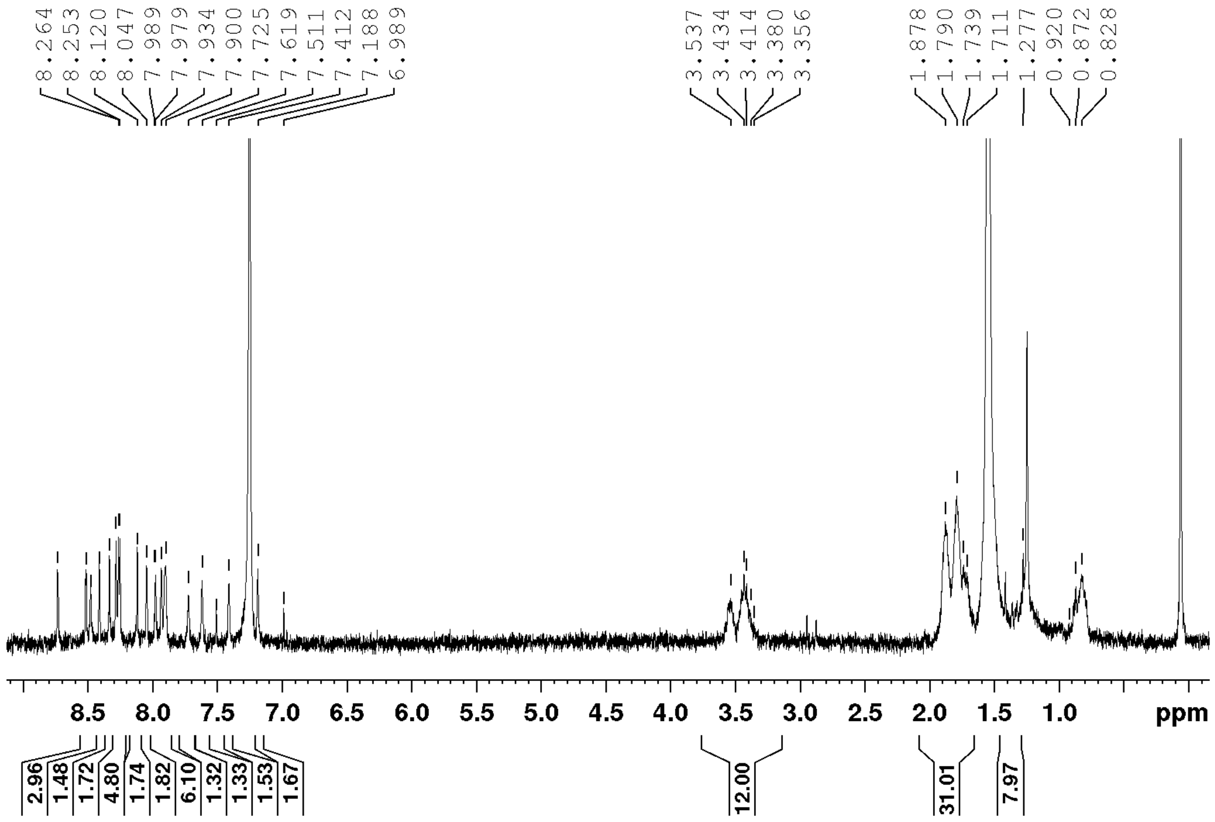

Figure S39 ^{1}H ($CDCl_3$) NMR of cage **[L1 + 2B1N]**.

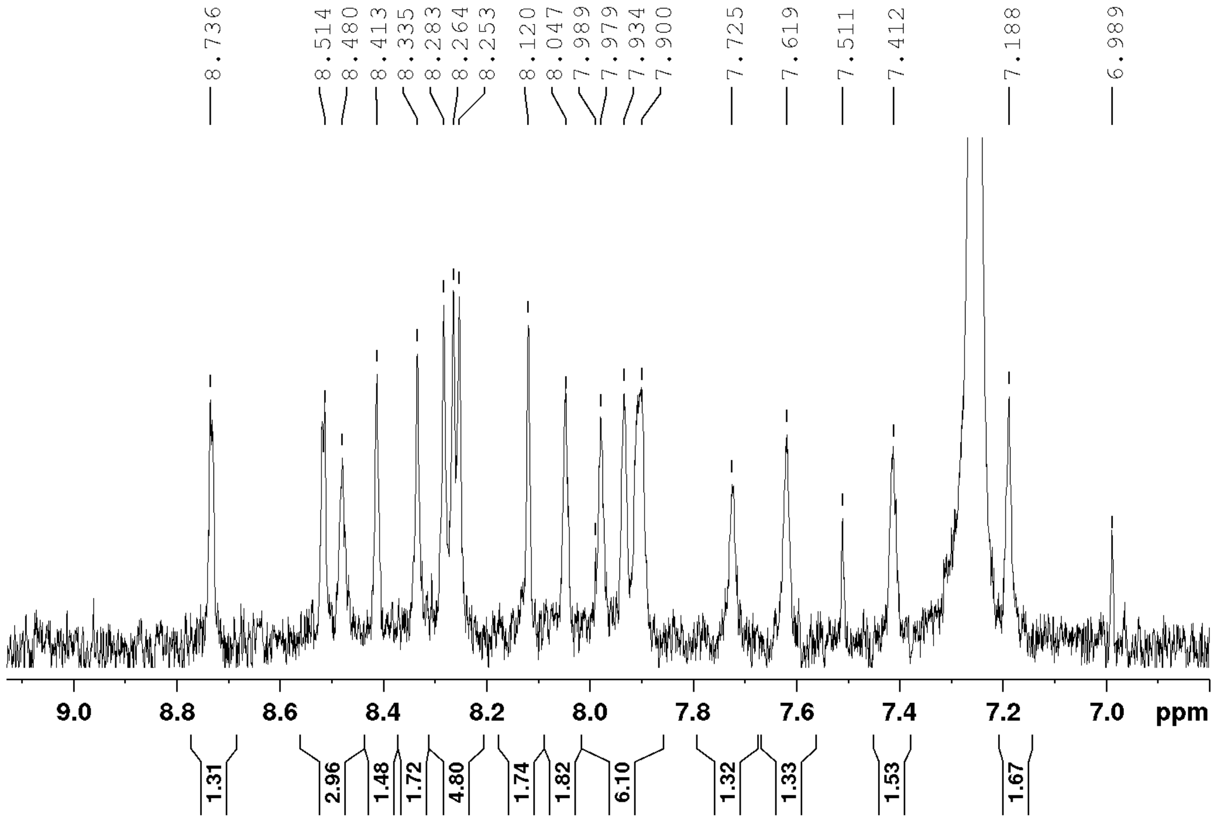

Figure S40 Aromatic region of ^{1}H ($CDCl_3$) NMR of cage **[L1 + 2B1N].**

S4. DOSY NMR spectra and analysis

DOSY spectra, showing diffusion NMR data acquired for cages **[L1 + 2B1]**, **[L1 + 2B1N]** and **[L1 + 2B1S]**, are presented in Figures S41-43. In all three spectra, the horizontal axis is a standard one-dimensional ^{1}H spectrum, with the diffusion information contained in the vertical axis. Signals originating from any one molecule will all have the same diffusion coefficient and are found on the same horizontal line. Where peaks from different species overlap in the ^{1}H spectrum, their net amplitudes will decay multi-exponentially and fitting with a single exponential will give an intermediate diffusion coefficient. In light of this, to obtain precise estimates of diffusion coefficients for the cages, only the isolated, well defined peaks of the aromatic rings are used.[33]

Diffusion coefficients estimated from DOSY spectra are related to hydrodynamic sizes through the Gierer−Wirtz (G−W or SEGWE) modification of the Stokes−Einstein equation (Eqs. 1 and 2 below).[34,35] This approach, where α is the ratio of the solvent molecular weight to the solute molecular weight, is more accurate for smaller species.

$$D = \frac{k_{\mathrm{B}}T\left(\frac{3\alpha}{2} + \frac{1}{1+\alpha}\right)}{6\pi\eta r_H} \qquad (1)$$

$$\alpha = \frac{r_{\mathrm{S}}}{r_H} \qquad (2)$$

Table S6 Experimental diffusion coefficients *D* measured by DOSY NMR (400 MHz, $CDCl_3$, 300 K), theoretical hydrodynamic radii R_H derived following the modified Stokes-Einstein equation (Eqs. 1 and 2), assuming viscosity of chloroform-d as 0.533 mPa s^{-1}, and the theoretical radii of gyration R_G calculated for the modelled structures.

**Cage**	***D* / 10^{-10} m^2 s^{-1}**	**R_H / Å**	**R_G / Å**
**[L1 + 2B1]**	4.2	12.4	7.1
**[L1 + 2B1N]**	3.9	13.1	7.1
**[L1 + 2B1S]**	4.4	11.9	7.0

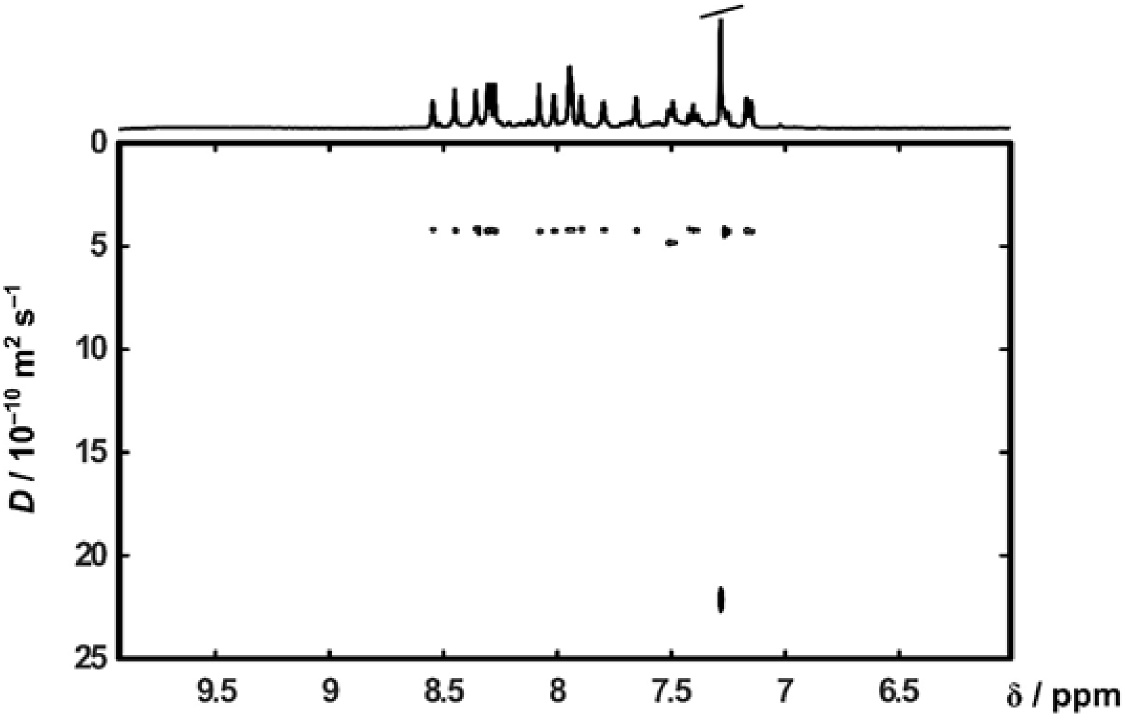

Figure S41 2D DOSY spectrum of **[L1 + 2B1]** in $CDCl_3$ acquired on a Bruker Avance 400 MHz NMR spectrometer at 300 K.

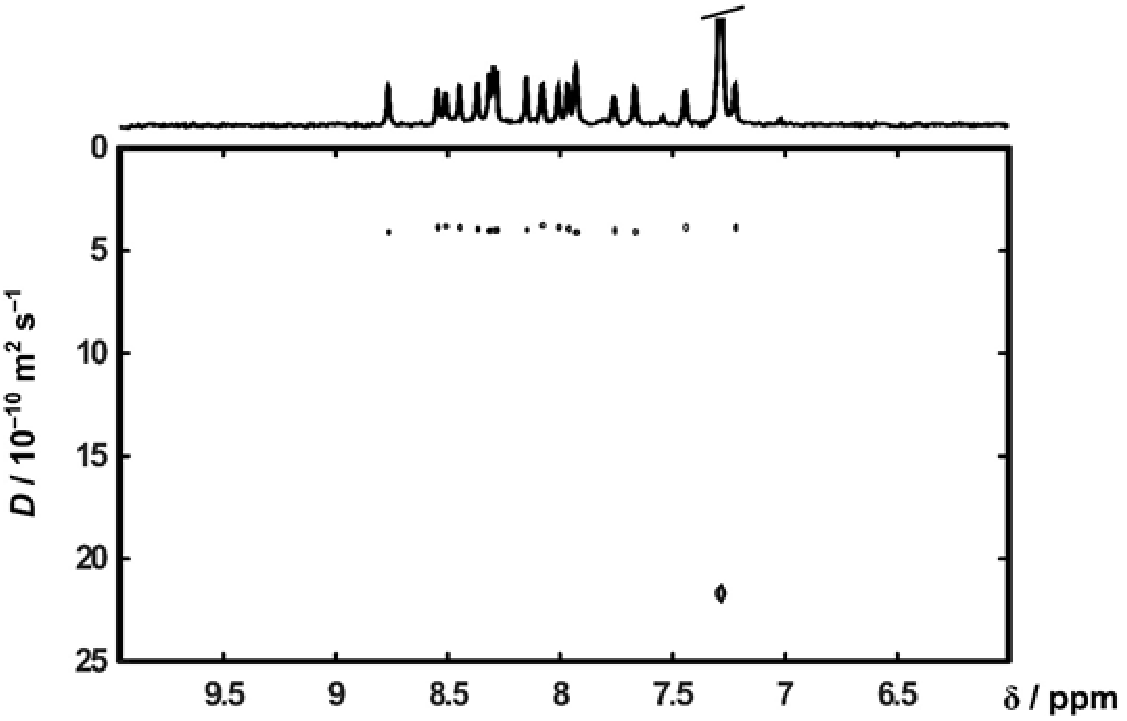

Figure S42 2D DOSY spectrum of **[L1 + 2B1N]** in $CDCl_3$ acquired on a Bruker Avance 400 MHz NMR spectrometer at 300 K.

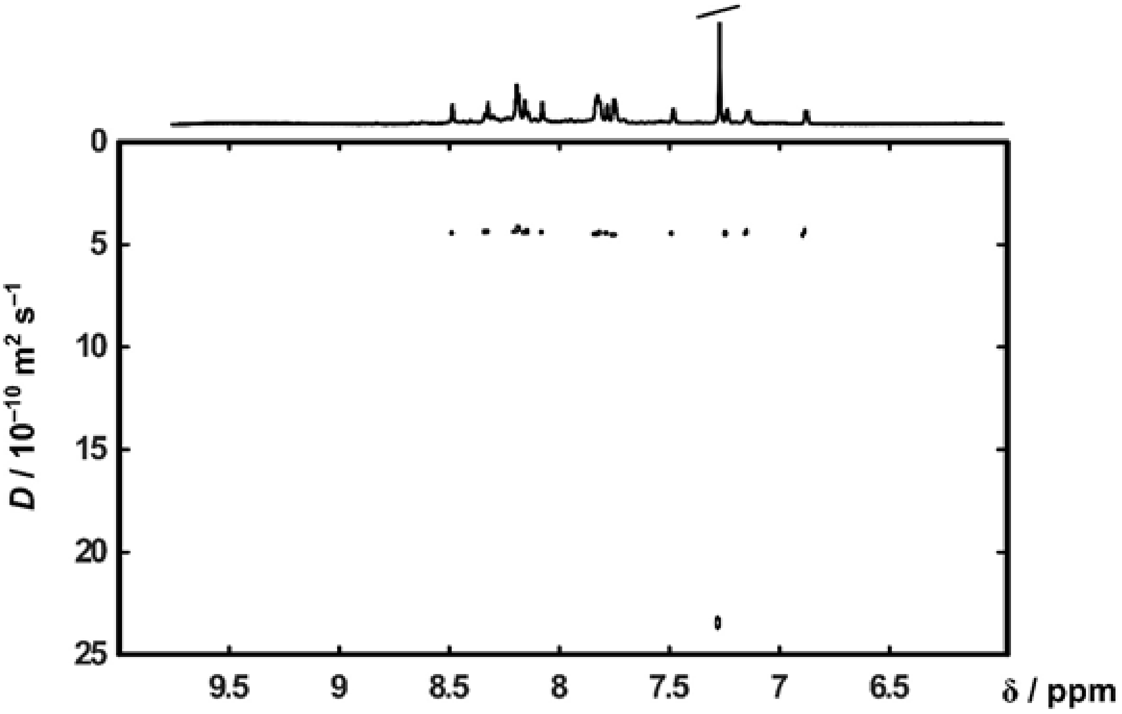

Figure S43: 2D DOSY spectrum of **[L1 + 2B1S]** in $CDCl_3$ acquired on a Bruker Avance 400 MHz NMR spectrometer at 300 K.

S5. IMS-MS spectra and analysis

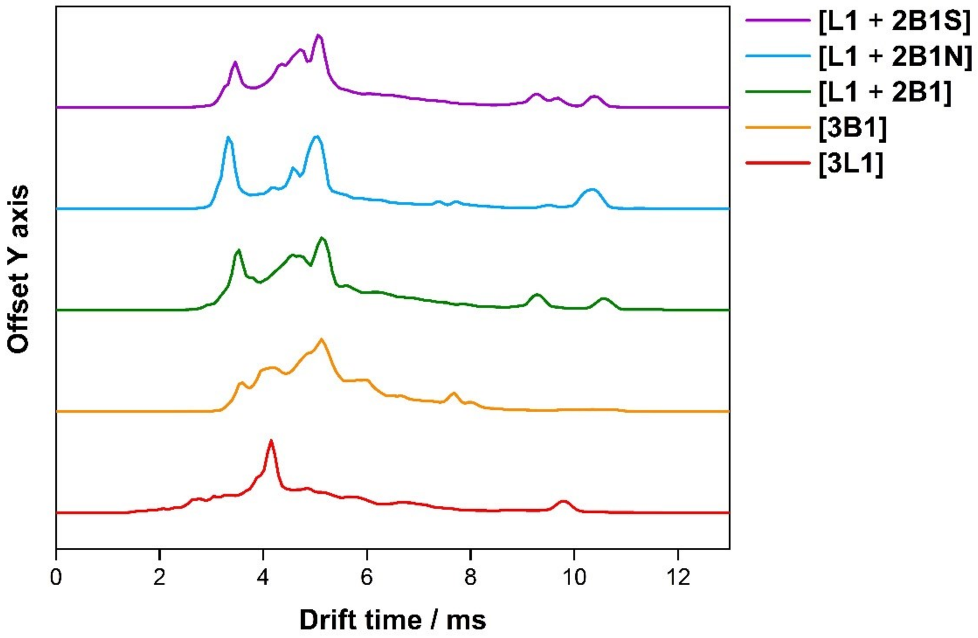

Figure S44 IMS drift times for all products generated by **L1** and **B1**, and mixtures of **L1** with **B1**, **B1N** and **B1S**. Data span the full range of m/z values.

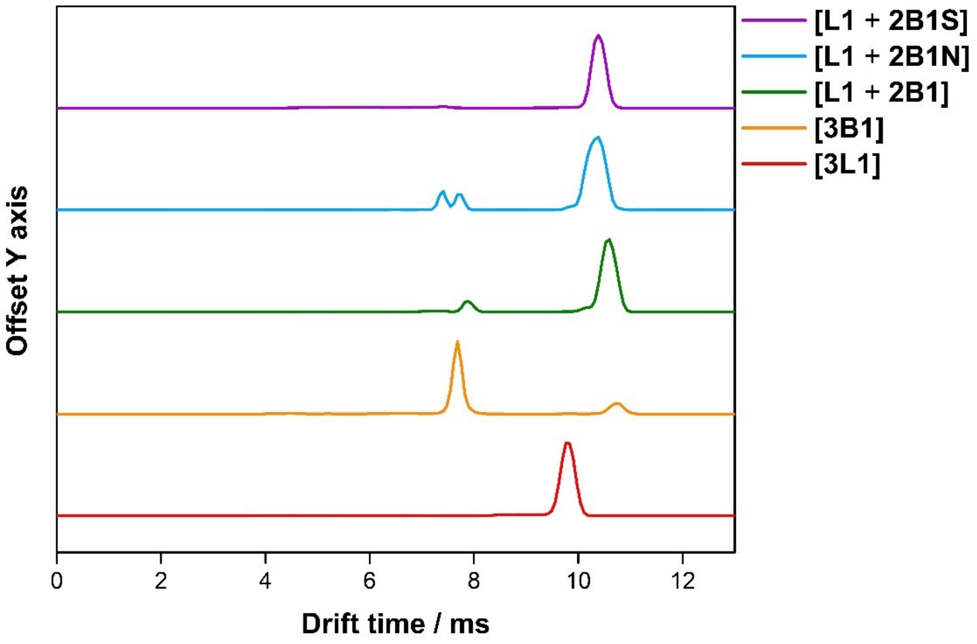

Figure S45 IMS drift times for **[3L1]** (m/z 1266.0 - 1271.8), red, **[3B1]** (m/z 1495.3 - 1498.7), orange, **[L1+2B1]** (m/z 1419.4 - 1424.1), green, **[L1 + 2B1N]** (m/z 1421.4 - 1425.8), blue, and **[L1 + 2B1S]** (m/z 1427.7 - 1439.3), purple.

S6. SC-XRD refinement notes

Table S7 SC-XRD refinement notes for, **[3L1]**, **[3B1-*RS*]**, **[3B2]**.

	**[3L1]**[a]	**[3B1-*RS*]**	**[3B2]**
Crystallisation Solvent	CH_2Cl_2/MeOH	CH_2Cl_2/Hexane	$CHCl_3$/MeOH
Space Group	R$\bar{3}$c	*P*6/*m*	*R*32
Wavelength [Å]	Cu-Kα	0.6889	Mo-Kα
Collection Temperature	150 K	100 K	100 K
Formula	$C_{84}H_{90}N_{12}$, 3(CH_2Cl_2), 3(CH_4O)	$C_{102}H_{102}N_{12}$, 4(CH_2Cl_2)	$C_{120}H_{114}N_{12}$, 7($CHCl_3$), 18.5(H_2O)
Mr	1628.32	1973.42	2894.10
Crystal Size (mm)	0.45 x 0.28 x 0.20	0.19 x 0.09 x 0.08	0.30 x 0.24 x 0.23
Crystal System	Trigonal	Hexagonal	Trigonal
a [Å]	24.993(3)	18.0664(2)	19.6841(11)
c [Å]	24.550(3)	19.5395(3)	32.579(2)
V [Å^3]	13280(4)	5523.15(15)	10932.0(14)
Z	6	2	3
D_{calcd} [g cm^{-3}]	1.222	1.187	1.319
μ [mm^{-1}]	2.244	0.346	0.456
F(000)	5178	2060	4530
2θ range [°]	12.93 – 107.31	2.52 – 51.00	2.69 – 52.73
Reflections collected	19431	38182	41105
Independent reflections, R_{int}	1752, 0.0912	3860, 0.0582	4981, 0.0568
Obs. Data [$I > 2\sigma$]	1150	2115	4500
Data / restraints / parameters	1752 / 0 / 145	3860 / 73 / 214	4981 / 87 / 331
Final R1 values ($I > 2\sigma(I)$)	0.0626	0.0836	0.0779
Final R1 values (all data)	0.1058	0.1121	0.0847
Final wR(F^2) values (all data)	0.1605	0.2412	0.2251
Goodness-of-fit on F^2	1.062	1.498	1.048
Largest difference peak and hole [e.A^{-3}]	0.204 /-0.223	0.497 / -0.293	0.620 / -0.630
Flack parameter			0.06(2)
CCDC	2001201	2001206	2001204

[a] Due to disorder, solvent molecules in the large interconnected 1-D pores could not be modelled. Therefore, a solvent mask, calculated using the SQUEEZE routine in Platon, was used during the final refinement cycles. To account for the solvent mask, 3(CH_2Cl_2) and 3(CH_4O) solvent molecules, per formula unit, were added to the atom count. [b] Due to disorder, solvent molecules in the large interconnected 1-D pores could not be modelled. Therefore, a solvent mask, calculated using the SQUEEZE routine in Platon, was used during the final refinement cycles. To account for the solvent mask, 3.5 $CHCl_3$ solvent molecules were added to the unit cell atom count. All aromatic rings were also refined with constrained geometries (AFIX 66 in SHEXL).

Table S8 SC-XRD refinement notes for **[L1+2B1]**-Hexane, **[L1+2B1]**-Acetone, and **[L1+2B1]**-1,4-Dioxane

	**[L1+2B1]**-Hexane[a]	**[L1+2B1]**-Acetone[a]	**[L1+2B1]**-1,4-Dioxane
Crystallisation Solvent	CH_2Cl_2/hexane	CH_2Cl_2/acetone	CH_2Cl_2/1,4-dioxane
Space Group	$P\bar{1}$	$P\bar{1}$	$P\bar{1}$
Wavelength [Å]	Mo-Kα	Mo-Kα	Mo-Kα
Collection Temperature	100 K	100 K	100 K
Formula	2($C_{96}H_{98}N_{12}$), 10(CH_2Cl_2), H_2O, 1.75(C_6H_{14})	$C_{96}H_{98}N_{12}$, 4.5(C_3H_6O), 0.5(H_2O), 2.5(CH_2Cl_2)	$C_{96}H_{98}N_{12}$, 8.25($C_4H_8O_2$), 2(H_2O)
Mr	3857.79	1905.05	2181.74
Crystal Size (mm)	0.10 x 0.10 x 0.08	0.20 x 0.04 x 0.04	0.19 x 0.09 x 0.09
Crystal System	Triclinic	Triclinic	Triclinic
a [Å]	20.698(3)	15.423(3)	16.038(4)
b [Å]	20.865(3)	17.147(3)	18.077(5)
c [Å]	28.627(4)	22.886(4)	23.415(6)
α [°]	104.802(4)	87.085(4)	87.286(7)
β [°]	102.225(4)	74.544(4)	72.600(6)
γ [°]	107.903(4)	70.574(4)	68.939(7)
V [Å^3]	10793(2)	5496.8(17)	6031(3)
Z	2	2	2
D_{calcd} [g cm^{-3}]	1.187	1.151	1.201
μ [mm^{-1}]	0.309	0.188	0.080
F(000)	4067	2029	2346
2θ range [°]	2.18 – 41.63	1.85 – 46.51	3.65 – 41.20
Reflections collected	91925	58264	30500
Independent reflections, R_{int}	22514, 0.1059	15764, 0.0874	12097, 0.0701
Obs. Data [$I > 2\sigma$]	13542	7521	7674
Data / restraints / parameters	22514 / 1973 / 2309	15764 / 1036 / 934	12097 / 1249 / 1483
Final R1 values ($I > 2\sigma(I)$)	0.1570	0.0841	0.1197
Final R1 values (all data)	0.2034	0.1534	0.1721
Final wR(F^2) values (all data)	0.3728	0.2309	0.2679
Goodness-of-fit on F^2	1.906	1.051	2.034
Largest difference peak and hole [e.A^{-3}]	0.924 / -0.733	0.621 / -0.480	0.695 / -0.597
CCDC	2001208	2001205	2001203

[a] Due to severe disorder, solvent masks were used during the final refinement cycles and the structures were refined with a group rigid-bond restraints (RIGU in SHELX).

Table S9. SC-XRD refinement notes for, **[L1+2B1S].**

	**[L1+2B1S]**
Crystallisation Solvent	CH2Cl2/Hexane
Space Group	*I*2/*a*
Wavelength [Å]	Mo-Kα
Collection Temperature	100 K
Formula	$C_{92}H_{94}N_{12}S_2$, 1.7(CH_2Cl_2), C_6H_{14}
Mr	1662.45
Crystal Size (mm)	0.40 x 0.31 x 0.28
Crystal System	Monoclinic
a [Å]	25.492(3)
b [Å]	11.3053(12)
c [Å]	32.839(5)
β [°]	103.978(3)
V [Å^3]	9184(2)
Z	4
D_{calcd} [g cm^{-3}]	1.202
μ [mm^{-1}]	0.210
F(000)	3534
2θ range [°]	3.65 – 54.97
Reflections collected	55213
Independent reflections, R_{int}	10533, 0.0600
Obs. Data [$I > 2\sigma$]	7829
Data / restraints / parameters	10533 / 10 / 555
Final R1 values ($I > 2\sigma(I)$)	0.0521
Final R1 values (all data)	0.0749
Final wR(F^2) values (all data)	0.1542
Goodness-of-fit on F^2	1.034
Largest difference peak and hole [e.A^{-3}]	0.464 / -0.437
CCDC	2001202

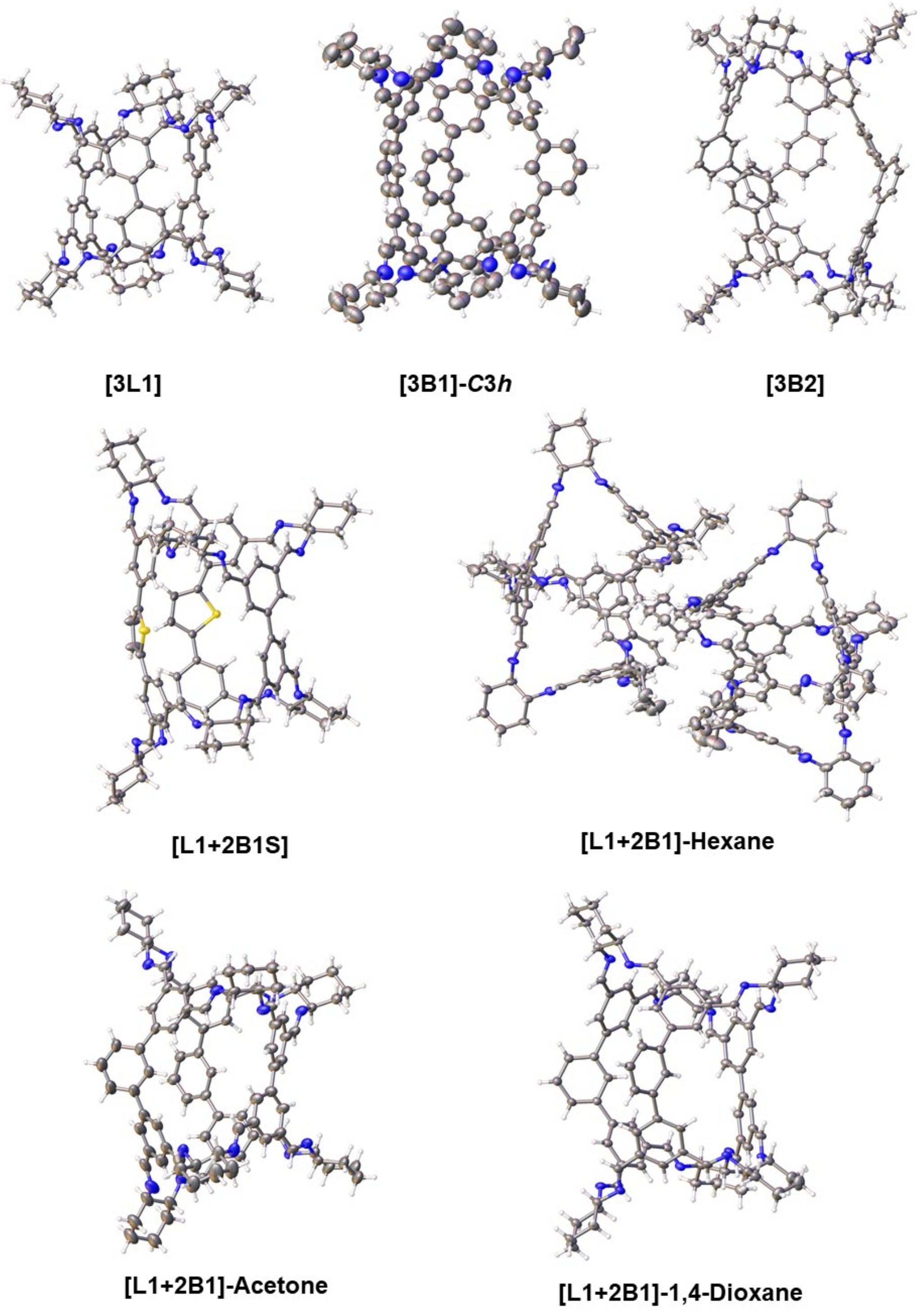

Figure S46 Displacement ellipsoid plots from the single crystal structures; **[3L1]**, **[3B1-*RS*]-C3*h***, **[3B2]**, **[L1+2B1]-Hexane**, **[L1+2B1]-Acetone**, **[L1+2B1]-1,4-Dioxane**, and **[L1+2B1S]**; ellipsoid are displayed at 50% probability level and all H atoms are omitted for clarity. Whole cage molecules are shown for the structures; **[3L1]**, **[3B1-*RS*]-C3*h***, **[3B2]**, and in **[L1+2B1]-Hexane** there are two cage molecules in the asymmetric unit. Ellipsoid plots are not shown on the same scale. C = grey, H = white, N = blue, S = yellow.

S7. References

[1] O. A. Blackburn, B. J. Coe, M. Helliwell, J. Raftery, *Organometallics* **2012**, *31*, 5307–5320.

[2] A. S. Demir, Ö. Reis, M. Emrullahoglu, *J. Org. Chem.* **2003**, *68*, 10130–10134.

[3] A. G. Slater, M. A. Little, A. Pulido, S. Y. Chong, D. Holden, L. Chen, C. Morgan, X. Wu, G. Cheng, R. Clowes, M. E. Briggs, T. Hasell, K. E. Jelfs, G. M. Day, A. I. Cooper, *Nat. Chem.* **2017**, *9*, 17–25.

[4] A. Jerschow, N. Müller, *J. Magn. Reson.* **1997**, *125*, 372–375.

[5] T. M. Barbosa, R. Rittner, C. F. Tormena, G. A. Morris, M. Nilsson, *RSC Adv.* **2016**, *6*, 95173–95176.

[6] I. Swan, M. Reid, P. W. A. Howe, M. A. Connell, M. Nilsson, M. A. Moore, G. A. Morris, *J. Magn. Reson.* **2015**, *252*, 120–129.

[7] M. Nilsson, *J. Magn. Reson.* **2009**, *200*, 296–302.

[8] G. M. Sheldrick, *Univ. Göttingen, Ger.* **1996**.

[9] G. Winter, D. G. Waterman, J. M. Parkhurst, A. S. Brewster, R. J. Gildea, M. Gerstel, L. Fuentes-Montero, M. Vollmar, T. Michels-Clark, I. D. Young, N. K. Sauter, G. Evans, *Acta Cryst. D* **2018**, *74*, 85–97.

[10] G. M. Sheldrick, *Acta Cryst. A* **2015**, *71*, 3–8.

[11] G. M. Sheldrick, *Acta Cryst. C* **2015**, *71*, 3–8.

[12] O. V. Dolomanov, L. J. Bourhis, R. J. Gildea, J. A. K. Howard, H. Puschmann, *J. Appl. Crystallogr.* **2009**, *42*, 339–341.

[13] V. Santolini, M. Miklitz, E. Berardo, K. E. Jelfs, *Nanoscale* **2017**, *9*, 5280–5298.

[14] K. Roos, C. Wu, W. Damm, M. Reboul, J. M. Stevenson, C. Lu, M. K. Dahlgren, S. Mondal, W. Chen, L. Wang, R. Abel, R. A. Friesner, E. D. Harder, *J. Chem. Theory Comput.* **2019**, *15*, 1863–1874.

[15] J. P. Perdew, K. Burke, M. Ernzerhof, *Phys. Rev. Lett.* **1996**, *77*, 3865–3868.

[16] J. VandeVondele, J. Hutter, *J. Chem. Phys.* **2007**, *127*, 114105.

[17] S. Goedecker, M. Teter, J. Hutter, *Phys. Rev. B* **1996**, *54*, 1703–1710.

[18] C. Hartwigsen, S. Goedecker, J. Hutter, *Phys. Rev. B* **1998**, *58*, 3641–3662.

[19] M. Krack, *Theor. Chem. Acc.* **2005**, *114*, 145–152.

[20] G. Lippert, M. Parrinello, J. Hutter, *Mol. Phys.* **1997**, *92*, 477–488.

[21] S. Grimme, J. Antony, S. Ehrlich, H. Krieg, *J. Chem. Phys.* **2010**, *132*, 154104.

[22] S. Grimme, S. Ehrlich, L. Goerigk, *J. Comput. Chem.* **2011**, *32*, 1456–1465.

[23] J. VandeVondele, M. Krack, F. Mohamed, M. Parrinello, T. Chassaing, J. Hutter, *Comput. Phys. Commun.* **2005**, *167*, 103–128.

[24] J. Hutter, M. Iannuzzi, F. Schiffmann, J. VandeVondele, *Wiley Interdiscip. Rev. Comput. Mol. Sci.* **2014**, *4*, 15–25.

[25] Y. Zhao, D. G. Truhlar, *Theor. Chem. Acc.* **2008**, *120*, 215–241.

[26] A. D. McLean, G. S. Chandler, *J. Chem. Phys.* **1980**, *72*, 5639–5648.

[27] M. J. Frisch, J. A. Pople, J. S. Binkley, *J. Chem. Phys.* **1984**, *80*, 3265–3269.

[28] M. J. Frisch, G. W. Trucks, H. B. Schlegel, G. E. Scuseria, M. A. Robb, J. R. Cheeseman, G. Scalmani, V. Barone, G. A. Petersson, H. Nakatsuji, X. Li, M. Caricato, A. V Marenich, J. Bloino, B. G. Janesko, R. Gomperts, B. Mennucci, H. P. Hratchian, J. V Ortiz, A. F. Izmaylov, J. L. Sonnenberg, D. Williams-Young, F. Ding, F. Lipparini, F. Egidi, J. Goings, B. Peng, A. Petrone, T. Henderson, D. Ranasinghe, V. G. Zakrzewski, J. Gao, N. Rega, G. Zheng, W. Liang, M. Hada, M. Ehara, K. Toyota, R. Fukuda, J. Hasegawa, M. Ishida, T. Nakajima, Y. Honda, O. Kitao, H. Nakai, T. Vreven, K. Throssell, J. A. Montgomery Jr., J. E. Peralta, F. Ogliaro, M. J. Bearpark, J. J. Heyd, E. N. Brothers, K. N. Kudin, V. N. Staroverov, T. A. Keith, R. Kobayashi, J. Normand, K. Raghavachari, A. P. Rendell, J. C. Burant, S. S. Iyengar, J. Tomasi, M. Cossi, J. M. Millam, M. Klene, C. Adamo, R. Cammi, J. W. Ochterski, R. L. Martin, K. Morokuma, O. Farkas, J. B. Foresman, D. J. Fox, *Gaussian, Inc., Wallingford CT* **2016**.

[29] P. Virtanen, R. Gommers, T. E. Oliphant, M. Haberland, T. Reddy, D. Cournapeau, E. Burovski, P. Peterson, W. Weckesser, J. Bright, S. J. van der Walt, M. Brett, J. Wilson, K. J. Millman, N. Mayorov, A. R. J. Nelson, E. Jones, R. Kern, E. Larson, C. J. Carey, İ. Polat, Y. Feng, E. W. Moore, J. VanderPlas, D. Laxalde, J. Perktold, R. Cimrman, I. Henriksen, E. A. Quintero, C. R. Harris, A. M. Archibald, A. H. Ribeiro, F. Pedregosa, P. van Mulbregt, *Nat. Methods* **2020**, *17*, 261–272.

[30] S. van der Walt, S. C. Colbert, G. Varoquaux, *Comput. Sci. Eng.* **2011**, *13*, 22–30.

[31] A. Stukowski, *Model. Simul. Mater. Sci. Eng.* **2010**, *18*, 015012.

[32] B. Mondal, A. K. Ghosh, P. S. Mukherjee, *J. Org. Chem.* **2017**, *82*, 7783–7790.

[33] V. Abet, R. Evans, F. Guibbal, S. Caldarelli, R. Rodriguez, *Angew. Chemie Int. Ed.* **2014**, *53*, 4862–4866.

[34] R. Evans, Z. Deng, A. K. Rogerson, A. S. McLachlan, J. J. Richards, M. Nilsson, G. A. Morris, *Angew. Chemie Int. Ed.* **2013**, *52*, 3199–3202.

[35] R. Evans, G. Dal Poggetto, M. Nilsson, G. A. Morris, *Anal. Chem.* **2018**, *90*, 3987–3994.
